# *RASA2* ablation in T cells boosts antigen sensitivity and long-term function

**DOI:** 10.1038/s41586-022-05126-w

**Published:** 2022-08-24

**Authors:** Julia Carnevale, Eric Shifrut, Nupura Kale, William A. Nyberg, Franziska Blaeschke, Yan Yi Chen, Zhongmei Li, Sagar P. Bapat, Morgan E. Diolaiti, Patrick O’Leary, Shane Vedova, Julia Belk, Bence Daniel, Theodore L. Roth, Stefanie Bachl, Alejandro Allo Anido, Brooke Prinzing, Jorge Ibañez-Vega, Shannon Lange, Dalia Haydar, Marie Luetke-Eversloh, Maelys Born-Bony, Bindu Hegde, Scott Kogan, Tobias Feuchtinger, Hideho Okada, Ansuman T. Satpathy, Kevin Shannon, Stephen Gottschalk, Justin Eyquem, Giedre Krenciute, Alan Ashworth, Alexander Marson

**Affiliations:** 1 Gladstone–UCSF Institute of Genomic Immunology, San Francisco, CA USA; 2 Department of Medicine, University of California, San Francisco, San Francisco, CA USA; 3 UCSF Helen Diller Family Comprehensive Cancer Center, University of California, San Francisco, San Francisco, CA USA; 4 Parker Institute for Cancer Immunotherapy, University of California, San Francisco, San Francisco, CA USA; 5 Department of Microbiology and Immunology, University of California, San Francisco, San Francisco, CA USA; 6 Diabetes Center, University of California San Francisco, San Francisco, CA USA; 7 Department of Laboratory Medicine, University of California, San Francisco, San Francisco, CA USA; 8 Department of Pathology, Stanford University, Stanford, CA USA; 9 Department of Bone Marrow Transplantation and Cellular Therapy, St Jude Children’s Research Hospital, Memphis, TN USA; 10 Department of Pediatric Hematology, Oncology and Stem Cell Transplantation, Dr von Hauner Children’s Hospital, University Hospital, LMU, Munich, Germany; 11 Department of Neurosurgery, University of California, San Francisco, San Francisco, CA USA; 12 Parker Institute for Cancer Immunotherapy, Stanford University, Stanford, CA USA; 13 Department of Pediatrics, University of California, San Francisco, San Francisco, CA USA; 14 Chan Zuckerberg Biohub, San Francisco, CA USA; 15 Innovative Genomics Institute, University of California, Berkeley, Berkeley, CA USA; 16 Present Address: The School of Neurobiology, Biochemistry and Biophysics, The George S. Wise Faculty of Life Sciences, Tel Aviv University, Tel Aviv, Israel; 17 Present Address: Department of Pathology Sackler Faculty of Medicine, Tel Aviv University, Tel Aviv, Israel; 18 Present Address: Varda and Boaz Dotan Center for Advanced Therapies, Tel Aviv Sourasky Medical Center, Tel Aviv, Israel

**Keywords:** Immunotherapy, Cancer immunotherapy

## Abstract

The efficacy of adoptive T cell therapies for cancer treatment can be limited by suppressive signals from both extrinsic factors and intrinsic inhibitory checkpoints^1,2^. Targeted gene editing has the potential to overcome these limitations and enhance T cell therapeutic function^3–10^. Here we performed multiple genome-wide CRISPR knock-out screens under different immunosuppressive conditions to identify genes that can be targeted to prevent T cell dysfunction. These screens converged on RASA2, a RAS GTPase-activating protein (RasGAP) that we identify as a signalling checkpoint in human T cells, which is downregulated upon acute T cell receptor stimulation and can increase gradually with chronic antigen exposure. RASA2 ablation enhanced MAPK signalling and chimeric antigen receptor (CAR) T cell cytolytic activity in response to target antigen. Repeated tumour antigen stimulations in vitro revealed that RASA2-deficient T cells show increased activation, cytokine production and metabolic activity compared with control cells, and show a marked advantage in persistent cancer cell killing. RASA2-knockout CAR T cells had a competitive fitness advantage over control cells in the bone marrow in a mouse model of leukaemia. Ablation of RASA2 in multiple preclinical models of T cell receptor and CAR T cell therapies prolonged survival in mice xenografted with either liquid or solid tumours. Together, our findings highlight RASA2 as a promising target to enhance both persistence and effector function in T cell therapies for cancer treatment.

## Abstract (web summary)

Genome-wide CRISPR screens, biochemical studies and animal models show that *RASA2* has a key role in regulating T cell function and has potential as a genetic target for enhancing anti-tumour immunity.

## Main

CAR T cells have been transformative in a subset of aggressive haematological malignancies, and T cell receptor (TCR)-transgenic T cells (TCR T cells) have shown promising results in early-phase clinical studies for solid tumours^1^. However, many cancers, especially solid tumours, do not respond to current T cell therapies or rapidly progress after the initial response. Within the tumour mass, the immunosuppressive microenvironment poses a substantial barrier to the efficacy of anti-tumour immunity^2,11^. In addition, persistent exposure to antigen can lead to T cell dysfunction, highlighting the need to balance effector function and long-term persistence in engineered T cells^3,12^. Targeted manipulation of select genes is being tested as a strategy to boost the efficacy of adoptive T cell therapies^5–7^. However, the optimal gene targets in human T cells have not been explored systematically. Large-scale CRISPR screens can accelerate the discovery of genetic perturbations that can boost the efficacy of engineered T cells^3,8–10^. We previously developed a discovery platform in primary human T cells and applied it to identify novel genetic regulators of T cell proliferation^13^. Here we describe unbiased genetic screens performed under several immunosuppressive conditions commonly encountered in the tumour microenvironment (TME) that uncovered ablation of the *RASA2* gene as a strategy for T cells to overcome multiple inhibitory cues. We find that ablation of RASA2 enhances sensitivity to antigen and improves both effector function and persistence of CAR T and TCR T cells. Finally, we show that RASA2-ablation in antigen-specific T cells can enhance tumour control and extend survival in multiple preclinical models of liquid and solid tumours.

## CRISPR screens converge on RASA2

The suppressive TME and T cell intrinsic checkpoints can impinge on the efficacy of engineered T cells targeting solid tumours^14^. We developed a systematic approach to identify genetic perturbations that could render T cells resistant to a range of inhibitory signals encountered in the TME. We previously used CGS-21680, an adenosine agonist^13^, to simulate elevated adenosine A_2A_ inhibitory signalling in response to high levels of adenosine in the hypoxic TME^15^. Here we extended this strategy to model multiple challenges to T cell function in the TME. To model intrinsic checkpoint signals, we focused on inhibitors of calcium and calcineurin signaling (tacrolimus and cyclosporine), which is a critical pathway for T cell activation that is frequently suppressed in tumour-infiltrating T cells^16^. To mimic a prominent extrinsic inhibitory signal in the TME, we used TGFβ, a canonical suppressive cytokine that limits T cell function within tumours^17^. Finally, as T regulatory cells (T_reg_ cells) are important mediators of T cell dysfunction in multiple tumour types^18^, we adapted our screening platform to assay cell–cell interactions and thereby reveal genes that confer resistance to suppression of effector T cells by T_reg_ cells.

**Fig. 1:**
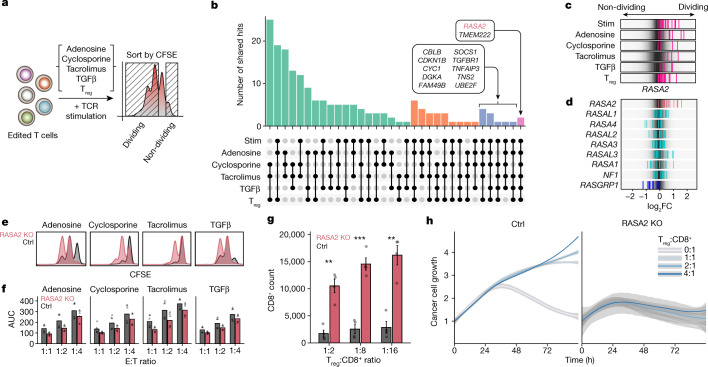
Multiple genome-wide CRISPR screens in primary human T cells identify RASA2 as a modulator of resistance to immunosuppressive conditions. **a**, Schematic of genome-wide screens for resistance gene targets in human T cells. **b**, Top shared gene hits (*z*-score >1.5) between 5 (blue) and all 6 (pink) of the screens are labelled. Bar height is the number of shared genes among the screens, connected by dots in the lower panel (*n* = 4 human donors for stimulated (stim) and T_reg_ cell screens, *n* = 2 for adenosine, cyclosporine and tacrolimus, and *n* = 1 for the TGFβ screen). **c**,**d**, log_2_ fold change (FC) for individual guide RNAs (vertical lines); background shows the overall guide distribution in greyscale. **c**, Guides targeting *RASA2* (pink) across all suppressive conditions. **d**, Guides targeting RasGAP family members other than RASA2 were not enriched consistently in either direction, whereas guides targeting the RasGEF RASGRP1 were depleted from dividing cells as expected. **e**, Distribution of CFSE staining in RASA2-KO versus control (Ctrl; non-targeting guide RNA) T cells across all suppressive conditions. **f**, Cancer cell growth during in vitro cancer cell-killing assay under suppressive conditions. AUC, area under the growth curve. *n* = 2 donors in triplicate, shape denotes donor. **g**, Suppression assay confirms that *RASA2* ablation rendered T cells resistant to T_reg_ cell suppression of proliferation in vitro. Bars show the CD8^+^ cell count 4 days after stimulation (*n* = 4 donors per group; mean ± s.e.m.; **P < 0.01 and ***P < 0.001, two-sided paired Student’s *t*-test). **h**, *RASA2* ablation rendered T cells resistant to T_reg_ cell suppression compared with control T cells in an in vitro cancer cell-killing assay for one representative donor out of four (summary statistics shown in Extended Data Fig. 2g). Line is the mean and shaded area is 95% confidence interval for 3 technical replicates. Source data

**Extended Data Fig. 1:**
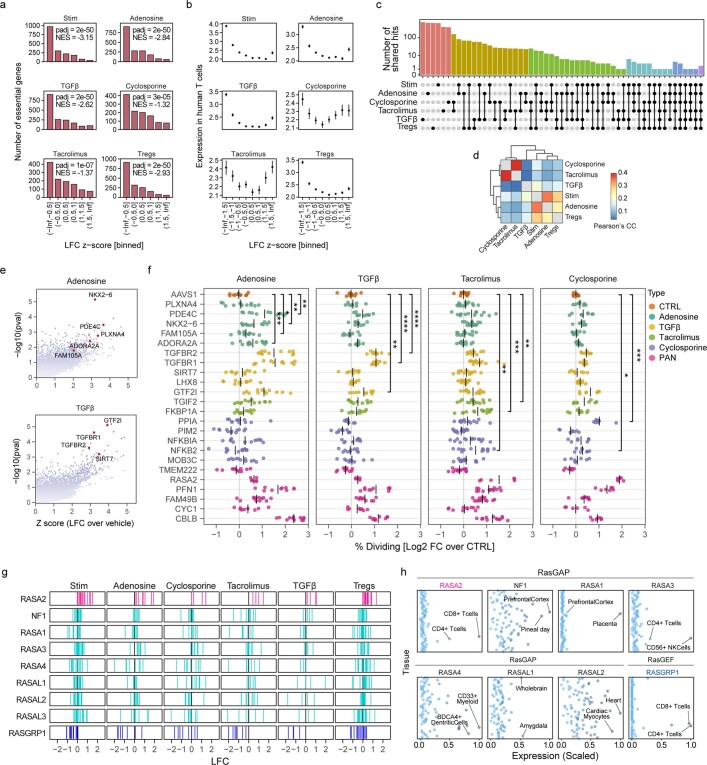
Multiple genome-wide CRISPR screens for T cell resistance. **a**, Dropout of essential genes^19^ across screen conditions. X-axis is scaled and binned log2 fold change of CFSE low over CFSE high cells in each screen, Y-axis is the number of essential genes in each bin. As expected, essential genes tend to have a negative LFC in these CRISPR KO screens of primary human T cells (n = 4 human donors for Stim and Tregs screens, n = 2 for Adenosine, Cyclosporine and Tacrolimus screens, and n = 1 for the TGFβ screen. Normalized Enrichment Score (NES) and adjusted p-value by GSEA and two-sided permutation test). **b**, Screen hits are expressed in human T cells. X-axis is scaled and binned log2 fold change in each screen, Y-axis shows the expression in activated human T cells^20^. Both negative and positive hits tended to be highly expressed in human T cells, suggesting these pooled KO screens point to relevant T cell biology (n = 4 human donors for Stim and Tregs screens, n = 2 for Adenosine, Cyclosporine and Tacrolimus screens, and n = 1 for the TGFβ screen, dots are mean +/− SEM). **c**, Shared hits (y-axis) (z-score > 1.5, methods) across the screen conditions (x-axis) including hits unique to each individual screen. Shared hits for each subset are detailed in Supplementary Table 1. **d**, Heatmap of the pairwise Pearson’s correlation coefficient for gene-level z-scores for all screen conditions. **e**, Volcano plots showing p-value (MAGeCK RRA one-sided test and methods) on the y-axis and gene-level z-scores on the x-axis, comparing highly dividing cells in each suppressive condition to highly dividing in the vehicle condition. Highlighted are genes found to be specific to adenosine and TGFβ screens, selected for further validation. **f**, Gene targets from screens were selected as either general (PAN) or more specific to certain suppressive contexts and were knocked out individually in T cells. CFSE stained, Cas9 RNP-electroporated edited T cells were stimulated and cultured in the different suppressive conditions. Percent of cells proliferating for each gene KO compared to control cells are displayed for each suppressive condition (n = 2 donors, 2 sgRNAs per gene target in triplicates. We highlighted gene KOs found to confer significant resistance in predicted conditions (adenosine, TGFB, and calcium/calcineurin inhibitors – Tacrolimus, and Cyclosporine), using a cut-off of FDR adjusted p-value < 0.05. For clarity, displayed are the significant p-values for gene targets according to their suppressive screen condition of origin, but results for all genes across conditions are detailed in Supplementary Table 3). As expected, ADORA2A, TGFBR1 and TGFBR2, FKBP1A, and PPIA KOs conferred resistance in the adenosine, TGFB, Tacrolimus, and Cyclosporine conditions, respectively. PDE4C and NKX2-6 KOs were found to confer relatively selective resistance in the adenosine condition, and NFKB2 KO was found to increase resistance in the calcineurin inhibitor (tacrolimus and cyclosporine) conditions. TMEM222, while scoring very highly in the screens, did not increase proliferative advantage in this arrayed validation (dots are individuals replicates, black vertical lines are the mean, *p < 0.05, **p < 0.01, ***p < 0.001 and ***p < 0.0001 for two-sided unpaired Student’s t-test). **g**, Log fold change (LFC) of guides targeting RasGAP genes or the RasGEF RASGRP1, across the different suppressive screen conditions shown here. **h**, Expression levels (scaled to minimum of 0 and a maximum of 1) of the RasGAP family members available in the BioGPS dataset^27^, including RASA2, across healthy human tissues revealed RASA2 as selectively expressed in CD8^+^/4^+^ human T cells. Data also shown for RASGRP1, a RasGEF with defined roles in TCR signaling and an expression pattern strikingly similar to that of RASA2. Source data

To identify regulators of resistance to these suppressive conditions, we applied single guide RNA (sgRNA) lentiviral infection with Cas9 electroporation (SLICE) of pooled genome-wide CRISPR-knockout (KO) screens in primary human T cells^13^. Here, we analysed a total of six different genome-wide screens in primary human T cells across multiple independent donors and suppressive conditions (Fig. 1a). In each of these conditions, we identified gene targets that promoted T cell proliferation by flow cytometry-based cell sorting to identify sgRNAs enriched in the dividing cells (low carboxyfluorescein succinimidyl ester (CFSE) staining) over those in non-dividing cells (high CFSE staining) after the cells were re-stimulated. As expected, guides targeting essential genes were depleted in highly dividing cells compared with non-dividing cells across screen conditions, and gene hits were correlated with higher expression in human T cells^19,20^ (Extended Data Fig. 1a,b). Analysis of gene hits enriched in the highly dividing compared with non-dividing cells that were shared between all screens converged on two candidate resistance target genes: *TMEM222* and *RASA2* (Fig. 1b, Extended Data Fig. 1c and Supplementary Table 1). Cross-comparison of screen hits highlighted the extent of shared hits between screens performed with similar suppressive cues (for example, tacrolimus and cyclosporine) (Extended Data Fig. 1d). Comparative analysis of the sgRNAs in highly dividing cells across screens nominated hits selective for each suppressive condition, as well as those conferring more general resistance profiles for additional validation (Supplementary Table 2). A subset of gene hits was more specific for individual screens—for example, *ADORA2A* and *TGFBR1* scored highly in the adenosine and TGFβ conditions, respectively (Extended Data Fig. 1e). Arrayed validation of selected genes by CRISPR-mediated knockout confirmed the potential of these unbiased screens to discover novel regulators of T cell resistance to TME-related suppressive cues (Extended Data Fig. 1e and Supplementary Table 3). For example, targeting *PDE4C* or *NKX2-6* rendered T cells resistant to adenosine suppression, whereas *NFKB2* KO conferred resistance to the calcineurin inhibitors tacrolimus and cyclosporine. Notably, we observed cross-talk between hits for TGFβ and those for adenosine resistance, supporting previously described interplay between these immunosuppressive signals^21,22^. We validated other gene targets as conferring resistance across suppressive conditions, such as *PFN1*, *FAM49B* (also known as *CYRIB*), *CBLB* and *RASA2*. Although published data support the roles of *CBLB*, *FAM49B* and *PFN1* in regulating T cell function, to our knowledge, *RASA2* has not been previously well-defined as a regulator of immune cells^8,13,23^.

**Extended Data Fig. 2:**
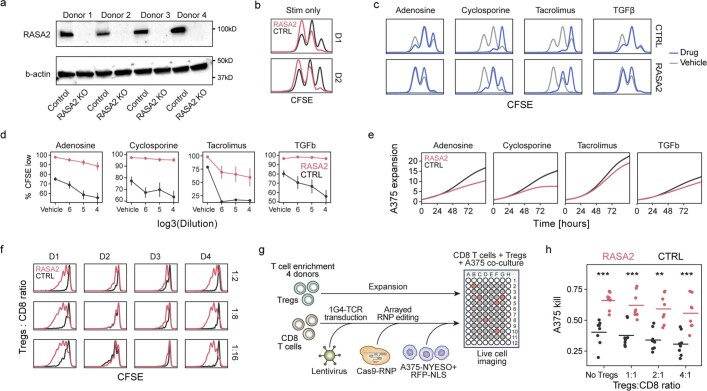
RASA2 validates as a T cell target to engineer resistance to suppressive conditions. **a**, Western blots showing level of RASA2 ablation in T cells from 4 human blood donors. **b**, RASA2 KO T cells have a stimulation dependent proliferative advantage over control T cells, for two independent human donors (D1 and D2). **c**, CFSE staining traces showing how suppressive conditions inhibited T cell proliferation compared to vehicle conditions, for one representative human T cell donor. We note that although both RASA2-KO and control-edited (CTRL) T cells were inhibited by the suppressive signals to varying degrees, RASA2 KO T cells retain a consistent proliferation advantage in each condition. **d**, Summary of gated CFSE low (dividing) cells on y-axis across a range of suppressive conditions on x-axis (mean +/- SEM, n = 2 donors in 3 replicates). **e**, Cancer killing assay by NY-ESO-1-specific 1G4 TCR-T cells in the presence of suppressive molecules. Lines show the count of A375-RFP+ cancer cells as detected by live-cell microscopy, normalized by their count at t = 0. Grey area represents a 95% confidence interval of one representative donor with 3 technical replicates. **f**, Treg suppression assay. Stimulated CD8 T cells were stained by CFSE to track their proliferation in co-culture with suppressive Tregs. T cells from four distinct human donors are shown across the columns, with Tregs:CD8 ratio shown in the rows. **g**, Schematic of cancer killing assay by effector T cells in the presence of Tregs. **h**, Cancer cell killing (calculated by 1 - scaled AUC for the cancer cell growth curve) shown on the y-axis for a range of Tregs:CD8 ratios. Horizontal lines are the mean, dots are individual wells (CTRL = non-targeting guide, n = 4 human donors, each in 2 replicates, **p < 0.01 and ***p < 0.001 for two-sided Wilcoxon test). Source data

We previously identified *RASA2* as a gene target that boosts T cell proliferation and in vitro cancer cell-killing capacity when it is knocked out^13^. Having observed that *RASA2* ablation also promotes T cell proliferative capacity under multiple immunosuppressive environments, we focused our subsequent efforts on characterizing the effects of *RASA2* ablation in preclinical models of adoptive cell therapy. RASA2 is a RasGAP that suppresses RAS signal output by accelerating the hydrolysis of active RAS-GTP to RAS-GDP^24,25^. In these screens, *RASA2* was unique among the RasGAP family in inhibiting T cell proliferation as evidenced by multiple *RASA2*-targeting guides in multiple donors being enriched in the dividing T cells (Fig. 1c,d and Extended Data Fig. 1g). By contrast, guides targeting the gene encoding the RAS guanine nucleotide exchange factor (RasGEF) *RASGRP1* were depleted from dividing T cells, confirming its known role as a positive regulator of TCR and RAS signaling^26^ (Fig. 1d). Analysis of global gene expression patterns across tissues^27^ showed that *RASA2* is expressed selectively in CD8^+^ and CD4^+^ human T cells, a pattern distinct from other RasGAP family members but very similar to that observed for the *RASGRP1*^26^ (Extended Data Fig. 1h). Targeted *RASA2* ablation with individual CRISPR guides in two additional donors reproduced the proliferative advantage observed in the screens under all four soluble-factor suppressive conditions (Fig. 1e and Extended Data Fig. 2a–d).

We further tested whether *RASA2*-deficient T cells exhibit increased in vitro killing of cancer cells under these immunosuppressive conditions. *RASA2* ablation boosted cancer cell killing by TCR T cells compared with control-edited T cells across this range of suppressive conditions (Fig. 1f and Extended Data Fig. 2e). A co-culture suppression assay with T_reg_ cells further confirmed that RASA2 inactivation renders effector T cells resistant to T_reg_ cell-mediated inhibition of proliferation (Fig. 1g and Extended Data Fig. 2f). This resistance to suppression was also evident in cancer cell-killing assays performed in the presence of T_reg_ cells (Fig. 1h and Extended Data Fig. 2g,h). Whereas RASA2-deficient effector T cells maintained their robust cytotoxic function, control T cells were unable to control tumour cell growth in the presence of suppressive T_reg_ cells. These findings support the idea that RASA2 normally serves as a negative regulator of T cell proliferation and cytotoxic function and that *RASA2* ablation confers resistance to multiple mechanisms that suppress the anti-tumour activity of adoptive T cells.

## RASA2 regulates TCR-dependent RAS signalling

**Fig. 2:**
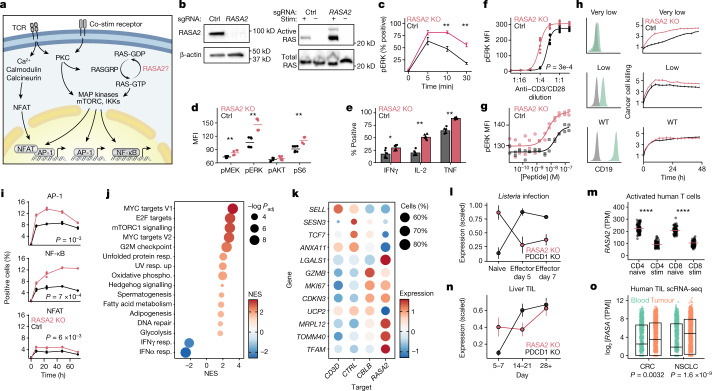
*RASA2* ablation promotes T cell activation, antigen sensitivity and effector function. **a**, RAS signaling and downstream transcriptional programmes in T cells. Drawing is adapted from ref. ^29^. IKK, inhibitor of NF-κB kinase. **b**, Western blot showing RASA2 protein expression in Jurkat cells and GTP-bound active RAS after TCR stimulation. **c**, Flow cytometry-based analysis of phospho-ERK kinetics in stimulated primary human T cells. **d**, Scaled phosphoprotein mean fluorescence intensity (MFI) in MAPK and AKT–mTOR pathways. **e**, Effector cytokine levels in stimulated T cells. **f**,**g**, pERK levels 10 min after TCR stimulation with anti-CD3/CD28 (**f**) or T2 cells preloaded with cognate peptide (**g**). **h**, Left, CD19 expression on engineered Nalm6 cancer target cells (green) compared with unstained cells (grey). Right, CAR T cell killing of Nalm6 cells expressing varying CD19 levels, measured by annexin staining. Data are mean ± s.d. of technical triplicates from one representative donor out of two. WT, wild type. **i**, Percentage of Jurkat cells positive for transcription factor-responsive mCherry reporters. **j**, GSEA of differentially expressed genes between RASA2-KO and control cells after TCR stimulation. Dot size represents adjusted *P*-value (*P*_adj_; two-sided permutation test). NES, normalized enrichment score; phospho, phosphorylation; resp., response. **k**, Differentially expressed genes in stimulated RASA2-KO T cells with perturbation of the indicated target genes^13^. Colour indicates mean expression level and size shows the percentage of cells with detectable expression (*n* = 2 donors). **l**–**o**, RASA2 expression in a mouse model of *Listeria* infection^38^ (**l**; *n* = 3 mice; mean ± s.e.m.), in vitro activated human T cells^20^ (**m**; *n* = 91 donors; two-sided Wilcoxon test), a mouse model of tumour-infiltrating T cells^38^ (TIL) (**n**, showing days after T cell transfer; *n* = 3 mice; mean ± s.e.m.) and human tumour-infiltrating T cells (orange) or peripheral T cells (green) (**o**). **o**, Box limits show quartiles, the horizontal line is the median (*n* = 12 donors for colorectal cancer^40^ (CRC) and *n* = 14 donors for non-small cell lung carcinoma^41^ (NSCLC); two-sided Wilcoxon test). **c**–**e**, Lines show mean; *n* = 2 donors in triplicate; two-sided Wilcoxon test. **f**,**g**, *n* = 2 donors in triplicate; fitted 4-parameter dose–response curves; two-sample Kolmogorov–Smirnov test. *P < 0.05, **P < 0.01, ****P < 0.0001. Source data

**Extended Data Fig. 3:**
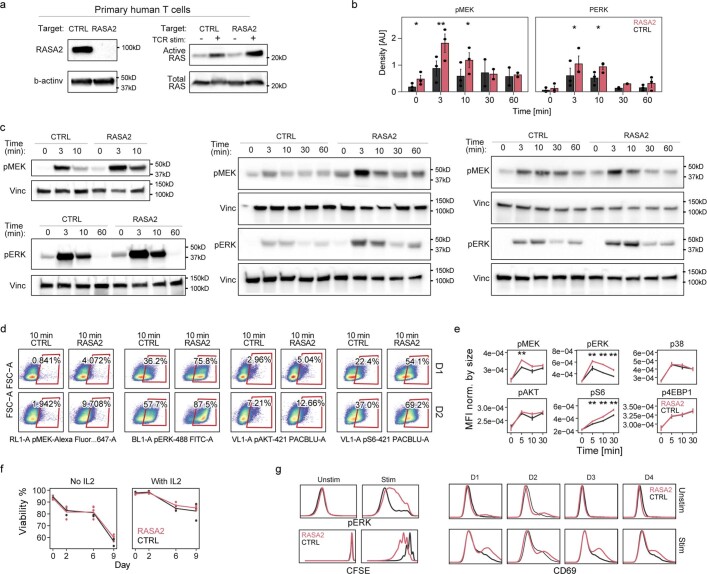
RASA2 is a TCR stimulation-dependent attenuator of Ras-MAPK signaling. **a**, Western blot showing effect of RASA2 ablation on the level of active Ras in primary human T cells with or without TCR stimulation. **b**, Densitometry measurements calculated for p-MEK and p-ERK westerns and averaged for all 3 T cell donors (mean ± SEM, *p < 0.05 and **p < 0.01 for two-sided paired t-test) **c**, Western blots showing the effect of RASA2 ablation compared to control-edited T cells for phospho-MEK and phospho-ERK over time after TCR stimulation in primary human T cells from 3 human donors from (b). **d**, Flow cytometry plots showing representative gating for phospho-proteins in the Ras signaling pathway in 2 human donor T cells after TCR stimulation. **e**, Summary of MFI for phospho-proteins in Ras signaling pathways over time after TCR stimulation. Y-axis shows MFI for each marker divided by MFI for FSC-A to normalize for cell size (mean +/− SEM, n = 2 donors in 3 replicates, **p < 0.01 for two-sided Wilcoxon test). **f**, T cells from 2 donors after 13 days of expansion in culture split into cultures with or without IL2 in triplicates and viability was tracked over time. Lines are mean, individual dots are replicates. **g**, Flow cytometry histogram plots from stimulated and unstimulated T cells for pERK, CD69 and CFSE. Baseline levels of RASA2 KO and control-edited T cells remained similar (except for variability in CD69 levels), while after CD3/CD28 stimulation, RASA2 KO T cells showed higher levels of pERK, CD69 and proliferation over CTRL KO T cells. Results representative of 4 human donors. We noted heterogeneous expression of CD69 at baseline across donors, with some showing marginally higher levels in RASA2 KO T cells. Source data

We next sought to define the effects of inactivating RASA2 on RAS-GTP levels and downstream signaling events in human T cells. RASA2 is predicted to attenuate RAS signalling, a major intersection for multiple pathways in T cells that control cell activation, proliferation and differentiation^28,29^ (Fig. 2a). The Jurkat T cell leukaemia cell line and primary human T cells express normal RAS proteins, and as expected in cells lacking an oncogenic *RAS* mutation^30^, basal RAS-GTP levels were low in both cell types but increased in response to TCR stimulation (Fig. 2b and Extended Data Fig. 3a). We found that knocking out RASA2 resulted in higher RAS-GTP levels in response to TCR stimulation, in agreement with its known function as a GTPase-activating protein for RAS. These biochemical data are consistent with the results of our CRISPR screens, which support a non-redundant role of RASA2 in regulating RAS output in T cells that is not rescued by other GTPase-activating proteins. MEK and ERK are key downstream effectors of RAS-GTP in the MAPK signalling pathway^29^. Consistent with the increased levels of RAS-GTP upon TCR activation, we observed higher levels of MEK and ERK phosphorylation in the RASA2-KO primary T cells compared with corresponding controls. Although RASA2-KO T cells followed similar overall kinetics of MAPK signalling as control cells, they reached a higher peak amplitude of phosphorylated (p)ERK and pMEK levels (Fig. 2c and Extended Data Fig. 3b,c). RASA2-KO T cells also had higher levels of stimulation-induced phosphorylation of S6, a further downstream mediator of the MAPK signalling cascade (Fig. 2d and Extended Data Fig. 3d,e). Together, these data support a role for RASA2 as a RasGAP regulating the MAPK signalling response to TCR stimulation.

**Extended Data Fig. 4:**
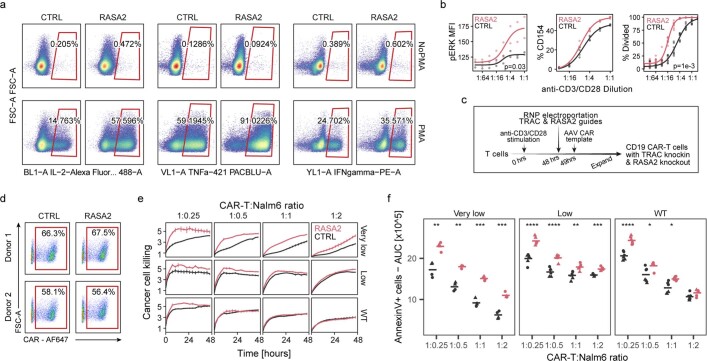
RASA2 ablation increases the sensitivity to antigen stimulation. **a**, Flow cytometry plots showing gating for cytokines in control (CTRL) and RASA2 KO T cells with (bottom row) and without (top row) PMA stimulation. **b, left:** Phosphorylated ERK levels (y-axis) measured by flow cytometry 10 min after TCR stimulation with titrated concentrations of anti-CD3/CD28 complexes (1:1 is 25 µl/ml Immunocult, n = 2 T cell donors in duplicates, lines show the 4-parameter logistic fit); **middle:** Percentage of cells positive for the T cell activation marker CD154 measured 18 h after stimulation with titrated concentrations of anti-CD3/CD28 complexes (1:1 = 25 µl/ml Immunocult, n = 2 T cell donors in duplicate); **right:** percentage of cells that divided based on CFSE profiles 3 days after stimulation with anti-CD3/CD28 complexes (n = 2 T cell donors in triplicates, lines show the 4-parameter logistic fit, p-value from a two-sample Kolmogorov-Smirnov test). **c**, Schema of CD19 CAR-T cell production, where the CAR is knocked into the *TRAC* locus^46^ and RASA2 versus AAS1 is targeted with Cas9 RNPs. **d**, Flow cytometry plots of CD19-CAR positive T cells after the CAR knockin strategy described in (**c**). **e**, *TRAC* CAR-T cell killing of target Nalm6 cells engineered to express varying CD19 levels (rows) measured by annexin levels in live cell microscopy across increasing CAR-T:Nalm6 ratios (columns) over 48 h (one representative donor out of two, 3 technical replicates, error bars are mean ± SD). **f**, Summary of cancer cell killing (scaled AUC of annexin levels) shown on the y-axis for a range of effector T cells to target cell ratios. Horizontal lines are the mean (n = 2 human donors, each in triplicates, *p < 0.05, **p < 0.01, ***p < 0.001, ****p < 0.0001, for two-sided unpaired Student’s t-test, shape denotes donor). Source data

We also confirmed that *RASA2* ablation does not cause unregulated T cell proliferation, which might reduce its utility as a target for gene editing in T cell therapies. In the absence of TCR stimulation, the viability of both control and RASA2-KO T cells steadily declined, and withdrawal of interleukin-2 (IL-2) enhanced this decline (Extended Data Fig. 3f). We found that RASA2-KO T cells remain dependent on TCR stimulation for MAPK signalling (indicated by pERK), proliferation (indicated by CFSE staining) and activation (indicated by CD69 expression), with no consistent change in baseline levels, except for CD69 which showed variable expression^13^ (Extended Data Fig. 3g). Additionally, we detected higher levels of multiple effector cytokines in RASA2-deficient T cells compared with control T cells in response to TCR stimulation, with no differences noted in the unstimulated cells (Fig. 2e and Extended Data Fig. 4a). Together, these results demonstrate that in TCR stimulated T cells, *RASA2* ablation boosts a cascade of key signalling pathways to promote more potent effector functions. Notably, *RASA2* ablation does not cause loss of cytokine dependence or unregulated proliferation in the absence of TCR stimulation.

## *RASA2* ablation sensitizes T cells to antigen

We next tested whether ablating *RASA2* in T cells amplifies sensitivity to lower levels of target cognate antigen in vitro. RASA2-KO T cells had higher levels of ERK phosphorylation, activation and proliferation compared with control T cells across a wide range of concentrations of anti-CD3 and anti-CD28 (anti-CD3/CD28) (Fig. 2f and Extended Data Fig. 4b). To measure this antigen sensitivity with a more physiological stimulus, NY-ESO-1 antigen-specific T cells were co-cultured with T2 cells preloaded with increasing concentrations of the cognate NY-ESO-1 peptide. This assay confirmed that RASA2 KO led to higher levels of pERK across a range of peptide concentrations, effectively sensitizing T cells to antigen (Fig. 2g). Increased antigen sensitivity could be particularly important in engineering T cells that are able to detect and kill cancer cells with low target-antigen expression^31,32^. To test this, we engineered T cells to express a CAR targeting the CD19 surface protein and edited them to disrupt either *RASA2* or a control locus (Extended Data Fig. 4c,d and Methods). We used a CD28-based CD19 CAR, which has been reported to be a highly sensitive CAR, to test whether loss of *RASA2* expression might further boost sensitivity to low-antigen targets with *RASA2* ablation^33^. These CAR T cells were co-cultured with cancer cells engineered to express a range of CD19 levels and cancer cell killing was assayed by annexin staining. Whereas both RASA2-KO and control CAR T cells efficiently killed leukaemia cells expressing high CD19 levels, RASA2 inactivation augmented the in vitro killing of leukaemia target cells versus control T cells in the context of low antigen expression (Fig. 2h and Extended Data Fig. 4e,f). Collectively, these data suggest that T cells lacking RASA2 are sensitized even to low antigen levels, which can enhance their ability to detect and kill antigen-dim cancer cells.

## RASA2 KO promotes reprogramming of T cells

**Extended Data Fig. 5:**
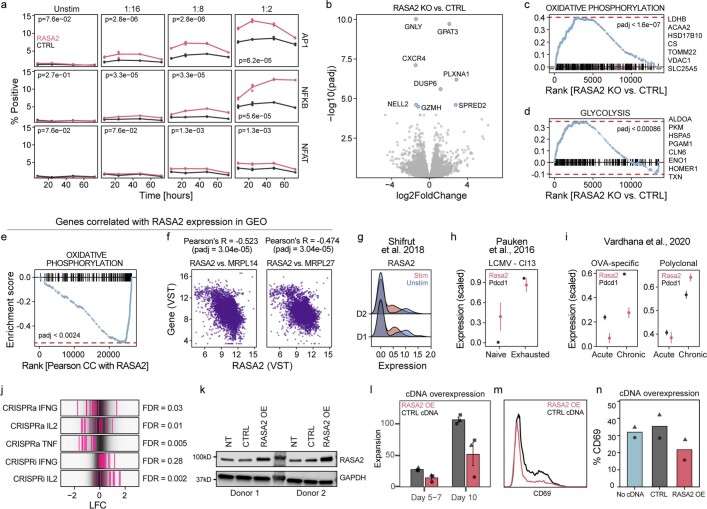
RASA2 knockout promotes transcriptional reprogramming and RASA2 expression is differentially regulated by acute and chronic antigen stimulation. **a**, Fraction of Jurkat cells positive for three different mCherry reporter cell lines responsive to the transcription factor as indicated to the right of each panel, after TCR stimulation with titrated dilutions (1:1 = 25 µl/ml) of anti-CD3/CD28 complexes (columns) over 72 h (dots show 3 technical replicates, p-value from a two-sample Kolmogorov-Smirnov test). **b**, Volcano plot showing differentially regulated genes between RASA2 KO and control-edited stimulated T cells as determined by RNA-Seq. Genes highlighted have BH adjusted, two-tailed Wald test p-value < 0.0001 and absolute log2 fold change > 1, as determined by DESeq2 analysis (methods). **c**, **d** Gene set enrichment analysis (GSEA) of oxidative phosphorylation (c) and glycolysis (d) ranked genes, based on DESeq2, higher rank indicates enrichment in RASA2 KO over CTRL. p-value is shown as determined by a two-sided permutation-test. Top up-regulated genes in each enrichment are listed to the right of each panel **e**, GSEA of oxidative phosphorylation genes correlated with *RASA2* expression in immune cells in the GEO expression database, as retrieved by correlationAnalyzeR (methods). Genes are ranked by the Pearson correlation coefficients between *RASA2* and the query gene, p-value by two-sided permutation test, after FDR adjustment. **f**, Selected examples of expression of *RASA2* and two mitochondrial fitness genes, *MRPL27* (left panel) and *MRPL14* (right panel) across GEO datasets from immune cells. Shown at the top is the Pearson’s correlation coefficient (R) and FDR adjusted p-value (padj) for each scatter plot. Values represent expression after variance stabilizing transformation (VST). **g**, Expression of RASA2 in stimulated T cells compared to unstimulated T cells, as measured by published single-cell RNA-Seq dataset, for two human donors^13^. **h**, **i**, Expression of *Rasa2* compared to *Pdcd1* in published RNA-Seq datasets from models of T cell exhaustion in murine T cells. Expression was scaled for a maximum of 1 and a minimum of 0 for each gene in each dataset (For LCMV samples in (h): n = 2 mice for Naive group, n = 3 mice for exhaustion group; for OVA samples, error bars are mean ± SEM (i): n = 3 mice for all groups, error bars are mean ± SEM). **j**, Log fold change (LFC) values for RASA2 sgRNAs in CRISPRa and CRISPRi screens for cytokine production^42^ (MAGeCK’s gene-level FDR listed for RASA2 in each screen). **k**, Western blot for level of RASA2 expression following *RASA2* transgene or control (CTRL) transduction in two T cell donors. **l**, Normalized values for T cell expansion based on cell counts for T cells with RASA2 transgene versus GFP control (n = 3 human T cell donors, mean ± SEM, shape denotes donor). **m**, histogram for one example donor from cells described in (l), stained and FACS analyzed for CD69 activation marker. **n**, Summary data for CD69 levels in two T cell donors transduced with the RASA2 transgene versus control (n = 2 human T cell donors, shape denotes donor). Source data

We next profiled downstream transcriptional networks in RASA2-KO cells. First, to assess transcriptional programmes key to T cell activation, we used a set of Jurkat T cell transcriptional reporter systems. These reporter lines have been engineered with response elements for activator protein 1 (AP-1), nuclear factor of activated T cells (NFAT) and nuclear factor kappa B (NFкB) driving the expression of an mCherry fluorescent reporter. These studies showed that *RASA2* ablation significantly increased TCR stimulation-induced transcriptional activity of AP-1 and NFкB, and to a lesser extent NFAT, consistent with the established downstream transcriptional effects of RAS and MAPK signalling pathways (Fig. 2i and Extended Data Fig. 5a). To profile transcriptional changes systematically in primary RASA2-KO T cells, we performed whole transcriptome RNA-sequencing (RNA-seq) analysis on either RASA2-KO or control edited antigen-specific T cells after 48 h of co-culture with target cancer cells. Two of the most upregulated genes in RASA2-KO T cells were *DUSP6* and *SPRED2*, which attenuate RAS signalling and are probably upregulated as a feedback mechanism in response to increased RAS signalling^34^ (Extended Data Fig. 5b). Gene set enrichment analysis (GSEA) highlighted multiple key pathways that are upregulated in RASA2-KO T cells, including those associated with cell cycle, transcriptional activity and cell metabolism (Fig. 2j). Notably, given the importance of metabolic state to T cell function, RASA2-deficient T cells showed increased expression of genes involved in oxidative phosphorylation and glycolysis (Extended Data Fig. 5c,d). To test whether these metabolic changes are generally common to hyper-activated T cells, we analysed a single-cell RNA-seq (scRNA-seq) dataset that we generated previously in CRISPR-perturbed primary human T cells^13^. We compared genes that were differentially expressed in RASA2-KO T cells with those in T cells lacking *CBLB*, which encodes a well-characterized negative regulator of TCR signalling. Whereas inactivation of RASA2 or CBLB increased levels of *GZMB*, *MKI67* and *CDKN3* and decreased expression of *SELL* and *TCF7* (Fig. 2k), our analysis revealed that ablation of *RASA2* also induced a unique gene signature. This signature included differential expression of core genes involved in mitochondrial activity, such as *MRPL12, TOMM40, TFAM* and *UCP2*^35,36^. Metabolic regulation by RASA2 was underscored by a strong negative correlation between genes driving oxidative phosphorylation and *RASA2* expression across thousands of transcriptional datasets from immune cells (Extended Data Fig. 5e,f and Methods). Overall, our analysis of the transcriptional state of RASA2 KO T cells revealed a heightened effector memory state (that is, decreased *TCF7* and *SELL* expression) coupled with a higher oxidative phosphorylation state, which is typically associated with central memory T cells^37^.

As—to our knowledge—RASA2 has no previously described roles in T cell biology, we next evaluated its endogenous transcriptional regulation in T cells. Analysis of our previously published scRNA-seq dataset^13^ revealed that *RASA2* is downregulated following stimulation in human T cells (Extended Data Fig. 5g). Further analysis of two published RNA-seq datasets of acute bacterial infection in mice^38^ and a large cohort of in vitro activated human T cells^20^ confirmed that T cell stimulation acutely downregulates *RASA2* expression levels (Fig. 2l,m). This acute endogenous reduction of RASA2 after stimulation may give T cells a window of heightened effector function, and genetic ablation of *RASA2* may amplify this phenomenon through complete and enduring loss of RASA2. Additionally, we tested whether RASA2 may have a role in T cell dysfunction through analysis of external datasets. Consistent with a checkpoint role in regulating T cell function, *RASA2* was upregulated in mouse T cells exposed to chronic infection^39^ or to repeated antigen stimulation^12^, as well as in tumour-infiltrating T cells^38^ (Fig. 2n and Extended Data Fig. 5h,i). Published scRNA-seq datasets from human patients^40,41^ also revealed higher *RASA2* levels in tumour-infiltrating T cells compared with peripheral T cells, suggesting a potential role for RASA2 in dampening T cell responsiveness in the TME (Fig. 2o). This role as a negative regulator was further supported by analysis of a published dataset of genome-wide CRISPR inhibition and CRISPR activation in T cells for cytokine production^42^, which showed that repression of *RASA2* tended to increase production of effector cytokines, and *RASA2* activation tended to reduce production of these cytokines (Extended Data Fig. 5j). Last, we found that transgenic overexpression of RASA2 in human T cells inhibited T cell activation and ex vivo expansion (Extended Data Fig. 5k–n). Together, these observations suggest that RASA2, which is downregulated during acute stimulation, can be upregulated in chronically stimulated T cells to serve as an intrinsic signalling checkpoint to curb T cell function.

## RASA2 KO increases cancer cell-killing capacity

**Fig. 3:**
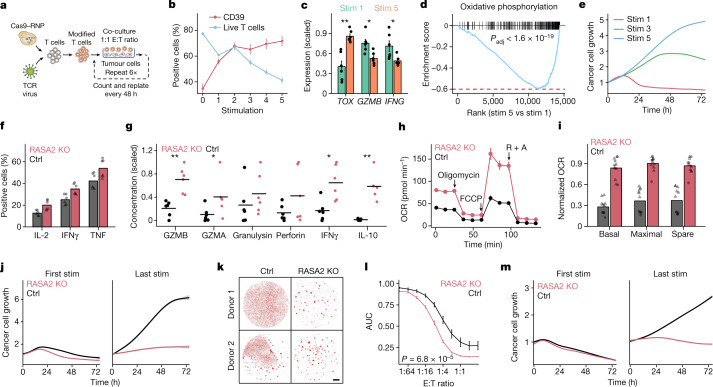
RASA2 ablation improves functional T cell persistence through repeated cancer cell exposures. **a**, Schematic of experiment for modelling T cell persistence in vitro. RNP, ribonuclear protein. **b**, T cell viability and CD39 levels were measured by flow cytometry after each stimulation (*n* = 4 donors; mean ± s.e.m.). **c**, Expression of key genes in T cells by RNA-seq after the first and fifth stimulations (*n* = 3 donors, stimulated via CAR or TCR; mean ± s.e.m.; two-sided Wilcoxon test). **d**, GSEA of differentially expressed genes between T cells after first and fifth stimulation. Adjusted *P*-value by two-sided permutation test. **e**, Cancer cell growth in co-culture with TCR T cells after multiple stimulations. The line is the fitted mean for triplicates. **f**,**g**, Effector cytokine production after repeated stimulations, as measured by flow cytometry (**f**; *n* = 2 donors in triplicate; shape denotes donor) or by multiplex ELISA (**g**; *n* = 3 donors; technical duplicates as dots; lines show mean; two-sided Wilcoxon test). **h**, Oxygen consumption rate (OCR) trace of TCR T cells after repeated tumour stimulations. Arrows mark addition of oligomycin, FCCP and rotenone + antimycin A (R + A) (one donor in 6 technical replicates; mean ± s.d.) **i**, Oxygen consumption rate measured in mitochondrial stress test (*n* = 2 donors in 6 technical replicates; shape denotes donor; values normalized to a maximum of 1 for each donor). **j**, Cancer cell killing after 1 and 5 stimulations. The shaded area shows the 95% confidence interval for triplicates. **k**, Imaging of RFP^+^ A375 cells co-cultured with T cells exposed to repeated stimulations. Scale bar, 1 mm. **l**, Summary statistics for area under the growth curve of cancer cells over a range of effector T cell:target cell ratios (*n* = 7 donors; mean ± s.e.m.; two-sample Kolmogorov–Smirnov test). **m**, RASA2-KO CD19 CAR T cells maintained efficient killing after six previous stimulations. Data are representative of one of three donors. The shaded area shows the 95% confidence interval for triplicates. Statistical tests as indicated, *P < 0.05, **P < 0.01. Source data

**Extended Data Fig. 6:**
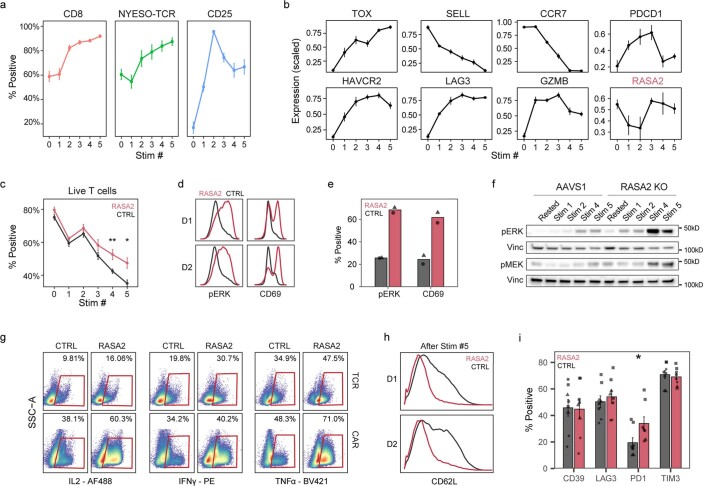
Repetitive tumor stimulation assay shows that RASA2 ablation rescues T cells from a dysfunctional state. **a**, Metrics of NY-ESO-1-specific 1G4 TCR-T cells after each repetitive co-culture with A375 tumor cells, including percent positive for CD8, NY-ESO-1 1G4 TCR, and activation marker CD25 (assessed by flow cytometry, n = 4 T cell donors, lines are mean ± SEM). **b**, Gene expression levels for selected genes, including *RASA2*, with repeated tumor stimulation measured by RNAseq (n = 3 donors TCR-T cells and n = 3 donors CAR-T cells, mean ± SEM). **c**, T cell viability, measured by flow cytometry with Live/Dead stain, compared between RASA2 KO and control (CTRL) T cells (n = 4 donors, mean ± SEM, *p < 0.05 and **p < 0.01 for two-sided Wilcoxon test). **d**, RASA2 KO T cells following multiple stimulations show higher levels of phosphorylated ERK and CD69 compared to control cells (n = 2 donors). **e**, Fraction of T cells positive by flow cytometry for p-ERK and CD69 after 6 repeated co-cultures with A375 tumor cells (n = 2 donors, mean ± SEM, *p < 0.05 for two-sided Wilcoxon test). **f**, Western blot analysis for p-ERK and p-MEK levels in T cells after each repeated CD3/CD28 stimulation. **g**, Flow cytometry data for multiple effector cytokines (labeled on bottom) in NY-ESO-1-specific TCR-T cells (top row) and CD19-specific CAR-T cells (bottom row) after 6 repeated co-cultures with target tumor cells. **h**, Histograms showing CD62L levels in NY-ESO-1-specific T cells in 2 donors after 6 repeated co-cultures with A375 tumor cells. **i**, Percent of cells expression exhaustion-associated markers as measured by flow cytometry of T cells after multiple stimulations show similar levels between RASA2 KO and control-edited (CTRL) T cells (n = 4 donors, mean ± SEM, *p < 0.05 and ns is p > 0.05 for two-sided Wilcoxon test, shape denotes donor). Source data

We next tested whether ablation of *RASA2*, which we found to be upregulated in tumor-infiltrating T cells, would ameliorate chronic antigen-exposure-induced T cell dysfunction. We established a repetitive stimulation assay where antigen-specific T cells are co-cultured with fresh target tumour cells at 1:1 effector to target (E:T) ratios repeatedly every 48 h (Fig. 3a and Methods). This repetitive stimulation assay showed a relative enrichment in antigen-specific T cells, a decline in T cell viability and activation levels, a change in metabolic profile, and progressive changes in key cell phenotyping markers, collectively consistent with a dysfunctional T cell state^43^ (Fig. 3b–d and Extended Data Fig. 6a,b). At a functional level, T cells gradually lost the ability to control the expansion of cancer cells after repeated exposures (Fig. 3e).

**Extended Data Fig. 7:**
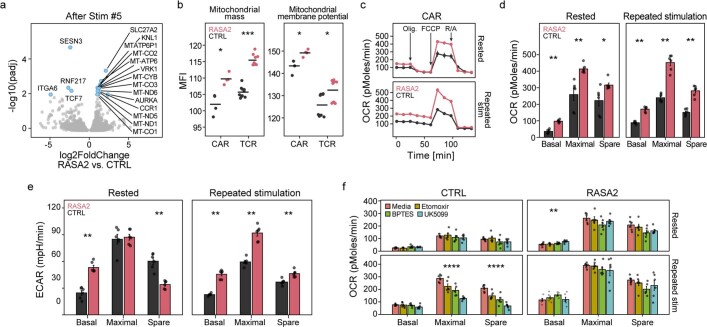
Metabolic fitness of RASA2 KO T cells. **a**, Volcano plot (log2 fold change on the x-axis, -log10 of unadjusted p-value from a two-tailed Wald test on the y-axis) of RNA-Seq analysis following five stimulations compared between RASA2 KO or control-edited T cells (n = 3 independent donors). We note TCF7 is lower in RASA2 KO compared to control-edited T cells and that multiple mitochondrial fitness genes are up-regulated following RASA2 deletion. **b**, MFI as measured by flow cytometry for mitochondrial mass (Mitotracker green) and for mitochondrial membrane potential (MitoTracker Red CMXRos) in NY-ESO-1 TCR-T and CD19 CAR-T cells after repetitive cancer target stimulations. (n = 2 human donors, each in 2 technical replicates for CAR-T cells and in 5 technical replicates for TCR-T cells, *p < 0.05 and ***p < 0.001 for two-sided Wilcoxon test). **c**, Oxygen consumption rate (OCR) traces as measured by seahorse mitochondrial stress test for CD19 TRAC-CAR T cells that were not exposed to cancer cells (rested) or were exposed to six repeated tumor stimulations. Arrows mark addition of each inhibitor: oligomycin 1.5 μM (Olig.), FCCP 1μM, rotenone/antimycin A 0.5 μM (R/A) (n = 6 technical replicates for one representative T cell donor of two, lines are mean ± SD). **d**, Average basal OCR, maximal OCR, and spare respiratory capacity (SRC) levels in seahorse mito stress test (n = 6 technical replicates for one representative T cell donor of two, lines are mean ± SD, *p < 0.05 and **p < 0.01 for two-sided Wilcoxon test). **e**, Same experiment as (d) but here showing extracellular acidification rates (ECAR). SGC = spare glycolytic capacity (n = 6 technical replicates for one representative T cell donor of two, lines are mean ± SD, **p < 0.01 for two-sided Wilcoxon test. **f**, OCR measurements from Seahorse substrate oxidation stress tests performed in CD19 CAR-T cells before co-culture with cancer cells or after six repeated stimulations by cancer cell targets (n = 1 human donor, with 6 technical replicates per condition, **p < 0.01, ****p < 0.0001 for one-way ANOVA test). Substrate inhibitors included 4 μM Etomoxir (XF Long Chain Fatty Acids), 2 μM BPTES (Glutamine), and 3 μM UK5099 (Glucose/Pyruvate). Source data

RNA-seq analysis of *RASA2* expression in repetitively stimulated T cells showed that although *RASA2* levels declined after acute stimulation, they increased upon repeated tumour exposures (Extended Data Fig. 6b). These findings further suggested that RASA2 can act as a checkpoint to restrain T cell responses in the setting of chronic stimulations. We tested this at a functional level in the repetitive stimulation assay and found that *RASA2* ablation generally limited many of the dysfunctional phenotypes. For instance, *RASA2* ablation limited the observed decline in T cell viability seen with repeated tumour exposures (Extended Data Fig. 6c). We also observed that RASA2-KO T cells demonstrated higher levels of phospho-MAPK signalling, activation and multiple effector cytokines compared with control-edited T cells after repeated stimulations (Fig. 3f and Extended Data Fig. 6d–g). An enhanced effector state of RASA2-deficient T cells was confirmed independently using an ELISA assay to measure immunomodulatory cytokines and cytolytic molecules in the supernatant of stimulated T cells (Fig. 3g). RASA2*-*KO T cells were found to be in a more effector-memory-differentiated state than control cells (Extended Data Fig. 6h). Canonical T cell exhaustion genes were similar between *RASA2* and control-edited T cells after multiple stimulations, suggesting that RASA2-KO T cells were not differentially exhausted in vitro (Extended Data Fig. 6i). RNA-seq analysis showed that RASA2-KO T cells expressed higher levels of genes associated with the cell cycle (*VRK1*, *AURKA* and *KNL1*), fatty acid metabolism (*SLC27A2*) and mitochondria compared with control-edited T cells after repeated stimulations (Extended Data Fig. 7a). Given the importance of metabolic fitness in resisting T cell dysfunction, we assessed metabolic profiles on a functional level in control and RASA2-KO T cells in this repeated stimulation experiment^44^. A flow-cytometry-based assay confirmed higher mitochondrial mass and activity in both CAR T and TCR T cells lacking RASA2 relative to control cells (Extended Data Fig. 7b). Seahorse real-time cell metabolic analysis showed that *RASA2* ablation led to increased basal and maximal oxygen consumption rates and extracellular acidification rates compared with control-edited T cells after repeated stimulation (Fig. 3h,i and Extended Data Fig. 7c–e). Whereas control T cells could not use alternative energy sources after chronic stimulation, RASA2-KO T cells maintained this ability despite the repeated antigen exposures (Extended Data Fig. 7f). In summary, RASA2 ablation limits dysfunction from chronic cancer antigen exposure across an array of diverse phenotypic metrics.

**Extended Data Fig. 8:**
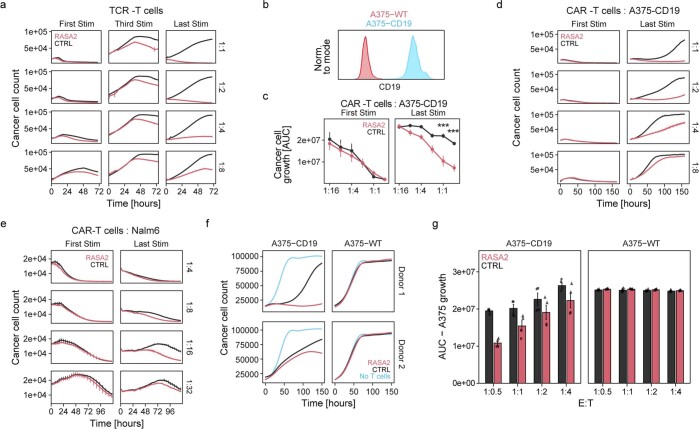
Ablation of RASA2 preserves cancer cell killing capacity in T cells after repeated target cancer cell exposures. **a**, Cancer cell killing assay showed control-edited NY-ESO-1-specific TCR-T cells failed to control cancer cell expansion after multiple stimulations, whereas RASA2 ablation led to persistent killing capability. Cancer cell kill after 1, 3, and 6 repeated co-cultures (columns) and for a range of effector T cells to target cancer cell ratios (rows) are displayed over time (each time point in triplicates, lines are mean ± SD, lines show a fitted curve by a generalized additive model, see methods). **b**, Representative flow cytometry histograms showing CD19 staining in A375 cells engineered to express CD19 (A375-CD19 in cyan) compared to unperturbed A375 cells (A375-WT in red). **c**, Summary statistics for area under the growth curve over target A375-CD19 cells a range of effector T cells to target cell ratios after 1 and 6 repeated cancer cell exposures (n = 3 donors in duplicates, mean ± SEM, ***p < 0.001 for two-sided Wilcoxon test). **d**, Representative data for cancer killing assays in (c) by CD19-specific CAR-T cells after 1 and 6 repeated co-cultures for a range of effector T cells to target cancer cell ratios (rows) with target A375-CD19 melanoma cells expressing CD19. **e**, Representative data from one CAR-T cell donor for cancer killing assays with target Nalm6 leukemia cells (each time point in triplicates, lines are mean ± SD). **f**, Traces of cancer cell count over time as detected by live-cell microscopy. CD19 CAR-T cells from two donors after repetitive stimulation were co-cultured with either unperturbed target cells (A375-WT) or an isogenic cell line engineered to express CD19 (A375-CD19). No cancer cell killing is observed with either RASA2 KO or CTRL T cells in the antigen-negative condition. Lines are mean of 3 technical replicates. **g**, Quantification of data in (f), for the area under the growth curve (y-axis), across multiple effector T cell to target cancer cell ratios (x-axis) (n = 2 donors in triplicates, mean ± SEM, shape denotes donor). Source data

Next, we tested whether the cancer cell-killing capacity of *RASA2*-ablated T cells is affected by repeated exposure to tumour antigen. Although T cells with *RASA2* ablation had a moderate advantage in our cancer cell-killing assay upon first stimulation, this advantage became even more marked after multiple stimulations (Fig. 3j,k). In contrast to control-edited T cells that showed a gradual decline in the ability to control the growth of cancer cells with each stimulation, *RASA2*-ablated T cells maintained their robust killing capacity after multiple stimulations (Extended Data Fig. 8a). This cancer cell-killing advantage was generally consistent across multiple human blood donors and ratios of effector T cell to cancer cells (Fig. 3l). We next tested whether this resistance to T cell dysfunction with *RASA2* loss was replicated in TRAC CAR T cells. *RASA2*-edited TRAC CD19-specific CAR T cells were co-cultured repeatedly with CD19-expressing cancer cells (Extended Data Fig. 8b). As seen with the TCR T cell model, *RASA2*-edited CAR T cells continued to kill target cells efficiently following repeated cancer cell exposures, whereas the control-edited CAR T cells were unable to control tumour cell growth (Fig. 3m). This persistent killing was consistent using two different CD19^+^ cancer cell lines and multiple human blood donors (Extended Data Fig. 8c–e). This killing advantage after repetitive stimulation was specific, as demonstrated by the lack of cancer cell killing when either RASA2-KO or control TRAC CAR T cells were co-cultured with antigen-negative cancer cells (Extended Data Fig. 8f,g). Collectively, these results show that T cells repeatedly exposed to their target antigen gradually lose the ability to control cancer cell growth, whereas ablation of *RASA2* can render both TCR T and CAR T cells resistant to this dysfunctional state.

## RASA2 KO improves T cell anti-tumour responses

**Fig. 4:**
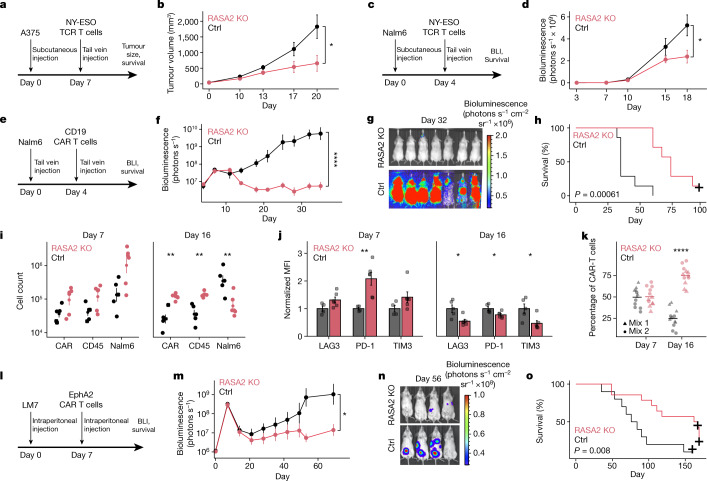
RASA2 ablation improves in vivo tumour control by engineered T cells in multiple preclinical models. **a**,**b**, NY-ESO-1^+^ A375 melanoma cells were engrafted into NSG mice via flank injection and NY-ESO-1-specific TCR T cells were injected via the tail vein. **a**, Experimental timeline. **b**, Tumour growth was monitored with calliper measurements (*n* = 6 mice per group; mean ± s.e.m.; two-sided unpaired Student’s *t*-test). **c**,**d**, NY-ESO-1^+^ Nalm6 leukaemia cells were injected into NSG mice followed by NY-ESO-1-specific TCR T cells. **c**, Experimental timeline. BLI, bioluminescence live imaging. **d**, Tumour growth was monitored using luciferase-based bioluminescence live imaging (*n* = 5 mice for RASA2-KO T cells, *n* = 4 for control T cells; mean ± s.e.m.; two-sided unpaired Student’s *t*-test). **e**,**f**, Nalm6 cells were injected into NSG mice followed by CD19-specific CAR T cells. **e**, Experimental timeline. **f**, Tumour growth was monitored by bioluminescence imaging (*n* = 7 mice per group; mean ± s.e.m.; two-sided unpaired Student’s *t*-test). **g**, Bioluminescence imaging of the cohort in **f**, dorsal view. **h**, Survival of the cohort shown in **f**. **i**, Cell counts by flow cytometry in bone marrow of Nalm6-engrafted NSG mice (day 7: *n* = 5 for control, *n* = 6 for RASA2 KO; day 16: *n* = 6 per group; mean ± s.e.m.; two-sided Wilcoxon test). **j**, Mean fluorescence intensity (normalized to control) of inhibitory markers on cells from cohort in **i** (mean ± s.e.m.; two-sided Wilcoxon test). **k**, Percentage of mixed CAR T cell population (originally injected into mice, mixed 50:50 (control:RASA2-KO CAR T cells)), isolated from bone marrow days 7 and 16 after infusion into Nalm6-bearing mice (*n* = 6 mice per group; two-sided Wilcoxon test). **l**–**o**, NSG mice were injected intraperitoneally with LM7-ffLuc tumour cells on day 0, then received a single intraperitoneal injection of control or RASA2-KO EphA2-CAR T-cells. **l**, Experimental timeline. **m**, Quantitative bioluminescence imaging (mean ± s.e.m.; *n* = 10 for control, *n* = 14 for RASA2 KO; two-sided paired Student’s *t*-test). **n**, Representative bioluminescence for each group. **o**, Survival curve for the cohort in **m**. Survival *P*-values by log-rank test. Statistical tests as indicated. *P < 0.05, **P < 0.01, ****P < 0.0001. Source data

**Extended Data Fig. 9:**
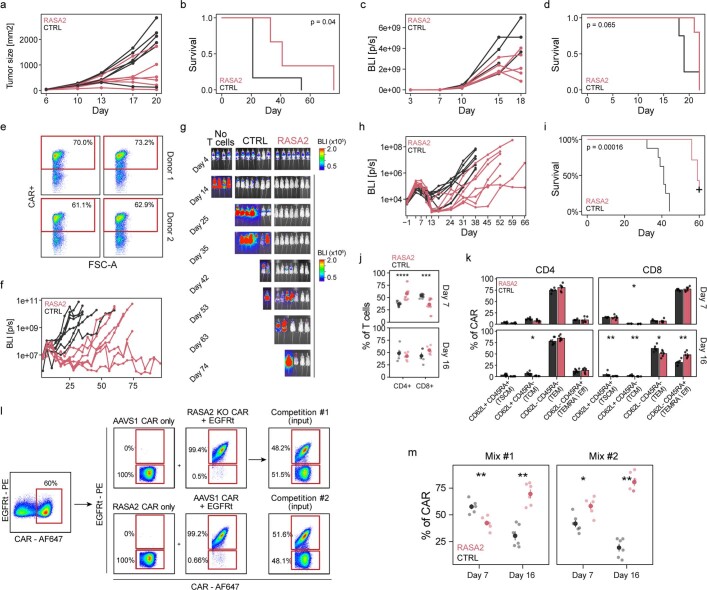
RASA2 ablation in TCR-T and CAR-T cells improves tumor control *in vivo*. **a**, Individual subcutaneous tumor growth by caliper measurements over time in NSG mice engrafted with 1x10^6^ A375 melanoma cells and intravenously injected with 1x10^6^ NY-ESO-1-specific 1G4 TCR-T cells (n = 6 mice per group). **b**, Survival of mice shown in (a), exact p-value by log-rank test. **c**, Individual tumor growth by bioluminescence (BLI as total flux) measurements over time in NSG mice engrafted with 0.3x10^6^ Nalm6 leukemia cells (engineered to express NY-ESO-1), and injected with 0.5x10^6^ NY-ESO-1-specific TCR-T cells (n = 5 mice for RASA2 group, n = 4 for CTRL group). **d**, Survival of mice from two cohorts shown in (c), exact p-value by log-rank test. **e**, Flow cytometry data showing levels of CD19-CAR positivity in T cells immediately prior to injection into Nalm6 bearing mice. Percentages were used to adjust with the goal of equal numbers of CAR+ T cells per mouse. **f**, Individual tumor growth over time in NSG mice engrafted with 0.5x10^6^ Nalm6 leukemia cells and injected with 0.2x10^6^ CD19-specific CAR-T cells (n = 7 mice per group). **g**, Tumor progression in (f) was monitored using bioluminescent imaging (BLI). **h**, Individual tumor growth over time in a cohort of NSG mice bearing Nalm6 leukemia cells injected with CD19-specific CAR-T from an independent human blood donor (n = 8 mice per group for control-edited CAR-T cells and n = 7 mice per group for RASA2 edited CAR-T cells). **i**, Survival of mice shown in (h), exact p-value by log-rank test. **j**, CD4^+^ and CD8^+^ percentage composition of CAR-T cells isolated from the bone marrow of Nalm6-bearing mice day 7 and 16 after CAR-T cell infusion (Day 7: n = 5 for CTRL, n = 6 for RASA2, Day 16: n = 6 for CTRL, n = 6 for RASA2, error bars mean ± SEM, *p < 0.05, **p < 0.01, ***p < 0.001 and ****p < 0.0001 for two-sided Wilcoxon test). **k**, Differentiation status based on CD45RA and CD62L staining for CAR-T cells from cohort in (j) (n = 6 per group, mean ± SEM, *p < 0.05, **p < 0.01 for two-sided Wilcoxon test, TSCM = CD62L+CD45RA+, TCM = CD62L+CD45RA-, TEM = CD62L-CD45RA-, TEMRA/EFF = CD62L-CD45RA+). **l**, Gating for input mixed cell populations for *in vivo* competition assay. T cells are first gated on CAR^+^ cells, then EGFR+/- populations. Input mix 1 and mix 2 each had ~50/50% AAVS1 and RASA2 KO CAR-T cells, with the EGFRt on opposite populations in each mix for identification. **m**, Percentage of the two different mixed CAR-T cell populations after isolation from bone marrow at days 7 and 16 post infusion into Nalm6-bearing mice and stained for CAR and EGFRt markers to determine CTRL/RASA2 KO CAR-T cells percentages *in vivo* (Day 7: n = 6 for CTRL, n = 6 for RASA2, Day 16: n = 6 for CTRL, n = 6 for RASA2, error bars are mean ± SEM, *p < 0.05 and **p < 0.01 for two-sided Wilcoxon test). Source data

To determine the translational relevance of these findings, we proceeded to test whether ablation of RASA2 would improve the performance of engineered T cells in multiple preclinical models of adoptive T cell therapies. First, we engrafted A375 melanoma cells, which express NY-ESO-1, in the flanks of immunodeficient NSG mice (Fig. 4a). T cells engineered to express the 1G4 NY-ESO-1-specific TCR^45^ and edited to ablate *RASA2* or a safe-harbour control locus (*AAVS1*) were transferred via tail vein injection. Transfer of *RASA2*-deficient T cells significantly slowed tumour growth and improved survival compared with mice that received control-edited T cells (Fig. 4b and Extended Data Fig. 9a,b). To test whether RASA2 ablation in TCR T cells could improve control of a liquid tumour bearing the same NY-ESO-1 antigen, we injected Nalm6 leukaemia cells engineered to express NY-ESO-1 on cognate major histocompatibility complex class I molecules (MHCI) into the tail vein of mice (Fig. 4c). In this leukaemia model, *RASA2*-deficient TCR T cells also improved tumour control (Fig. 4d and Extended Data Fig. 9c,d). Thus, *RASA2* ablation enhanced the efficacy of TCR-engineered adoptive T cell therapies in both liquid and solid tumour models.

To test if this advantage of RASA2-KO in vivo is applicable to the CAR T cell context, we generated CD19-specific CAR T cells via knock-in of the CD19-28z CAR into the *TRAC* locus as previously described^46^, with the addition of concurrent disruption of either *RASA2* or of the *AAVS1* locus. These CAR T cells were transferred intravenously into NSG mice engrafted with Nalm6 leukaemia cells (Fig. 4e and Extended Data Fig. 9e). CAR knock-in at the *TRAC* locus has been shown to reduce T cell dysfunction and increase persistence compared with CAR expressed by retroviral vectors^46^. Nonetheless, we found that the *RASA2*-deficient TRAC CAR T cells had a marked advantage over control TRAC CAR T cells in tumour control, as measured by bioluminescence imaging in cohorts of mice treated with cells from multiple different human blood donors (Fig 4f,g and Extended Data Fig. 9f–h). This reduced tumour burden resulted in significantly prolonged survival of the mice that received *RASA2*-deficient TRAC CAR T cells (Fig. 4h and Extended Data Fig. 9i). Whereas all mice injected with the control-edited CAR T cells had to be euthanized by day 60, the majority receiving RASA2-KO human T cells survived past day 60, with a subset demonstrating durable responses beyond 100 days.

**Extended Data Fig. 10:**
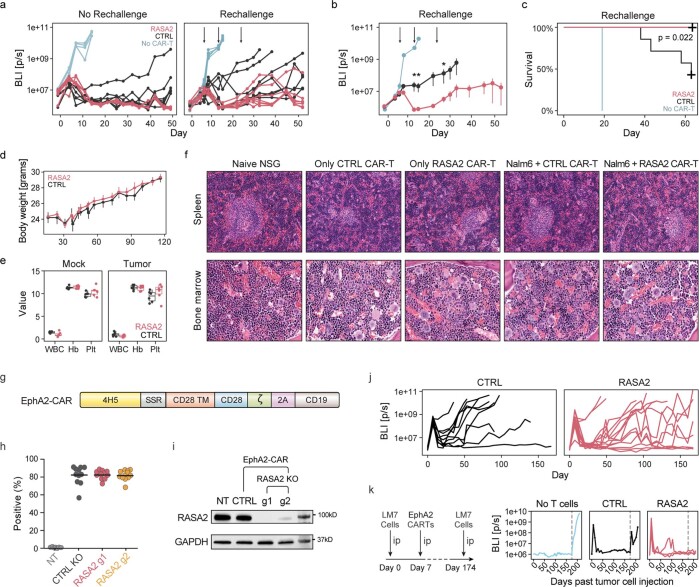
RASA2 ablation boosts CAR-T cell resistance to tumor rechallenge and has a similar safety profile to control CAR-T cells. **a**, BLI values for Nalm6-bearing mice which received 0.2x10^6^ TRAC CAR-T cells after first Nalm6 injection without any rechallenge injections, and for Nalm6-bearing mice which received 0.2x10^6^ CAR-T cells and then 3 further Nalm6 rechallenge injections (1x10^6^ Nalm6/injection). Mice were monitored for tumor burden (BLI levels) and survival. n = 7 mice per arm in each cohort. Arrows depict Nalm6 leukemia rechallenges. **b**, mean ± SEM for BLI values shown in (a) until first mouse death in the control cohort, *p < 0.05 and **p < 0.01 for two-sided unpaired Student’s t-test. **c**, Survival analysis for the leukemia rechallenge model of the cohort shown in (a,b). Exact p-value by log-rank test. **d**, Body weights over time for mice receiving CAR-T cells with no tumor cells engrafted (mean ± SEM, n = 2 human donors and 3 mice per group). **e**, White blood cell (WBC; K/μL), hemoglobin (Hb; g/dL), and platelet (Plt; x10^4^ K/μL) counts for mice receiving only CAR-T cells (n = 2 human donors, n = 3 mice per group), as well as mice receiving tumor-clearing CAR-T cell infusions (n = 1 human donor, n = 6 mice per group, box shows the upper and lower quartiles, horizontal line is median). **f**, Representative H&Es from bone marrow and spleens of mice from (e). Sternal bone marrows (40X magnification) showed tri-lineage hematopoiesis, which was similar in all animals. As expected, tumor naive NSG mice had few cells of lymphoid appearance in their white pulp (seen here as few mononuclear cells surrounding a blood vessel) (spleens at 20X magnification). The red pulp of NSG mice contains abundant erythroid precursors (seen here as numerous cells with dense dark nuclei) as well as megakaryocytes. In comparison to the cancer naive NSG mice, the red pulp and white pulp of recipient animals were largely similar; in some mice, independent of group, some expansion of splenic white pulp was seen, consistent with engrafted lymphoid cells. Of importance, recipients of RASA2 KO TRAC CAR-T cells did not show evidence of increased lymphoid infiltrates in comparison to matched recipients of TRAC CAR-T control cells. **g**, Scheme of EphA2-specific CAR retroviral vectors. 4H5: scFV recognizing EphA2. SSR: short spacer region. **h.** Summary data for CAR expression in gene targeted T-cells as measured by flow cytometry (n = 10 for CTRL, n = 14 for RASA2). **i**, Western Blot analysis of RASA2 expression RASA2 KO EphA2-CAR T cells compared to T cells treated with non-targeting guide (CTRL). Two guide RNAs targeting RASA2 were tested. **j**, Individual BLI traces from main Fig. 4m, using both RASA2 sgRNAs for one donor, and sgRNA1 for the second donor. **k**, Mice without detectable BLI from experiment in (j) were re-challenged with a second intraperitoneal (i.p.) tumor injection with 1x10^6^ LM7-ffLuc tumor cells on Day 174. Graphs show quantitative bioluminescence imaging (total flux). Dotted vertical line indicates the second tumor injection. Source data

To better understand this observed tumour-control advantage, we evaluated the bone marrow in a separate cohort of Nalm6 leukaemia-engrafted mice at two time points after CD19 CAR T cell treatment. We found significantly higher numbers of CAR T cells and lower numbers of Nalm6 cells in the mice treated with RASA2-KO CAR T cells than in those treated with control CAR T cells (Fig 4i). In this in vivo model, the RASA2-KO CAR T cells in the bone marrow also showed lower surface expression of canonical exhaustion-associated inhibitory receptors than control CAR T cells (Fig. 4j). Further phenotyping of these cells showed no major differences in CD4^+^:CD8^+^ composition or differentiation status by day 16, with RASA2-KO cells skewed slightly towards less naive states (Extended Data Fig. 9j,k). To directly compare the relative T cell expansion and persistence in the same bone marrow niche, we transferred a mix of roughly equal proportions of RASA2-KO and control T cells to Nalm-6 bearing mice and found that RASA2-KO CAR T cells clearly outcompeted control CAR T cells over time in the bone marrow niche (Fig. 4k and Extended Data Fig. 9l,m). The persistence advantage we observed in the bone marrow as well as in the repeated cancer cell killing assays in vitro led us to test whether RASA2 KO confers an advantage to CAR T cells in controlling repeated leukemia injections in vivo. These experiments required optimization such that CAR T cell doses for a given donor were not too low when mice were already relapsing, and not too high so that all mice strongly controlled tumour rechallenge. We identified a T cell donor that demonstrated relatively durable control of the initial tumour burden at previously identified low ‘stress-test’ CAR T cell doses^46^, and then we re-introduced Nalm6 cells 3 times, 7–11 days apart, in a separate mouse cohort (Extended Data Fig. 10a). We found that RASA2-KO CAR T cells had an advantage over control CAR T cells in reducing tumour burden and increasing survival in this tumour-rechallenge model, demonstrating that *RASA2* ablation can improve functional persistence in vivo (Extended Data Fig. 10b,c).

To assess the effects of adoptive T cell transfer alone on the health of the mice, we injected non-tumour bearing mice with T cells and monitored them over time. In addition, to assess tumour-antigen-stimulated T cells, we treated an additional cohort of mice bearing Nalm6 leukaemia with control and RASA2-KO CD19 TRAC CAR T cells to achieve tumour clearance and observed these mice to 116 days after CAR T cell injections. In both of these cohorts, there were no observed differences in mice receiving the RASA2-KO and control TRAC CAR T cells by visual inspection and body weight, and RASA2 KO did not alter the blood counts or histopathologic findings of recipient animals in comparison to control TRAC CAR T cells (Extended Data Fig. 10e,f). Overall, these data demonstrate that RASA2 can be ablated in CAR T cells to improve anti-tumour efficacy and survival with no apparent increased safety risk in this preclinical model using TRAC CAR T cells.

Finally, given the major clinical challenges in developing CAR T cell therapies for solid tumours, we tested whether RASA2 KO could also enhance CAR T cell function in a preclinical model of solid tumours. We made use of our previously described intraperitoneal locoregional osteosarcoma (LM7) model^47^ and T cells expressing EphA2.CD28z CAR^48^ (Extended Data Fig. 10g). We injected the LM7 osteosarcoma cell line into the peritoneum of NSG mice, followed by injection of T cells engineered to express an EphA2-specific CAR (Fig. 4l). Bioluminescence measurements of tumour burden revealed that ablation of *RASA2* in CAR T cells could significantly slow tumour growth and prolong survival compared with control CAR T cells in this model (Fig 4m–o and Extended Data Fig. 10h–j). In this cohort of mice, in the subset that cleared their tumours, RASA2-KO CAR T cells were able to clear a tumour rechallenge at day 174 (Extended Data Fig. 10k). In summary, we found that *RASA2* ablation can improve the performance of TCR T and CAR T cells against a range of preclinical models of both liquid and solid tumours, highlighting its promising translational potential for multiple immunotherapy indications.

## Discussion

Inhibitory extrinsic and intrinsic cues present major challenges for current adoptive T cell therapies^14^. Large-scale CRISPR genetic screens offer a powerful discovery platform to reveal genetic perturbations that render T cells resistant to these inhibitory signals^3,8–10^. Here we used such a screening platform to model a variety of tumour-relevant suppressive conditions and found that these screens converged on *RASA2* as a promising candidate target for engineering resistance to multiple inhibitory signals. Our results suggest that in the absence of *RASA2*, T cells experience increased RAS signalling and activation in response to antigen exposure. This amplified response to target antigen may mitigate some of the dampening effects conferred by the suppressive factors that we tested. Notably, this heightened signalling response to antigen did not drive these cells towards dysfunction. Instead, RASA2 KO conferred a more persistent cancer killing capacity to T cells through repeated cancer antigen exposures. These heightened proximal signalling responses to repeated antigen encounters may drive changes in downstream transcriptional programmes that help preserve T cell function. For instance, we note that *RASA2* ablation leads to higher AP-1 and NF-κB transcriptional programmes with less pronounced differences in NFAT responses. This pattern is predicted to counteract T cell anergy and/or exhaustion, which can result from unopposed NFAT signalling^16^. Additionally, we observed transcriptional reprogramming toward metabolic states favouring oxidative phosphorylation, which were confirmed by functional analyses of mitochondrial fitness after chronic antigen exposures, suggesting that *RASA2* ablation may prevent dysfunction by altering the metabolic state of T cells. We also find that *RASA2* levels are elevated across multiple models of chronic stimulation. Although to our knowledge RASA2 had not previously been ascribed a role in T cell biology, we show here that RASA2 serves as a key intracellular checkpoint of T cell signalling and that its ablation leads to increased antigen sensitivity and persistent effector function in engineered human T cells.

Our work highlights *RASA2* as a promising genetic target for engineering improved next-generation T cells across indications. *RASA2* loss boosted T cell responses against antigen-dim target cells in vitro, which could greatly expand the repertoire of antigen receptors available in the clinic by widening the dynamic range of T cell signalling. Further preclinical testing is warranted to explore the efficacy and safety of *RASA2* ablation in T cell therapies. A concern might be that *RASA2* loss-of-function mutations, although uncommon, have been implicated in a subset of cancers, most prominently melanoma and multiple myeloma. However, it is notable that *RASA2* is usually co-mutated with other tumor suppressors (such as NF-1), suggesting reduced transformation potential as a single mutation^25^. Use of genome-targeted CAR integration with CRISPR may help to reduce the risk of insertional mutagenesis in additional genes that could serve as tumour suppressors, which is possible with lentiviral or retroviral CAR transduction. Of note, our observation that the fitness advantage in these RASA2-KO T cells is stimulation-dependent indicates that *RASA2* loss increases antigen sensitivity without driving constitutive proliferation. This stimulation dependence may be linked to the PH domain in RASA2, which binds to the lipid second messenger phosphatidylinositol (3,4,5)-trisphosphate but not phosphatidylinositol (4,5)-bisphosphate^49^. Phosphatidylinositol (3,4,5)-trisphosphate is present only in the active state, and recruits RASA2 to the plasma membrane, suggesting that the GAP activity of RASA2 is dependent on active PI3K signalling^24^. This dependence on PI3K signalling suggests that RASA2 may function as an inducible negative regulator of RAS signalling in the setting of cellular activation. Although this stimulation-dependence mitigates some concerns of using *RASA2*-deficient T cells therapeutically, these cells could also be engineered with suicide switches and synthetic circuits for tighter control over the T cell products^50^. Our data identifies RASA2 as a powerful regulator of T cell responses, and ongoing work will be needed to test for enhanced reactivity for unintended antigen targets across varying TCRs and CARs. Notably, the combination of RASA2 knockout with TRAC CAR knockin, which eliminates the endogenous TCR, should reduce the risk of enhancing potentially autoreactive T cells and improve safety^45,46^. Additional TCR-positive cell-depletion strategies could further reduce this risk. Overall, our findings demonstrate that *RASA2* ablation increases the potency and the persistence of T cell therapies, two key domains in which these therapies have been failing clinically. Combined with conferring resistance to suppressive cues, this makes *RASA2* ablation a promising new strategy for generating more effective T cell therapies for haematological and solid tumour indications.

## Methods

### Isolation of primary T cells from healthy donors

Leukopaks from deidentified healthy donors with Institutional Review Board-approved consent forms and protocols were purchased from StemCell Technologies (200-0092). For screens, residuals from leukoreduction chambers after Trima Apheresis from deidentified healthy donors with Institutional Review Board-approved consent forms and protocols were purchased from Vitalant (formerly known as Blood Centers of the Pacific). Primary Human T cells were isolated using EasySep Human T cell isolation kit (17951) according to the manufacturer’s protocol using the EasySep magnets. The cells were seeded in appropriate culture vessels and activated with Immunocult (Stem Cell Technologies, 10971) at 12.5 μl ml^−1^. Cells were kept in culture at a 10^6^ cells per ml density throughout, and cultured with IL-2 at 50 IU ml^−1^ (unless otherwise specified). Cells were cultured in X-Vivo-15 medium which was supplemented with 5% fetal calf serum, 50 µM 2-mercaptoethanol, and 10 mM *N*-acetyl-l-cysteine. Peripheral blood mononuclear cells (PBMCs) were frozen down at 5 × 10^7^ cells per vial using Bambanker (Bulldog Bio) serum-free cell freezing medium.

### Pooled CRISPR-KO screens under suppressive conditions

Pooled CRISPR-KO screens were performed as previously described^13^. In brief, isolated T cells were stimulated as above and 24 h later they were transduced with a lentiviral pool to express the genome-wide Brunello sgRNA library^51^. 24 h after transduction, T cells were washed once with PBS, electroporated with Cas9 protein and expanded in culture as above. On Day 14, T cells were stained with CFSE and stimulated with Immunocult in the presence of either tacrolimus (TOCRIS 3631, final concentration 5 nM), cyclosporine (TOCRIS 1101, final concentration 50 nM), CGS-21680 (TOCRIS 1063, final concentration 20 µM) or TGFβ1 (Biolegend 781802, final concentration 10 ng ml^−1^). For the T_reg_ cell condition, matched donor CD4^+^CD127^low^CD25^+^ T_reg_ cells were isolated on day 0 using magnetic enrichment (STEMCELL 18063), stimulated with anti-CD3/CD28 and expanded in culture until being mixed at a 1:1 ratio with the CFSE-stained effector T cells. For all screens, 3 days after re-stimulation, stained T cells were sorted into CFSE high and low populations and lysed, and genomic DNA was prepped for next-generation sequencing for each sample as previously described^13^. We used four human donors for the stimulation and T_reg_ cell screen, two donors for the adenosine, cyclosporine and tacrolimus screens, and one donor for the TGFβ screen. Screen hits were identified using MAGeCK^52^ v0.5.9 using paired analysis with default parameters. For tacrolimus and cyclosporine only, dividing cells were collected and compared to the undivided cells from the matched donors in the stimulation only screen. Guides with a read count of under 50 in more than 80% of the samples were filtered out. Supplementary Table 1 details the guide and gene-level counts, log fold change and MAGeCK scores. To find shared hits, gene-level log_2_ fold-change values were scaled to obtain *z*-scores. Genes above a *z*-score of the 95% percentile (*z*-score >1.54) were defined as hits for the shared hits analysis to generate Fig. 1b and Extended Data Fig. 1c and are detailed in Supplementary Table 1. To define suppressive condition-specific hits, the sgRNA counts in CFSE-low (highly dividing) cells were compared to the stimulation only (stim) condition using MAGeCK software as above. Results of this analysis are provided in Supplementary Table 2. For the quality metric of screens by dropout analysis of essential genes, we used essential genes as determined by DepMap^19^ and GSEA for gene-level log_2_ fold change. For analysis of expression of screen hits in primary human T cells the DICE database was used, averaging the expression of both activated CD4^+^ and CD8^+^ T cells^20^.

### CRISPR KO in primary human T cells using Cas9–RNP electroporation

T cells were isolated and stimulated as above and 48 h later, Cas9–sgRNA–RNP electroporation was performed using the Amaxa P3 Primary Cell 96-well 4D-Nucleofector Kit (Lonza, V4SP-3960). Lyophilized crRNA and tracrRNAs (Dharmacon) were resuspended in nuclease-free duplex buffer (IDT 1072570) at a concentration of 160 μM. Unless otherwise stated, control-edited T cells were targeted with the *AAVS1* sequence GGGCCACTAGGGACAGGAT, and *RASA2*-edited T cells were targeted with the *RASA2*-targeting sequence AGATATCACACATTACAGTG. In some cases, as detailed in the figure legends, the ctrl group indicates the non-targeting control guide GGTTCTTGACTACCGTAATT. The crRNAs and tRNAs were complexed at 1:1 v/v ratio for 30 min at 37 °C. sgRNAs were mixed with Cas9 (Stock 40 μM) at a 1:1 v/v ratio and incubated at 37 °C for 15 min to form the RNP complex. T cells were counted, resuspended in P3 buffer at 1 × 10^6^ per 20 μl, mixed with 3 μl of RNPs and added to a 96-well electroporation plate. The cells were electroporated using the EH115 protocol and immediately recovered by adding 80 μl T cell medium (X-Vivo-15, Lonza) at 37 °C for 15 min. Once recovered, cells were transferred to appropriate culture vessels in X-Vivo-15 medium with IL-2 at 50 IU ml^−1^.

### Cell line authentication and testing

Cell line sources were as follows: A375 (ATCC, CRL-1619), A375-CD19 (generated in this study), T2 cells (ATCC, CRL-1992), Nalm6 cells expressing luciferase, GFP and varying levels of CD19 (generated by J.E.), Nalm6 cells expressing NY-ESO-1 (generated by J.E.), Nalm6 cell line (originally purchased from ATCC, CRL-3273), LM7 osteosarcoma cells (kindly provided to G.K.’s lab by Eugenie Kleinerman of the MD Anderson Cancer Center in 2011), Jurkat reporter cells (gift from Kole Roybal of the University of California, San Francisco), Jurkat cells (originally purchased from ATCC, clone E6-1), HEK293T cells (Lenti- XTM 293T cell line, Takara Bio catalogue no. 632180). Certificates of analysis were provided with cell lines from ATCC and Takara Bio. Relevant antigen expression for each cell line was routinely confirmed by flow cytometry. LM7 cells were routinely validated using the ATCC STR Profiling Cell Authentication Service. The following cell lines were tested for mycoplasma: Nalm6, A375, LM7 and 293T cells. They were mycoplasma free as tested using either the LookOut Mycoplasma PCR Detection Kit (Sigma Aldrich, catalogue no. MP0035) at UCSF or the MycoAlert Mycoplasma Detection kit (Lonza, catalogue no. LT07- 218) at St. Jude. The following cell lines were used for short-term assays and not tested for mycoplasma: T2, Jurkat reporter lines. Our results pertain to the performance of primary human T cells. The International Cell Line Authentication Committee register was consulted and no commonly misidentified lines were used.

### Lentiviral production and T cell transduction of TCR

Lenti-X 293T cell line (Takara Bio 632180) cells were seeded at 18–20 million cells per 15 cm dish pre-coated with poly-l-lysine 16 h before transfection and cultured in DMEM + 5% FBS + 1% penicillin-streptomycin. Cells were transfected with the sgRNA transfer plasmids and second-generation lentiviral packaging plasmids, pMD2.G (Addgene 12259) and psPAX2 (Addgene 12260) using the Lipofectamine 3000 transfection reagent per manufacturer’s protocol (L3000001). Six hours after transfection, the transfection medium was replaced with DMEM + 5% FBS + 1% penicillin-streptomycin containing viral boost reagent at 500× per the manufacturer’s instructions (Alstem VB100). Twenty-four- and forty-eight-hour viral supernatants were collected and spun down at 300*g* for 10 min at 4 °C to remove the cell debris. The lentiviral particles were concentrated using Alstem precipitation solution (Alstem VC100) and stored overnight at 4 °C. The virus was centrifuged at 1,500*g* for 30 min at 4 °C and resuspended at 100× of the original volume in ice-cold PBS and stored at −80 °C until further use. For T cell transduction, 24 h after TCR stimulation, the concentrated lentivirus was directly added to T cells at 1:25 v/v ratio with X-Vivo-15 medium and gently mixed by tilting.

### CRISPR knock-in of CD19 CAR into *TRAC* using adeno-associated virus

Adeno-associated virus (AAV)-ITR plasmids containing the CD19 1928z CAR and *TRAC-*targeting homology arms for homology directed repair were used as previously described^46^. The AAV-ITR containing plasmid was packaged to AAV6 by transfection of HEK293T cells together with pHelper and pAAV Rep-Cap plasmids using Polyethylenimine. The AAVs were further purified using iodixanol gradient ultracentrifugation. The titration of the AAV was performed by quantitative PCR on DNaseI (NEB) treated, proteinase K (Qiagen)-digested AAV samples, using primers against the left homology arm (forward: CTTTGCTGGGCCTTTTTCCC, reverse: CCTGCCACTCAAGGAAACCT). The quantitative PCR was performed with SsoFast EvaGreen Supermix (Bio-Rad 1725201) on a StepOnePlus Real-Time PCR System (Applied Biosystems).

T cells were isolated and activated as previously described^46^. After 48 h of T cell activation, cells were transfected by electroporation of RNP using a 4D Nucleofector 96 well unit (Lonza). One reaction of RNP was generated by incubating 60 pmol of Cas9 protein with 120 pmol sgRNA (Synthego, TRAC guide RNA (gRNA): ACAGGGUUCUGGAUAUCUGU) at 37 °C. Two million cells were electroporated and diluted into culture medium and incubated at 37 °C, 5% CO_2_. Recombinant AAV6 donor vector was added to the culture 30 to 60 min after electroporation, at the indicated multiplicity of infection (10^5^), and incubated with the cells overnight. The day after the electroporation, edited cells were resuspended in T cell growth medium and expanded using standard culture conditions and kept at a density of 10^6^ cells per ml. Knock-in efficiency was evaluated by flow cytometry by staining the CAR with a goat anti-mouse Fab (Jackson ImmunoResearch, 115-606-003).

### In vitro cancer killing assay by TCR T and CAR T cells

Antigen-specific T cells were co-cultured with pre-plated RFP^+^ A375 or GFP^+^ Nalm6 tumour cells in a 96-well flat bottom plate starting at a 2:1 E:T ratio then with a log_2_ serial dilution in triplicates. For target cancer cells, A375 (ATCC, CRL-1619) were used for TCR T assays. For CAR T assays, CD19-expressing RFP^+^ A375 melanoma cells were generated by targeted non-viral knock-in of an SFFV promoter in front of the endogenous CD19 gene (guide targeting CATGGTGGTCAGACTCTCCG) as previously described^45^. Cells were sorted for uniformly low CD19 expression. Nalm6 cell lines with varying CD19 expression levels and Nalm6 cell line engineered to express the NY-ESO-1 antigen generated by J. Eyquem. For experiments with annexin detection, Annexin V Dyes (Essen Bioscience) Red (4641) and Green (4642) were used according to the manufacturer’s instructions. The plates were imaged every 2–3 h for 72–96 h using IncuCyte Zoom live-cell imaging (Essen Bioscience). The RFP^+^ or GFP^+^ object counts per well was recorded over time. Cancer cell growth was calculated as the count at any given time point, normalized by the count at *t* = 0. For time traces showing a confidence interval as a grey shaded area, traces were smoothed by fitting a generalized additive model using the R package gam v1.20. The fitted model was also used to interpolate the trace and calculate the area under the curve for the given time interval.

### Screen validation experiments

For the arrayed screen validation experiments, T cells were isolated from two donors and edited with RNPs with gRNAs targeting genes of interest or control guides as indicated above. Genes for this arrayed validation were selected based on log fold change over the stimulation condition, false discover rate (FDR), expression level in T cells, and relative log_2_ fold change across the different screens (Supplementary Table 3). Two gRNAs were selected per gene, one from the screen hits, and an orthogonal guide designed by an online tool (https://www.synthego.com/products/bioinformatics/crispr-design-tool). Guide sequences are detailed in Supplementary Table 3. At day 9 post isolation, T cells were stained with CFSE to track cell divisions and stimulated with 6.25 µl ml^−1^ Immunocult at 10^6^ cells per ml. Drug doses used for the validation of gene targets were as follows: cyclosporine, 50 nM; tacrolimus, 0.25 nM; CGS-21680, 100 µM; TGFβ, 10ng ml^−1^. For the functional cancer cell killing assays, the IncuCyte system was used as above. For validation of the T_reg_ cell resistance, T_reg_ cells were isolated as above and mixed with donor-matched CFSE-stained effector T cells at varying cell to cell ratios. For the functional cancer cell killing assays, the IncuCyte system was used as above.

### Western blot for active RAS and for phosphoproteins

For immunoblotting experiments, T cells were serum starved for 2 h at 37 °C in RPMI (Gibco 21870076). After starvation, cells were stimulated for 0, 5, 10, 30 and 60 min with Immunocult at 12.5 μl ml^−1^ at 37 °C in a water bath. After each timepoint, the stimulation was quenched with ice-cold PBS and the cells were spun down at 300*g* for 5 min at 4 °C. Each pellet was resuspended in Pierce RIPA buffer (Thermo Fisher 89901) and incubated at 4 °C for 40 min. Cell lysates were stored at −80 °C until further use. The protein concentrations were determined using Pierce BCA Protein Assay (Thermo Fisher 23227). Fifteen micrograms of protein per sample was loaded onto 4–15% tris-glycine SDS gels (Bio-Rad) followed by transfer to PVDF membrane (Bio-Rad) using the Biorad Trans-Blot Transfer system. The membrane was blocked using 5% milk in TBST and incubated with primary antibodies at 4 °C overnight. Primary antibodies used: p-ERK (4370), p-MEK (9154) (Cell Signaling Technology), vinculin (MAB3574) (Millipore Sigma), RASA2 (HPA035375) (Sigma Aldrich), β-actin rabbit monoclonal (horseradish peroxidase (HRP) conjugate) (Cell Signaling 5125), anti-rabbit HRP antibody (Cell Signaling 7074), anti-mouse IgG, HRP-linked antibody (Cell Signaling 7076), RASA2 rabbit anti-human GAP1m (NBP1-89794 Novus Biologicals), GAPDH mouse anti-human GAPDH (sc-47724 Santa Cruz Biotechnology), goat anti–rabbit IgG–HRP (111-036-045 Jackson ImmunoResearch) and goat anti–mouse IgG–HRP (sc-2005 Santa Cruz Biotechnology).

Membranes were imaged on the Azure Biosystems 600 imaging system at UCSF, and they were imaged on the Odyssey Fc Imaging System (LI-COR Biosciences) at St Jude.

For the active RAS assay, T cells and Jurkat cells (ATCC (Clone E6-1)) were serum starved for 2 h at 37 °C in RPMI (Gibco 21870076). After starvation, T cells were stimulated for 5 min with Immunocult at 12.5 μl ml^−1^ at 37 °C in a water bath. Once incubation was complete, the stimulation was quenched with ice-cold PBS and the cells were spun down at 4 °C at 300*g* for 5 min. Further assay was performed according to protocol from RAS Activation Assay kit (Cytoskeleton BK008).

For densitometry analysis of the western blot gels, we used ImageJ v1.52q software. Using the set measurements option under the ‘Analyze’ menu, we set the measurements as mean grey value for the analysis. Next, a region of interest (ROI) was defined using the rectangle tool to draw a frame around the band of interest. The same ROI was used to quantify all of the bands. Using the same frame as the protein, measurements for the background were taken for that protein. We repeated the steps for loading controls and recorded the measurements. For the analysis, the pixel density was inverted for bands/controls and their backgrounds expressed as 255 − *X*, where *X* is the value for the protein band or loading control band. The net value for protein band and the loading control was calculated by subtracting the inverted background value from the inverted band value. The relative quantification value is calculated as a ratio of net protein band value to net loading control band value of that lane.

### Flow cytometry assays

For cell surface activation markers, 2 × 10^5^ to 5 × 10^5^ TCR T cells were seeded per well in a round bottom 96-well plate. The TCR T cells were stimulated by Immunocult at 12.5 μl ml^−1^ at 37 °C for 4–6 h. CAR T cells were stimulated by co-culturing with CD19^+^ Nalm6 leukaemia cells at 1:1 E:T ratio at 37 °C for 4–6 h. Next, the cells were centrifuged, washed once with 200 μl of cell staining buffer and stained with antibodies (5 µl antibody per in 100 μl staining buffer) for 30 min at 4 °C in the dark. Samples were read using Attune NXT Cytometer (Invitrogen) and analyzed by FlowJo 10.7.1. For exhaustion and differentiation markers, cells were not stimulated but stained as described above. Antibodies used: Brilliant Violet 421 CD69 (Biolegend 310930), FITC anti-human CD154 (Biolegend 310804), PE anti-human CD25 (Biolegend 302606), FITC anti-human CD279 (PD-1) (Biolegend 621612), Brilliant Violet 711 CD223 (LAG-3) (Biolegend 369320), Brilliant Violet 421 anti-human CD366 (Tim-3) (Biolegend 345008), PE anti-human CD39 (Biolegend 328208), PE anti-human CD62L (Biolegend 304806), PE CD19 (Beckman Coulter IM1285U) and APC CD19 (Beckman Coulter IM2470U).

For phospho-flow cytometry assays, 2 × 10^5^ to 5 × 10^5^ TCR T cells were seeded per well in a round bottom 96-well plate (Corning 877254) and stimulated by Immunocult at 12.5 μl ml^−1^ for 5, 10 and 30 min at 37 °C. For CAR T cells staining, CAR T cells were activated by co-culture with CD19^+^ Nalm6 leukemia cells at 1:1 E:T ratio in a round bottom 96-well plate, spun down briefly at 400*g* and incubated at 37 °C for 5 min. At the end of treatment, the cells were fixed with pre-warmed BD Phosflow Fix Buffer I (BD Biosciences 557870) for 10 min at 37 °C. Cells were washed once with Stain Buffer (FBS) (BD Biosciences 554656). Next, the cells were permeabilized by adding BD Phosflow Perm Buffer III and incubated 30 minutes to overnight at −20 °C. Cells were then washed twice and incubated with antibodies (5 μl antibody per in 100 µl staining buffer) for 30 min at room temperature in the dark followed by two washes with Stain Buffer (FBS). Samples were read using an Attune NXT Cytometer (Invitrogen) and analysed by FlowJo 10.7.1. Antibodies used: anti-MEK1 (pS218)/MEK2 (pS222) (BD 562460), Alexa Fluor 488 anti-ERK1/2 phospho (Thr202/Tyr204) (Biolegend 675507), PE anti-ERK1/2 phospho (Thr202/Tyr204) (Biolegend 369506), Brilliant Violet 421 anti-RPS6 phospho (Ser235/Ser236) (Biolegend 608610), PE Mouse anti-4EBP1 (pT36/pT45) (BD 560285), Brilliant Violet 421 Anti-AKT (pS473) (BD 562599), PE anti-p38 MAPK phospho (Thr180/Tyr182) (Biolegend 690204).

For intracellular cytokine staining, TCR T cells were stimulated by Immunocult at 12.5 μl ml^−1^ and brefeldin A (eBioscience 00-4506-51) at 37 °C for 4-6 h. For CAR T cells stimulation, T cells and CD19^+^ Nalm6 leukemia cells were co-cultured at 1:1 E:T ratio and incubated with brefeldin A at 37 °C for 4–6 h. Next, cells were fixed and permeabilized with Fix & Perm Cell Permeabilization Kit (Thermo Fisher Gas004) and incubated with fluorochrome-conjugated antibodies (5 µl antibody per in 100 µl staining buffer) for 20 min at room temperature in the dark. Samples were read using Attune NXT Cytometer (Invitrogen) and analysed using FlowJo 10.7.1. Antibodies used: PE mouse anti-human IFNγ (BD Biosciences 554701), BV711 mouse anti-human IL-2 (BD Biosciences 563946), Pacific Blue anti-human TNF (Biolegend 502920).

For Mitotracker probe staining, T cells were incubated in a 96 well plate at 200,000 cells per well in 25 nM mitotracker Green FM (M7514) or Mitotracker Red CMXRos (M7512) in 100 µl of warm X-Vivo medium in the incubator for 30 min. Cells were then quenched with warm complete X-vivo medium at a 1:1 volume, spun down, washed twice with warm X-vivo medium, resuspended in 5% FBS/PBS, and then analysed on the Attune flow cytometer.

Antibodies and reagents used for flow cytometry experiments on cells isolated from bone marrow included: PE-Cyanine7 anti-human CD8a (eBioscience 25-0087-42), APC-Cy7 mouse anti-human CD45 (BD Biosciences 557833), BUV395 mouse anti-human CD4 (BD Biosciences 563550), BV421 mouse anti-human CD62L (BD Biosciences 563862), BV650 mouse anti-human CD45RA (BD Biosciences 563963), BV480 mouse anti-human CD279 (PD-1) (BD Biosciences 566112), PerCP-eFluor 710 anti-human CD223 (LAG-3) (eBioscience 46-2239-42), BUV737 mouse anti-human CD19 (BD Biosciences 564303), BV785 anti-human CD366 (Tim-3) (Biolegend 345032), PE CD127 (IL7RA) (Biolegend 351304), PE anti-human EGFR (Biolegend 352904), 7-AAD (Invitrogen A1310), Counting Beads (Invitrogen C36995). For these antibody stains, cells were resuspended in 2 µl antibody in 100 µl total staining buffer volume (see ‘Bone Marrow CAR T cell isolation, processing and staining’).

### Titration assay to measure antigen dose–response curve

T cells were isolated, activated, transduced with lentivirus containing the NY-ESO-1 1G4 TCR and edited for RASA2 or AAVS1 as described above. TCR T cells (2 × 10^5^ to 3 × 10^5^) were seeded per well and stimulated with Immunocult. For pERK staining, the T cells (both transduced and untransduced as a control) were stimulated for 2 min, 5 min, 10 min at 37 °C with top dose of Immunocult at 50 μl ml^−1^ and serially diluted by 2. For activation markers, T cells were stimulated for 24 h at 37 °C with top dose of Immunocult at 12.5 μl ml^−1^ and serially diluted by 2. Subsequent staining was performed as described above.

To measure T cell sensitivity to antigen-specific TCR re-stimulation, primary human T cells were stimulated with Immunocult, after 24 h then transduced with concentrated lentivirus to express the NY-ESO-1-specific T cell receptor, and 24 h later RNP-edited for RASA2 or AAVS1 as described above. Antigen-presenting T2 cells^53^ (ATCC CRL-1992) were loaded with the NY-ESO-1_157-165_ peptide SLLMWITQV (Thermofisher) by incubation at 37 °C for 1 h. Top final dose of peptide was 18 μM and was subsequently diluted by log5 serial dilutions. Unbound peptide was removed by washing twice with medium. Antigen-specific edited T cells were added and allowed to interact with the T2 cells for 10 min before the reaction was stopped by adding a fixation buffer for pERK staining using flow cytometry as above.

### Transcriptional reporter using Jurkat cell lines

Jurkat T cell reporter systems for activator protein 1 (AP-1), nuclear factor of activated T cells (NFAT), and nuclear factor κB (NF-κB) transcriptional activity were a gift from Kole Roybal (University of California, San Francisco) and were generated as previously described^54^. These Jurkat reporter cells were TCR-stimulated with Immunocult at 12.5 μl ml^−1^, the top dose then serially diluted by 2 for a range of activation levels. Cells were assayed for mCherry levels every 24 h using flow cytometry.

### Generation of transgenic *RASA2*

To create a transgenic *RASA2* construct to use for the RASA2-overexpression experiments, RASA2 ORF (NM_001303246.2, GenScript) was cloned into a retroviral pSFG vector with a Flag tag added to the N terminus using In-Fusion cloning kit (Takara). A GFP or CAR transgene was inserted into the same retroviral pSFG backbone as RASA2 for use as a transgene overexpression control. To generate T cells expressing transgenic RASA2, cells were transduced using the same protocol used for CAR T cell generation. In brief, retroviral particles were generated by transient transfection of HEK293T cells with the RASA2-encoding SFG retroviral vectors, Peg-Pam-e plasmid encoding MoMLV gag-pol, and a plasmid encoding the RD114 envelope protein. Supernatants were collected after 48 h, filtered and snap-frozen for later transduction of T cells. RASA2 expression was confirmed by western blot.

### Repetitive stimulation assay

Tumour cells were seeded in complete RPMI medium one day prior to co-culture. Complete RPMI medium includes RPMI (Gibco 21870076), 10% fetal bovine serum, 1% l-glutamine, 1% penicillin-streptomycin. The next day, RPMI medium was replaced with T cell medium and antigen-specific T cells were seeded on top of the tumour cells at a 1:1 E:T ratio with IL-2 at 50 IU ml^−1^. Subsequent repeated co-cultures were set up every 48 h. For each co-culture, T cells were collected and counted using the Vi-CELL XR cell counter and viability analyser and replated onto fresh target tumour cells at a 1:1 E:T ratio. Before using the T cells for any assays, T cells were collected, counted and purified using EasySep Release Human CD45 positive selection kit (Stem Cell 100-0105) or purified by flow sorting. For ELISA experiments, after 5 stimulations of TCR T cells with target cells (A375), supernatant of co-cultures was collected and analyzed using the LEGENDplex Human CD8/NK Panel 13-plex (Biolegend 740267) according to the manufacturer’s instructions.

### Gene expression analysis using RNA-seq

T cells were subjected to the repeated stimulation assay in 24-well plates, transferring T cells to freshly seeded cancer cells every 48 h. Cultured T cells were sorted after each stimulation using BD FACSAria Fusion to obtain a pure population of NY-ESO-1 multimer positive cells T cells from the co-cultures with target cancer cells, and resuspended in TRI Reagent (Sigma T9424). Total RNA was extracted using Direct-zol RNA MicroPrep kit (Zymo Research R2061) per the manufacturer's protocol and prepared for sequencing as previously described^55^, by the Functional Genomics Laboratory at UC Berkeley and sequenced by the Vincent J. Coates Genomics Sequencing Laboratory at UC Berkeley. To evaluate kinetics of *RASA2* gene expression levels during the course of the repetitive stimulation assay, NY-ESO-1 TCR or anti-CD19 CAR T cells were stimulated with target cells 5 times and TCR^+^ or CAR^+^ cells were FACS sorted 48 h after each stimulation (NGFRt reporter). Cells were resuspended in TRI Reagent (Sigma T9424). Total RNA was extracted using Direct-zol RNA MicroPrep kit (Zymo research R2061) per the manufacturer's protocol and prepared for sequencing as previously described^55^, by Functional Genomics Laboratory at UC Berkeley and sequenced by the Vincent J. Coates Genomics Sequencing Laboratory at UC Berkeley. To compare gene expression between *RASA2*- and control-edited T cells, cells were subjected to the repeated stimulation assay as above and isolated after five stimulations using Releasable Human CD45 Positive Selection Kit (Stemcell technologies 100-0105), pelleted and sent in RNAlater (Thermofisher, AM7020) to the DNA Technologies and Expression Analysis Cores at the UC Davis Genome Center for batch-tag-seq RNA-seq.

To analyse the gene expression, reads were mapped to the human reference transcriptome (GRCh38 Ensembl release 96) using Kallisto^56^ with default parameters. Genes with zero counts in more than 80% of the samples were filtered out. Differential gene expression was performed using R package DESeq2^57^ v1.32.0, controlling for donor variance. Results of differential gene expression analysis are provided in Supplementary Table 4. The R package fgsea^58^ v1.18.0 was used to perform GSEA, with gene ranking based on DESeq2 test statistic and MSigDB v7.2 hallmark gene sets^59^ as the reference gene lists.

### Analysis of published gene expression datasets from GEO and BioGPS

To define tissue-specific expression of the RasGAP family of genes, the Human U133A Gene Atlas was downloaded from BioGPS website and probe id was matched to Gene Symbol using BioMart. To allow for comparison between genes, expression values for each gene were scaled to a minimum of 0 and a maximum of 1. Only the top 2% expressing tissues are labelled in Extended Data Fig. 1h. Data are shown for all RasGAP family members available in this dataset.

To find genes correlated with *RASA2* expression in immune cells (Extended Data Fig. 5e,f), the R package correlationAnalyzeR^60^ v1.0.0 was used. Only datasets defined as ‘normal’ (not tumor) and ‘immune’ tissues were used to find the Pearson’s correlation coefficient (R) with RASA2 expression. The signed correlation coefficient was used to rank all genes based on their correlation with *RASA2* expression and was analysed for GSEA as above.

To generate Fig. 2k, our published scRNA-seq CROP-seq data from primary human T cells was downloaded from the Gene Expression Omnibus (GEO) accession GSE119450 and processed to generate gene expression and sgRNA barcode matrices as previously described^13^. Differentially expressed genes between stimulated cells expressing *RASA2* sgRNA and non-targeting control guide (ctrl) were analysed using the FindMarkers function from Seurat^61^ 4.0 R package. Only cells from the stimulated samples with either *CD3D*, *CBLB*, *RASA2* or non-targeting (ctrl) sgRNA were used to calculate the average expression for each gene in Fig. 2k.

To generate Fig. 2l,n and Extended Data Fig. 5h,i, processed RNA-seq datasets were downloaded from GEO (GSE89307, GSE86881 and GSE138459, respectively). For each dataset, expression of *RASA2* and *PDCD1* was extracted from the count matrices and scaled to a minimal value of 0 and a maximal value of 1 to allow for inter-gene comparison. For Fig. 2m, expression data for RASA2 was downloaded from the DICE database^20^ (https://dice-database.org/). For human TIL data (Fig. 2o), RASA2 expression data was downloaded from the web portal for each dataset (http://crc.cancer-pku.cn and http://lung.cancer-pku.cn). Only data from cells labelled as ‘CD8 T cells’ from the peripheral and tumor samples were used for analysis.

### Seahorse assay

Metabolic phenotyping was performed by extracellular flux analysis. Mitochondrial substrate dependency and maximal respiration levels were determined by assessing OCR. OCR was measured using a 96-well extracellular flux analyser (Seahorse Bioscience). In brief, TCR T cells or CAR T cells were isolated from culture using a CD45 isolation kit (100-0105), plated on pre-coated 96 well plates that were coated with poly-d-lysine (103729-100) at 4 × 10^5^ cells per well in 50 μl Seahorse XF RPMI supplemented with 10 mM glucose, 1 mM pyruvate and 2 mM glutamine per manufacturer’s instructions (Seahorse XF RPMI assay medium pack 103681-100). After plating, cells were left in the incubator for an hour to ensure adherence to the wells. After inspection under the microscope confirming uniform and confluent adherence, 130 μl of the supplemented Seahorse XF RPMI medium was added to bring the volume to 180 μl per well. These plated cells were then placed for 1 h in a CO_2_-free incubator at 37 °C before commencing measurements using the Seahorse instrument. The Seahorse Mito stress test assay was performed using the Seahorse XF Cell Mito Stress Test Kit (103015-100), and the substrate oxidation stress tests were performed using the Agilent XF Substrate Oxidation Stress Test Kits, specifically, the XF Long Chain Fatty Acid Oxidation Stress Test Kit (103672-100), the XF Glucose/Pyruvate Oxidation Stress Test Kit (103673-100) and the XF Glutamine Oxidation Stress Test Kit (103674-100). Drugs were used in the following final concentrations: oligomycin, 1.5 μM; carbonyl cyanide-*p*-trifluoromethoxyphenylhydrazone (FCCP), 1 μM; rotenone + antimycin A, 0.5 μM; etomoxir, 4 μM; UK5009, 2 μM; and BPTES 3 μM. Experiments were performed according to the manufacturer’s instructions. All Seahorse assays were run on a Seahorse XFe96 Analyzer.

### NY-ESO-1 TCR T cells with A375 or Nalm6 xenograft models

Eight- to ten-week-old male or female NOD-SCID-*Il2rg*^*−/−*^ (NSG) mice (*Mus musculus*, strain #005557) were purchased from Jax or bred in-house. All mice were housed and treated in ethical compliance with UCSF IACUC approved protocols. Mice were housed in the UCSF LARC Animal Care Facilities at the Helen Diller Family Cancer Center or at UCSF Parnassus. They were housed in an individual specific-pathogen free suite. They were housed with up to 5 mice per cage in ventilator cages, with ad libitum food and water on a 12-hour light cycle and controlled temperature and humidity conditions (19–23 °C and 30–70%). IACUC protocols used at UCSF included the UCSF Preclinical Therapeutics Core (IACUC protocol AN194778—continuation of AN179937) and the Marson laboratory (AN180228-03B). To ensure equivalent pre-treatment tumour burdens, mice were randomized by tumour burden prior to T cell injections. For the A375 melanoma xenograft study, NSG mice were engrafted with 1 × 10^6^ A375 melanoma cells via subcutaneous injection on day 0. On day 7, 1 × 10^6^ NY-ESO-1 TCR^+^ T cells were infused via intravenous injection in the tail vein. A375 cell progression was measured by calliper measurements. For the NY-ESO-1^+^ Nalm6 leukemia study, NSG mice were injected intravenously with 0.3 × 10^6^ NY-ESO-1-expressing Nalm6 leukaemia cells on day 0, followed by 0.5 × 10^6^ control-edited or RASA2-KO NY-ESO-1-specific TCR T cells on day 4. Bioluminescence imaging was conducted using the Xenogen IVIS Imaging System (Xenogen) with Living Image software (Xenogen) for acquisition of imaging datasets. Mice were humanely euthanized at an IACUC-approved end-point when tumour measurement reached 2 cm in the largest dimension or when they demonstrated signs of morbidity described in our IACUC protocol, such as respiratory distress, hunched posture, lesions unresponsive to treatment, body condition score of 2 or less, 15% weight loss, impaired or decreased mobility, tumour ulcerated or interfering with normal functions, neurologic signs that interfere with normal function, reduced grooming and piloerection, or rectal prolapse. These limits were not exceeded in any of the experiments. For all in vivo experiments, mice allocated to different experimental groups were sex-, age- and housing-matched. No statistical methods were used to predetermine sample size. Sample sizes were estimated on the basis of preliminary experiments and previously published results. We made an effort to achieve a minimum sample size of *n* = 5 mice per treatment arm, which proved to be sufficient to reproducibly observe statistically significant differences. Sample size is stated in each legend. For all in vivo experiments, mouse randomization and injections were always done by a blinded member of the Preclinical Therapeutics Core or a blinded member of the Marson laboratory. Measurements of tumour burdens, monitoring of the mice, and tumour burden analysis were performed by members of the Preclinical Therapeutics Core who were blinded to the experimental groups. For the in vivo cell phenotyping and rechallenge experiments, when Preclinical Therapeutics Core staff were not available, J.C. collected the data and was not blinded to the groups. For the histopathological analysis of bone marrow and splenic tissues, the hematopathologist was blinded to the experimental groups.

### CD19-CAR T cells and NALM6 xenograft model

Animal experiments followed a protocol approved by the UCSF Institutional Animal Care and Use Committee. Eight- to twelve-week-old NOD/SCID/IL-2Rγ-null (NSG) male mice were obtained through Jax Labs or in-house breeding. Mice were intravenously injected with 0.5 × 10^6^ FFLuc-GFP NALM-6, followed four days later by intravenous injection with 0.1–0.2 × 10^6^ CD19-specific CAR T cells. Tumour burden was monitored via bioluminescence imaging as above. For all in vivo CAR T cell experiments, mice were randomized on the basis of bioluminescence imaging results to ensure equal tumour burden distribution in each group before T cells were transferred.

Two separate cohorts of NSG mice were treated in parallel to evaluate the effects of the CAR T cells alone on the health of the mice. The first cohort was injected with 0.2 × 10^6^ CD19-specific CAR T cells edited for RASA2 versus a control guide as described above. The second cohort served as an antigen-experienced cohort, where NSG mice were engrafted with 0.5 × 10^6^ FFLuc-GFP NALM-6 cells and then injected with CD19-specific CAR T cells at tumour-clearing doses. Mice were followed over time with bioluminescence imaging and monitored by weight and for any signs of morbidity per our UCSF IACUC protocol. Bioluminescence measurements showed that the cohort receiving NALM6 cells cleared these tumours by day 18. After prolonged monitoring, mice from these cohorts were euthanized on day 116. Mouse tissues (spleen, bone marrow and lymph nodes) were fixed in phosphate buffered formalin. Prior to embedding, sternae were decalcified with a formic acid containing commercially available decalcification solution. Tissues were paraffin-embedded, sectioned and stained with haematoxylin and eosin. Tissues were initially reviewed by a blinded member of the UCSF Preclinical Therapeutics Core for evidence of pathologic abnormalities without knowledge of the experiments from which the individual animal tissues were generated. Tissue slides were reviewed by a blinded UCSF haematopathologist. Complete blood counts were measured from blood from these mice using a Hemavet 950 instrument.

For the rechallenge experiment, a donor was tested and found to exhibit durable tumour control at the same CAR T cell doses of 0.2 × 10^6^ CAR^+^ CAR T cells per mouse. A separate cohort of mice were then engrafted with the same 0.5 × 10^6^ number of FFLuc-GFP NALM-6 cells, and then 4 days later they were intravenously injected with 0.2 × 10^6^ CD19 CAR^+^ CAR T cells edited for *RASA2* versus a control guide as before. In this case, after the CAR T cell injection, mice were then injected with 1 × 10^6^ FFLuc-GFP NALM-6 cells in three separate intravenous rechallenge injections 7–11 days apart. Mice were followed over time with bioluminescence imaging and monitored by weight and for any signs of morbidity per our UCSF IACUC protocol as listed above. These limits were not exceeded in any of the experiments.

### Bone marrow CAR T cell isolation, processing and staining

NALM-6-bearing mice were treated with 2 × 10^5^ TRAC CAR T cells from a donor previously tested for in vivo leukaemia control efficacy, and euthanized at days 7 and 16 after infusion. For bone marrow extraction, long bones of each leg were isolated by dissection, and then crushed with PBS in a mortar and pestle, followed by PBS washes through a cell strainer. Cells were then centrifuged and treated for 2 min with ACK lysing buffer (118-156-721, Quality Biological) for red blood cell lysis, the reaction was quenched with FACS buffer. Remaining cells per each mouse were then resuspended in 300 μl FACS buffer. After addition of Fc block (10 μl per sample) (130-092-575, Miltenyi Biotec), cells were stained for the CAR (1 μl per sample) (115-606-07, Jackson ImmunoResearch) and incubated for 30 min at room temperature. After a wash, cells were resuspended in 2% normal mouse serum (Millipore-Sigma) and Fc block (10 μl) and incubated for 20 min at room temperature. Next cells were stained with the relevant antibody mix (100 μl per tube staining volume; specific antibodies found above in the flow cytometry methods section) and incubated for 45 min at room temperature. After staining, cells were washed and resuspended in 300 μl FACS buffer as well as counting beads (50 μl per sample) (C36950, ThermoFisher Scientific) and then analysed by flow cytometry.

### In vivo CD19 CAR T cells competition experiment

CD19 TRAC CAR T cells were generated as described above, using the CAR construct described previously^46^. In brief, this construct contains a pAAV-TRAC-1928z containing 1.2 kb of genomic TRAC flanking the gRNA targeting sequence, a self-cleaving P2A peptide in frame with the first exon of TRAC followed by the 1928z CAR and a second self-cleaving P2A peptide. For this experiment, we made use of a version of this construct that either does or does not include a sequence for a truncated form of EGFR (EGFR-t), followed by a third self-cleaving P2A peptide. As above, these two different CAR T populations were edited for either the *AAVS1* locus or the *RASA2* locus. The resulting products included four CAR T cell populations, *RASA2-*edited CD19 TRAC CAR T cells with and without EGFRt, and *AAVS1-*edited CD19 TRAC CAR T cells with and without EGFRt. These populations were then combined into two mixed populations, mix 1 (*AAVS1*-edited CD19 TRAC CAR T cells without EGFRt + *RASA2-*edited CD19 TRAC CAR T cells with EGFRt) and mix 2 (*AAVS1-*edited CD19 TRAC CAR T cells with EGFRt + *RASA2-*edited CD19 TRAC CAR T cells without EGFRt). Mixing percentages of each of these two mixed populations were confirmed to be approximately 50%:50% in each mixed input population on the day of infusion into Nalm6-bearing mice. Mice were euthanized at days 7 and 16 after infusion, and bone marrow was processed as above. Final percentages of AAVS1-KO versus RASA2-KO CAR T cells isolated from the bone marrow at these time points were determined by staining for CAR and EGFR.

### EphA2-CAR RASA2-KO T cell generation

The generation of the retroviral vectors encoding the EphA2 CARs has been previously described^48,62^. Retroviral particles were generated by transient transfection of HEK293T cells which were seeded at 1–2 million cells per 10 cm dish 2 days before transfection and cultured in DMEM with 10% FBS. Cells were transfected with the CAR retroviral plasmid, retroviral packaging plasmids Peg-Pam3-E and a plasmid encoding the RD114 envelope protein using the GeneJuice transfection reagent per manufacturer’s protocol (Novagen 70967). Forty-eight hours after transfection, viral supernatants were collected, spun down at 400*g* for 5 min and filtered using 0.45-µm filter to remove the cell debris. Viral supernatants were then snap-frozen and stored at −80 °C until further use.

To generate EphA2-CAR RASA2-KO T cells, human PBMCs were obtained from whole blood of healthy donors under IRB-approved protocols at St Jude Children’s Research Hospital (SJCRH). To generate CAR T cells, we isolated PBMCs by Lymphoprep (Abbott Laboratories) gradient centrifugation. On day 0, PBMCs were stimulated on non–tissue culture–treated 24-well plates, which were precoated with CD3 and CD28 antibodies (anti-CD3/CD28; CD3: OKT3, CD28: 15E8; Miltenyi Biotec). Recombinant human IL-7 and IL-15 (IL-7: 10 ng ml^−1^; IL-15: 5 ng ml^−1^; PeproTech) were added to cultures on day 1. T cells were electroporated with ribonucleoproteins (RNPs) targeting *RASA2* using the Lonza 4D electroporator on day 2, and transduced with the EphA2 CAR-encoding retroviral vector on day 3. sgRNA was designed to target the sequence AGATATCACACATTACAGTG (g1), AGGATCGACTTGTGGAACAA (g2) or a non-targeting guide sequence AGTAGTCGGGATGTCGGCG. RNPs were precomplexed at an sgRNA:Cas9 ratio of 4.5:1, prepared by adding 3 μl of 60 μM sgRNA (Synthego) to 1 μl of 40 μM Cas9 (Macro Lab, University of California, Berkeley) and frozen for later use. For RASA2 KO, 1 × 10^6^ T cells were resuspended in 17 μl P3 nucleofector master solution (Lonza P3 Primary Cell 4D-NucleofectorTM X Kit S, V4XP-3032) and added to 3 μl RNP. Twenty microlitres of cells + RNP were electroporated using program EH-115. One 20 μl electroporation reaction was transferred to one well of a 48-well tissue culture treated plate containing RPMI 1640 supplemented with 20% FBS, 1% Glutamax, 10 ng ml^−1^ IL-7, and 5 ng ml^−1^ IL-15 (recovery medium) for overnight. The next day, CAR transduction was performed using electroporated cells.

For T cell transduction, 500 μl of viral supernatants were spun down on RetroNectin (Clontech) coated 24-well non-tissue culture plate at 2,000*g* for 90 min. After the spin, viral supernatants were removed and electroporated T cells were plated at 2.5 × 10^5^ cells per well in 2 ml of recovery medium. 48 h later, CAR-transduced T cells were transferred from the RetroNectin-coated plate to a tissue culture plate and expanded in RPMI 1640 supplemented with 10% FBS, 1% Glutamax, 10 ng ml^−1^ IL-7, and 5 ng ml^−1^ IL-15. T cells were evaluated for CAR expression with CD19-PE (clone J3-119, Beckman Coulter) 4–6 days after transduction. Untransduced T cells were used as a negative control for gating. Samples were washed with and stained in PBS (Lonza) with 1% FBS (Cytiva). LIVE/DEAD Fixable Aqua Dead Cell Stain Kit (Invitrogen, Thermo Fisher Scientific) was used as a viability dye. A FACSCanto II (BD) instrument was used to acquire flow cytometry data, which were analyzed using FlowJo v10.

### EphA2-CAR T cells and LM7 xenograft model

Animal experiments followed a protocol approved by the St Jude Children’s Research Hospital Institutional Animal Care and Use Committee. Eight- to -ten-week-old female NSG mice were obtained from the SJCRH NSG colony. Mice were housed in an individual specific-pathogen free individual suite at the St Jude Animal Resource Center (ARC) which is a fully AAALAC-accredited facility. The IACUC protocol used at St Jude in the laboratory of G.K. was 623-100650. The mice were housed up to 5 mice per cage in ventilator cages, with ad libitum food and water on a 12-h light cycle and controlled temperature and relative humidity conditions (19–23 °C and 30–70%). Mice allocated to different experimental groups were sex-, age- and housing-matched. Mice were humanely euthanized when an IACUC-approved end-point tumour measurement of bioluminescence reached >10^10^ photons s^–1^ or when mice demonstrated signs of distress described in our IACUC protocol such as respiratory distress, hunched posture, lesions unresponsive to treatment, 15–20% weight loss, impaired or decreased mobility, neurologic signs that interfere with normal function or reduced grooming. These limits were not exceeded in any of the experiments. Mice were injected intraperitoneally with 1 × 10^6^ LM7-GFP-ffluc osteosarcoma tumour cells. Seven days later, mice were injected intraperitoneally with 1 × 10^5^ CAR T cells. The experiment was performed twice with CAR T cells generated from two different healthy donors. For the second experiment, mice that had long-term tumour-free survival (*n* = 1 for control KO, *n* = 3 for RASA2 KO) were re-challenged with an intraperitoneal injection of 1 × 10^6^ LM7-GFP-ffluc tumor cells at day 174. Tumour burden was monitored by bioluminescence imaging as above.

### Reporting summary

Further information on research design is available in the Nature Research Reporting Summary linked to this article.

## Online content

Any methods, additional references, Nature Research reporting summaries, source data, extended data, supplementary information, acknowledgements, peer review information; details of author contributions and competing interests; and statements of data and code availability are available at 10.1038/s41586-022-05126-w.

## Supplementary information

Supplementary Figure 1 Original source images for western blots.

Reporting Summary

Statistics and reproducibility

Supplementary Table 1 Gene- and guide-level MAGeCK results for T cell suppression screens and results of shared hits analysis.

Supplementary Table 2 Results of MAGeCK analysis for condition-specific hits.

Supplementary Table 3 Guide sequences and results of arrayed screen validation.

Supplementary Table 4 Differential gene expression results for all RNA-seq analysis.

## Data Availability

All CRISPR screen data generated for this manuscript is provided in Supplementary Tables 1 and 2. Results from validation arrayed screens are detailed in Supplementary Table 3. Differential gene expression analysis is provided in Supplementary Table 4. Raw sequencing data for RNA-seq experiments is deposited on GEO with accession GSE204862. Source data are provided with this paper.

## Source data

Source Data Fig. 1
Source Data Fig. 2
Source Data Fig. 3
Source Data Fig. 4
Source Data Extended Data Fig. 1
Source Data Extended Data Fig. 2
Source Data Extended Data Fig. 3
Source Data Extended Data Fig. 4
Source Data Extended Data Fig. 5
Source Data Extended Data Fig. 6
Source Data Extended Data Fig. 7
Source Data Extended Data Fig. 8
Source Data Extended Data Fig. 9
Source Data Extended Data Fig. 10

## Code availability

Code used in this manuscript is available on Zenodo (10.5281/zenodo.6808407).
