# Supplementary material for: RASA2 ablation in T cells boosts antigen sensitivity and long-term function
Source: Nature. 2022 Aug 24;609(7925):174–82. doi: 10.1038/s41586-022-05126-w (PMC9433322; doi:10.1038/s41586-022-05126-w)
Supplement: Supplementary file 1 — Original source images for western blots. [file 41586_2022_5126_MOESM1_ESM.pdf]

nature portfolio

https://doi.org/10.1038/s41586-022-05126-w

Supplementary information

RASA2 ablation in T cells boosts antigen sensitivity and long-term function

In the format provided by the authors and unedited

Supplementary Fig. 1: Uncropped gel source data

Main Fig 2b

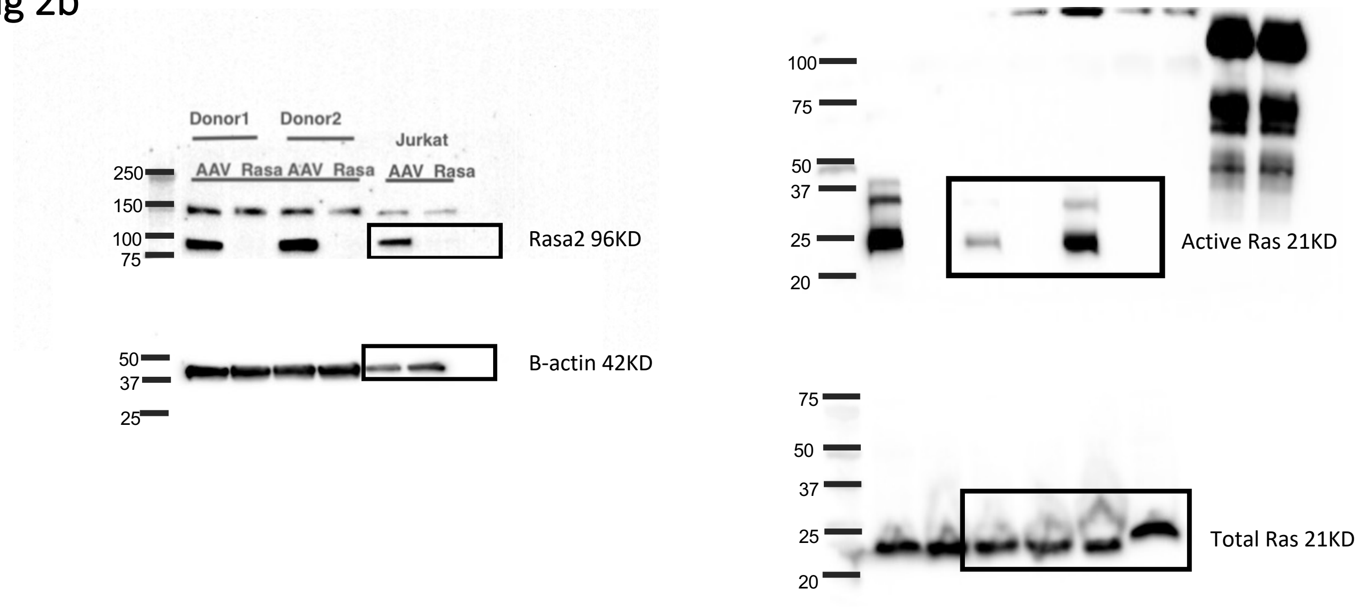

Extended Fig 2

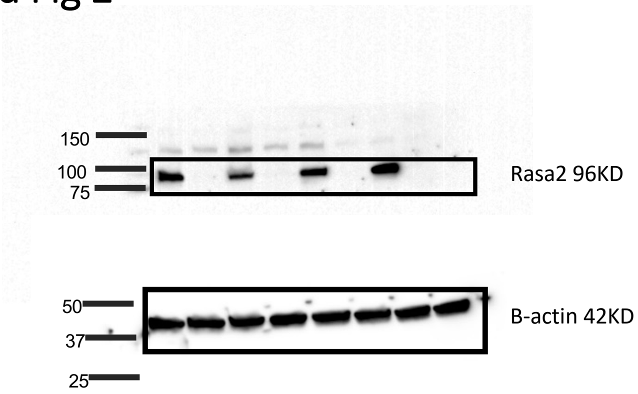

Extended Fig 3a

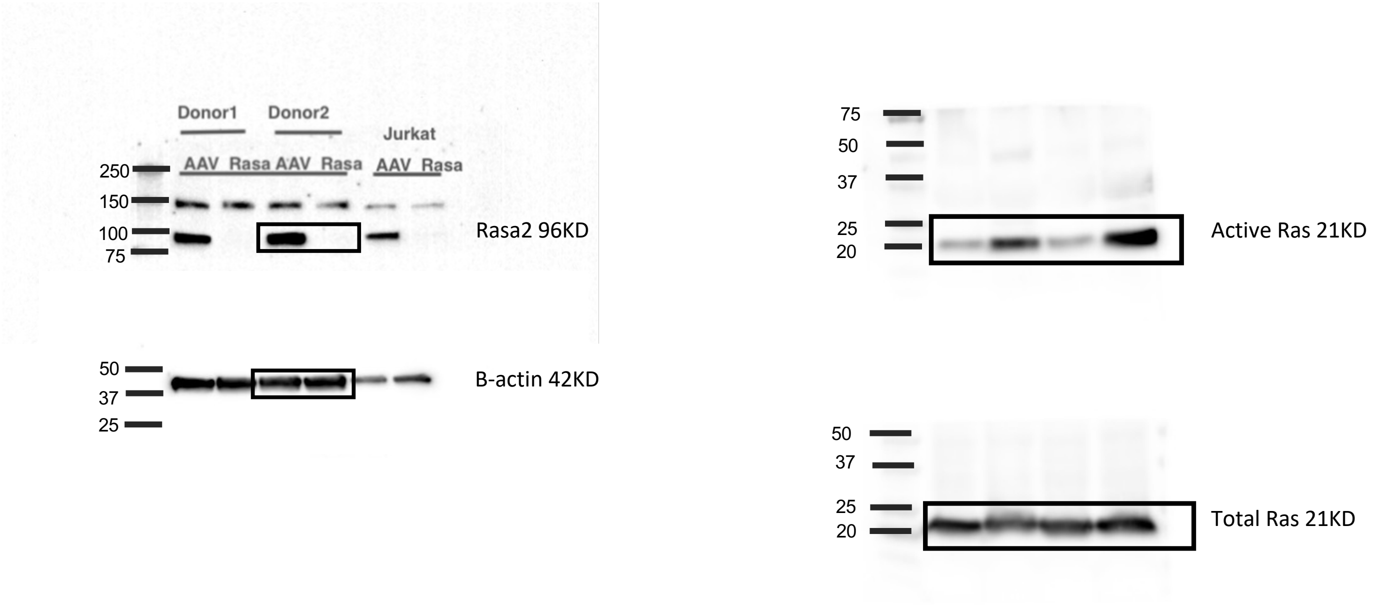

Extended Fig 3C

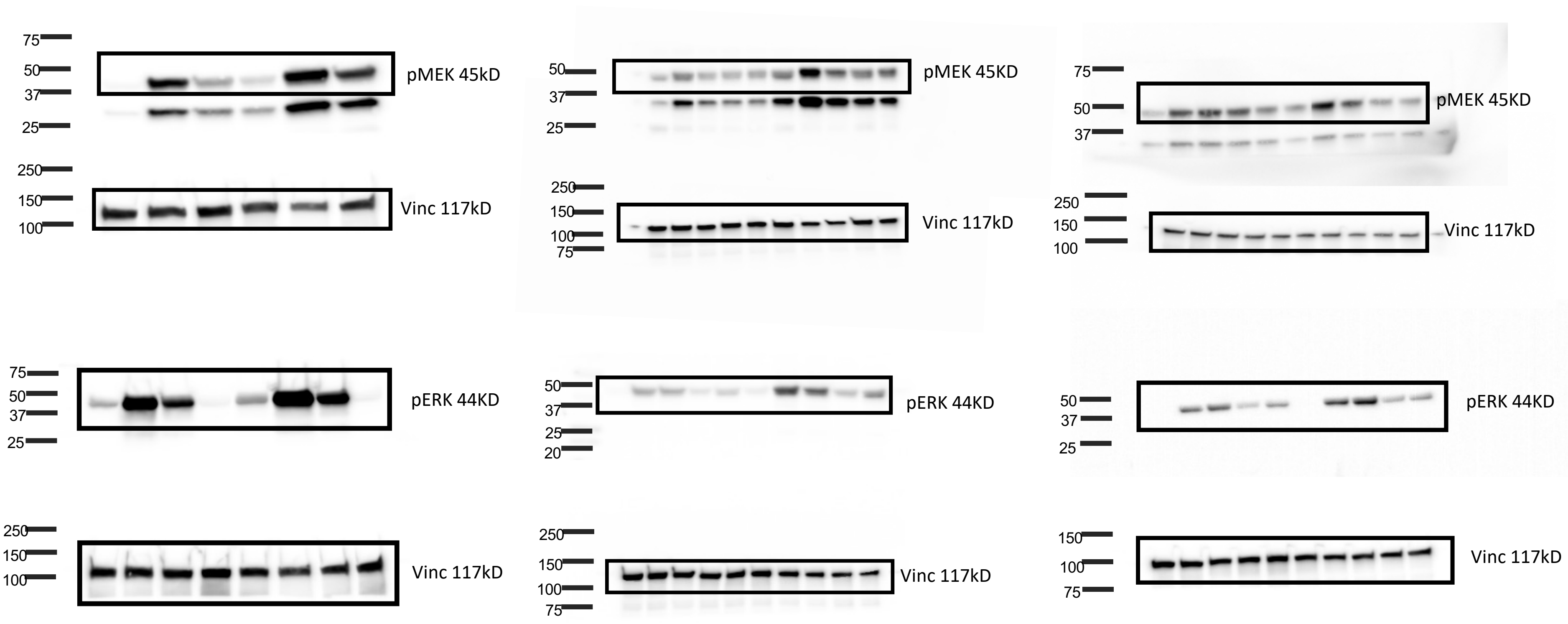

Extended Figure 5k

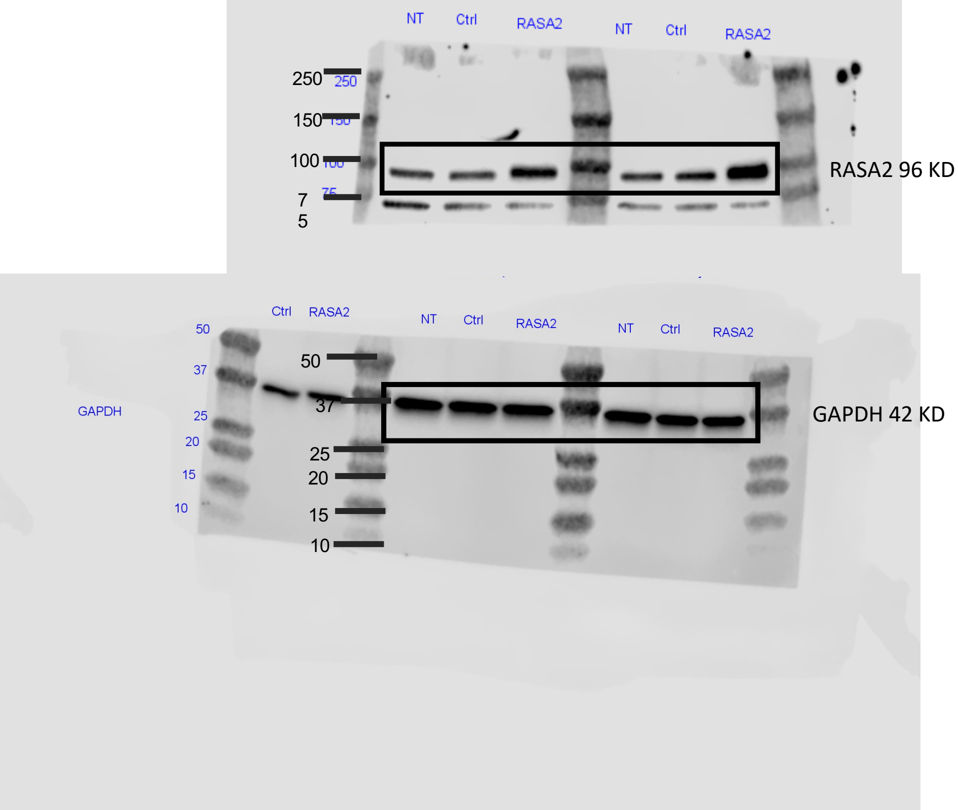

Extended Figure 6f

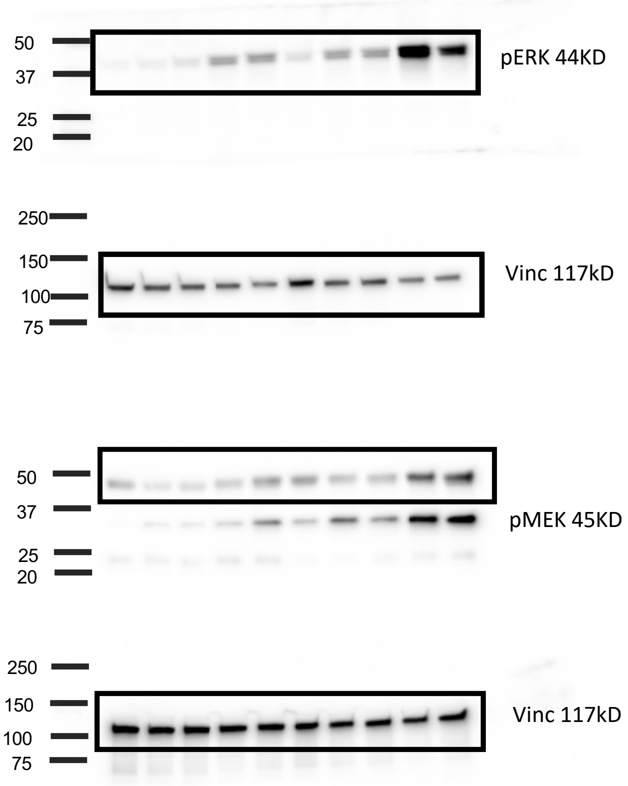

Extended Figure 10i

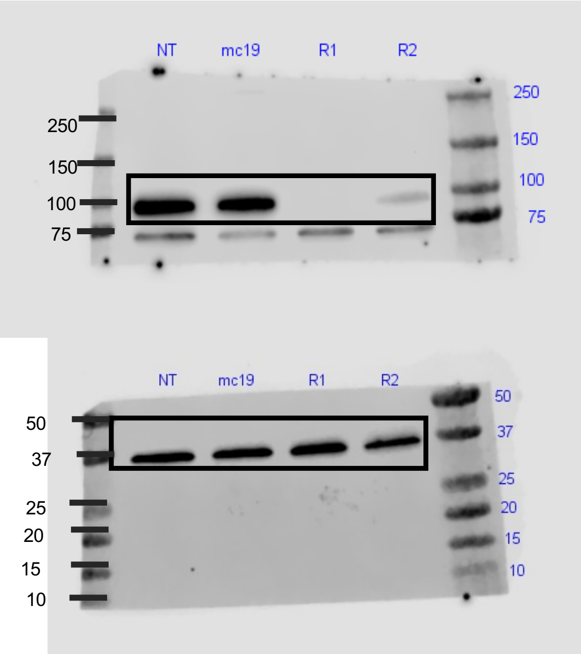

RASA2 96 KD

GAPDH 42 KD
