# Supplementary material for: RASA2 ablation in T cells boosts antigen sensitivity and long-term function
Source: Nature. 2022 Aug 24;609(7925):174–82. doi: 10.1038/s41586-022-05126-w (PMC9433322; doi:10.1038/s41586-022-05126-w)
Supplement: Supplementary file 2 — Reporting Summary [file 41586_2022_5126_MOESM2_ESM.pdf]

nature portfolio

Corresponding author(s): Alexander Marson

Last updated by author(s): 2022-08-12

Reporting Summary

Nature Portfolio wishes to improve the reproducibility of the work that we publish. This form provides structure for consistency and transparency in reporting. For further information on Nature Portfolio policies, see our Editorial Policies and the Editorial Policy Checklist.

Statistics

For all statistical analyses, confirm that the following items are present in the figure legend, table legend, main text, or Methods section.

n/a	Confirmed	
☐	☒	The exact sample size (*n*) for each experimental group/condition, given as a discrete number and unit of measurement
☐	☒	A statement on whether measurements were taken from distinct samples or whether the same sample was measured repeatedly
☐	☒	The statistical test(s) used AND whether they are one- or two-sided<br>*Only common tests should be described solely by name; describe more complex techniques in the Methods section.*
☐	☒	A description of all covariates tested
☐	☒	A description of any assumptions or corrections, such as tests of normality and adjustment for multiple comparisons
☐	☒	A full description of the statistical parameters including central tendency (e.g. means) or other basic estimates (e.g. regression coefficient) AND variation (e.g. standard deviation) or associated estimates of uncertainty (e.g. confidence intervals)
☐	☒	For null hypothesis testing, the test statistic (e.g. *F*, *t*, *r*) with confidence intervals, effect sizes, degrees of freedom and *P* value noted<br>*Give P values as exact values whenever suitable.*
☒	☐	For Bayesian analysis, information on the choice of priors and Markov chain Monte Carlo settings
☒	☐	For hierarchical and complex designs, identification of the appropriate level for tests and full reporting of outcomes
☐	☒	Estimates of effect sizes (e.g. Cohen's *d*, Pearson's *r*), indicating how they were calculated

Our web collection on statistics for biologists contains articles on many of the points above.

Software and code

Policy information about availability of computer code

Data collection	FACS Diva, BD FACS Canto II, and Attune control software was used to collect flow cytometry data. Incucyte Zoom software was used to count RFP and AnnexinV positive cell in the cancer cell killing assays. Living Image (Xenogen) version 4.5.5 software was used for acquisition of bioluminescence imaging datasets.
Data analysis	Flow cytometry data was gated with FlowJo v10 and 10.7.1 and BD FACSDiva v8. Next generation sequencing data was pre-processed with Kallisto v0.46.1. CRISPR screen results were analyzed with MAGeCK v0.5.9.The following R (v4.0.2) packages were used in this work: Plots were generated by ggplot2 v3.3.3, flow plots were generated with ggcyto 1.16.0, statistical tests by ggsignif 0.6.1, DE analysis by DESeq2 1.32.0, scRNA-seq plots were generated with Seurat 4.0.4, genes correlated with RASA2 expression in immune cells were found using correlationAnalyzeR v1.0.0. R code used in this manuscript will be made available upon reasonable request. Plots were also generated using Prism V9 (GraphPad). Densitometry analysis of western blots was completed using ImageJ 1.52q, Java 1.8.0_172. Living Image (Xenogen) version 4.5.5 software was used for analysis of the ROI values from the bioluminescence imaging.

For manuscripts utilizing custom algorithms or software that are central to the research but not yet described in published literature, software must be made available to editors and reviewers. We strongly encourage code deposition in a community repository (e.g. GitHub). See the Nature Portfolio guidelines for submitting code & software for further information.

Data

Policy information about availability of data

All manuscripts must include a data availability statement. This statement should provide the following information, where applicable:

- Accession codes, unique identifiers, or web links for publicly available datasets
- A description of any restrictions on data availability
- For clinical datasets or third party data, please ensure that the statement adheres to our policy

All CRISPR screen data generated for this manuscript is provided in Supplementary Tables 1 and 2. Results from validation arrayed screens are detailed in Supplementary Table 3. Differential gene expression analysis is provided in Supplementary Table 4. Raw sequencing data for RNA-Seq experiments is deposited on GEO with accession GSE204862. This manuscript uses the following published datasets: GSE119450, GSE89307, GSE86881 and GSE138459.

Field-specific reporting

Please select the one below that is the best fit for your research. If you are not sure, read the appropriate sections before making your selection.

☒ Life sciences ☐ Behavioural & social sciences ☐ Ecological, evolutionary & environmental sciences

For a reference copy of the document with all sections, see nature.com/documents/nr-reporting-summary-flat.pdf

Life sciences study design

All studies must disclose on these points even when the disclosure is negative.

Sample size	No statistical methods were used to predetermine sample size. Sample sizes were estimated based on preliminary experiments and previously published results. We made the effort to achieve a minimum sample size n=5 mice per treatment arm which proved to be sufficient to reproducibly observe statistically significant differences. Sample size is stated in each panel, including number of distinct human donors and technical replicates.
Data exclusions	Results show all data points collected from the experiments described in the manuscript.
Replication	All experimental findings were reproducible as indicated by the statistical analysis in the figure legends.
Randomization	For all in vivo experiments, mice were randomized to ensure equal tumor burden distribution in each group before T cells were transferred. For in vitro experiments, no randomizing was required as each experimental condition (ie: control targeting sgRNA and gene of interest sgRNAs) was controlled within each T cell donor.
Blinding	For all in vivo experiments, mouse randomization and injections were always done by a blinded member of the Preclinical Therapeutics Core or a blinded member of the Marson laboratory. Measurements of tumor burdens, monitoring of the mice, and tumor burden analysis were performed by members of the Preclinical Therapeutics Core who were blinded to the experimental groups. For the in vivo cell phenotyping and rechallenge experiments, when the Preclinical Therapeutics Core staff was not available, Dr. Carnevale collected the data and was not blinded to the groups. For the histopathological analysis of bone marrow and splenic tissues, the hematopathologist was blinded to the experimental groups. For all other in vitro experiments the data collection was not blinded, but was measured with objective methodologies (flow cytometry, incucyte, RNA-Seq). Fully blinded in vitro experiments were not possible due to personnel availability to accommodate such situations.

Reporting for specific materials, systems and methods

We require information from authors about some types of materials, experimental systems and methods used in many studies. Here, indicate whether each material, system or method listed is relevant to your study. If you are not sure if a list item applies to your research, read the appropriate section before selecting a response.

Materials & experimental systems

n/a	Involved in the study	
☐	☒	Antibodies
☐	☒	Eukaryotic cell lines
☒	☐	Palaeontology and archaeology
☐	☒	Animals and other organisms
☐	☒	Human research participants
☒	☐	Clinical data
☒	☐	Dual use research of concern

Methods

n/a	Involved in the study	
☒	☐	ChIP-seq
☐	☒	Flow cytometry
☒	☐	MRI-based neuroimaging

Antibodies

Antibodies used	For Western blots: p-ERK (Clone D13.14.4E, Cell Signaling Technology Cat #4370), p-MEK (Clone 41G9, Cell Signaling Technology Cat #9154, Vinculin (Clone VIIF9, Millipore sigma Cat #MAB3574), RASA2 (Sigma Aldrich, Cat #HPA03537), beta-Actin Rabbit mAb (HRP

March 2021

Conjugate) (Clone 13E5, Cell signaling Cat# 5125), Anti-Rabbit HRP antibody (Cell Signaling Cat# 7074), Anti-mouse IgG, HRP-linked Antibody (Cell Signaling Cat# 7076), RASA2 – rabbit anti-human GAP1m (NBP1-89794, Novus Biologicals), GAPDH – mouse anti-human GAPDH (sc-47724, clone 0411, Santa Cruz Biotechnology), goat anti–rabbit IgG–HRP (111-036-045, Jackson ImmunoResearch), goat anti–mouse IgG–HRP (sc-2005, Santa Cruz Biotechnology)

Flow Cytometry:
From Biolegend: Brilliant Violet 421™ CD69 (Clone FN50 , Cat #310930), FITC anti-human CD154 (Clone 24-31, Cat #310804), PE CD anti-human CD25 (Clone BC96 , Cat #302606), FITC anti-human CD279 (PD-1) (Clone A17188B , Cat #621612), Brilliant Violet 711™ CD223 (LAG-3) (Clone 11C3C65 , Cat #369320), Brilliant Violet 421™ anti-human CD366 (Tim-3) (Clone F38-2E2, Cat #345008), PE anti-human CD39 (Clone A1, Cat #328208), PE anti-human CD62L (Clone DREG-56 , Cat # 304806), Alexa Fluor® 488 anti-ERK1/2 Phospho (Thr202/Tyr204) (Clone 4B11B69 , Cat #675507), PE anti-ERK1/2 Phospho (Thr202/Tyr204) (Clone 6B8B69, Cat #369506), Brilliant Violet 421™ anti-RPS6 Phospho (Ser235/Ser236) (Clone A17020B, Cat #608610), PE anti-p38 MAPK Phospho (Thr180/Tyr182) (Clone A16016A, Cat #690204), Pacific Blue™ anti-human TNF-α (Clone Mab11, Cat #502920), BV785 Anti-Human CD366 (Tim-3) (Clone F38-2E2, Cat# 345032), PE CD127 (IL7Ra) (Clone A019D5, Cat# 351304), PE anti-human EGFR (Clone AY13, Cat# 352904)

From BD Biosciences:
Anti-MEK1 (pS218)/MEK2 (pS222) (Clone O24-836, Cat #562460) , PE Mouse anti-4EBP1 (pT36/pT45) (Clone M31-16, Cat #560285), Brilliant Violet 421™ Anti-Akt (pS473) (Clone M89-61, Cat #562599), PE Mouse Anti-Human IFN-γ (Clone B27, Cat #554701), BV711 Mouse Anti-Human IL-2 (Clone 5344.111, Cat #563946), APC-Cy7 Mouse anti-human CD45 (Clone 2D1, Cat# 557833), BUV395 Mouse Anti-Human CD4 (Clone SK3, Cat# 563550), BV421 Mouse Anti-Human CD62L (Clone DREG-56, Cat# 563862), BV650 Mouse Anti-Human CD45RA (Clone HI100, Cat# 563963), BV480 Mouse Anti-Human CD279 (PD-1) (Clone EH12.1, Cat# 566112), BUV737 Mouse Anti-Human
CD19 (Clone SJ25C1, Cat# 564303)

From Thermo Fisher:
PE-Cyanine7 Anti-Human CD8a (Clone SK1, Cat# 25-0087-42), PerCP-eFluor 710 Anti-Human CD223 (LAG-3) (Clone 3DS223H, Cat# 46-2239-42), 7-AAD (Cat# A1310), Counting Beads (Cat# C36995)

From Beckman Coulter:
CD19-PE (IM1285U, clone J3-119), CD19-APC (IM2470U, clone J3-119)

Validation

- BV421™ CD69 (Clone FN50) was validated here https://www.biolegend.com/en-us/products/brilliant-violet-421-anti-human-cd69-antibody-7141
- FITC anti-human CD154 (Clone 24-31) was validated here https://www.biolegend.com/en-us/products/fitc-anti-human-cd154-antibody-1665
- FITC anti-human CD279 (PD-1) (Clone A17188B) was validated here https://www.biolegend.com/en-us/products/fitc-anti-human-cd279-pd-1-antibody-18921
- PE CD anti-human CD25 (Clone BC96) was validated here https://www.biolegend.com/en-us/products/pe-anti-human-cd25-antibody-616
- BV711™ CD223 (LAG-3) (Clone 11C3C65) was validated here https://www.biolegend.com/en-us/products/brilliant-violet-711-anti-human-cd223-lag-3-antibody-14878
- BV421™ anti-human CD366 (Tim-3) (Clone F38-2E2) was validated here https://www.biolegend.com/en-us/products/brilliant-violet-421-anti-human-cd366-tim-3-antibody-7401
- PE anti-human CD39 (Clone A1) was validated here https://www.biolegend.com/en-us/products/pe-anti-human-cd39-antibody-4364
- PE anti-human CD62L (Clone DREG-56) was validated here https://www.biolegend.com/en-us/products/pe-anti-human-cd62l-antibody-653
- Alexa Fluor® 488 anti-ERK1/2 Phospho (Thr202/Tyr204) (Clone 4B11B69) was validated here https://www.biolegend.com/en-us/products/alexa-fluor-488-anti-erk1-2-phospho-thr202-tyr204-antibody-13656
- PE anti-ERK1/2 Phospho (Thr202/Tyr204) (Clone 6B8B69) was validated here https://www.biolegend.com/en-us/products/pe-anti-erk1-2-phospho-thr202-tyr204-antibody-13590
- BV421™ anti-RPS6 Phospho (Ser235/Ser236) (Clone A17020B) was validated here https://www.biolegend.com/en-us/products/brilliant-violet-421-anti-rps6-phospho-ser235ser236-antibody-18271
- PE anti-p38 MAPK Phospho (Thr180/Tyr182) (Clone A16016A) was validated here https://www.biolegend.com/en-us/products/pe-anti-p38-mapk-phospho-thr180-tyr182-antibody-18747
- anti-MEK1 (pS218)/MEK2 (pS222) (Clone O24-836) was validated here https://www.bdbiosciences.com/en-us/products/reagents/flow-cytometry-reagents/research-reagents/single-color-antibodies-ruo/alexa-fluor-647-mouse-anti-mek1-ps218-mek2-ps222.562460
- PE Mouse anti-4EBP1 (pT36/pT45) (Clone M31-16) was validated here https://www.bdbiosciences.com/en-us/products/reagents/flow-cytometry-reagents/research-reagents/single-color-antibodies-ruo/pe-mouse-anti-4ebp1-pt36-pt45.560285
- BV421™ Anti-Akt (pS473) (Clone M89-61) was validated here https://www.bdbiosciences.com/en-us/products/reagents/flow-cytometry-reagents/research-reagents/single-color-antibodies-ruo/bv421-mouse-anti-akt-ps473.562599
- Pacific Blue™ anti-human TNF-α (Clone Mab11) was validated here https://www.biolegend.com/en-us/products/pacific-blue-anti-human-tnf-alpha-antibody-4149
- PE Mouse Anti-Human IFN-γ (Clone B27) was validated here https://www.bdbiosciences.com/en-us/products/reagents/flow-cytometry-reagents/research-reagents/single-color-antibodies-ruo/pe-mouse-anti-human-ifn.554701
- BV711 Mouse Anti-Human IL-2 (Clone 5344.111) was validated here https://www.bdbiosciences.com/en-us/products/reagents/flow-cytometry-reagents/research-reagents/single-color-antibodies-ruo/bv711-mouse-anti-human-il-2.563946
- APC-Cy7 Mouse anti-human CD45 (Clone 2D1) was validated here https://www.bdbiosciences.com/en-us/products/reagents/flow-cytometry-reagents/research-reagents/single-color-antibodies-ruo/apc-cy-7-mouse-anti-human-cd45.557833
- BUV395 Mouse Anti-Human CD4 (Clone SK3) was validated here https://www.bdbiosciences.com/en-us/products/reagents/flow-cytometry-reagents/research-reagents/single-color-antibodies-ruo/buv395-mouse-anti-human-cd4.563550
- BV421 Mouse Anti-Human CD62L (Clone DREG-56) was validated here https://www.bdbiosciences.com/en-us/products/reagents/flow-cytometry-reagents/research-reagents/single-color-antibodies-ruo/bv421-mouse-anti-human-cd62l.563862
- BV650 Mouse Anti-Human CD45RA (Clone HI100) was validated here https://www.bdbiosciences.com/en-us/products/reagents/

flow-cytometry-reagents/research-reagents/single-color-antibodies-ruo/bv650-mouse-anti-human-cd45ra.563963
- BV480 Mouse Anti-Human CD279 (PD-1) (Clone EH12.1) was validated here https://www.bdbiosciences.com/en-us/products/reagents/flow-cytometry-reagents/research-reagents/single-color-antibodies-ruo/bv480-mouse-anti-human-cd279-pd-1.566112
- BUV737 Mouse Anti-Human CD19 (Clone SJ25C1) was validated here https://www.bdbiosciences.com/en-us/products/reagents/flow-cytometry-reagents/research-reagents/single-color-antibodies-ruo/buv737-mouse-anti-human-cd19.612756
- PE anti-human EGFR (Clone AY13) was validated here https://www.biolegend.com/en-us/products/pe-anti-human-egfr-antibody-7432
- BV785 Anti-Human CD366 (Tim-3) (Clone F38-2E2) was validated here https://www.biolegend.com/en-us/products/brilliant-violet-785-anti-human-cd366-tim-3-antibody-11965
- PE CD127 (IL7Ra) (Clone A019D5) was validated here https://www.biolegend.com/en-us/products/pe-anti-human-cd127-il-7ralpha-antibody-7094
- PE-Cyanine7 Anti-Human CD8a (Clone SK1) was validated here https://www.thermofisher.com/antibody/product/CD8a-Antibody-clone-SK1-Monoclonal/25-0087-42
- PerCP-eFluor 710 Anti-Human CD223 (LAG-3) (Clone 3DS223H) was validated here https://www.thermofisher.com/antibody/product/CD223-LAG-3-Antibody-clone-3DS223H-Monoclonal/46-2239-42
- 7-AAD was validated here https://www.thermofisher.com/order/catalog/product/A1310?SID=srch-srp-A1310
- Counting Beads was validated here https://www.thermofisher.com/order/catalog/product/C36995?SID=srch-hj-C36995
- RASA2 https://www.sigmaaldrich.com/US/en/product/sigma/hpa035375
- p-ERK (Clone D13.14.4E) https://www.cellsignal.com/product/productDetail.jsp?productId=4370
- p-MEK (Clone 41G9) https://www.cellsignal.com/product/productDetail.jsp?productId=9154
- Vinculin (Clone VIIF9) https://www.sigmaaldrich.com/US/en/product/mm/mab3574
-beta-Actin Rabbit mAb (HRP Conjugate) https://www.cellsignal.com/products/antibody-conjugates/b-actin-13e5-rabbit-mab-hrp-conjugate/5125
- Anti-Rabbit HRP antibody https://www.cellsignal.com/products/secondary-antibodies/anti-rabbit-igg-hrp-linked-antibody/7074
- Anti-mouse IgG, HRP-linked Antibody https://www.cellsignal.com/products/secondary-antibodies/anti-mouse-igg-hrp-linked-antibody/7076
- RASA2 – rabbit anti-human GAP1m https://www.novusbio.com/products/gap1m-antibody_nbp1-89794
- GAPDH – mouse anti-human GAPDH https://www.scbt.com/p/gapdh-antibody-0411?requestFrom=search
- goat anti–mouse IgG–HRP https://www.scbt.com/p/goat-anti-mouse-igg-hrp?requestFrom=search
- goat anti–rabbit IgG–HRP https://www.jacksonimmuno.com/catalog/products/111-036-045

Eukaryotic cell lines

Policy information about cell lines

Cell line source(s)	A375 (ATCC, CRL-1619)<br>A375-CD19 - generated in this study<br>T2 cells (ATCC, CRL-1992)<br>Nalm6 expressing luciferase and GFP, varying levels of CD19 - Gift from Justin Eyquem, Nalm6 cell line originally purchased from ATCC (CRL-3273)<br>Nalm6 expressing NY-ESO-1 - Gift from Justin Eyquem, Nalm6 cell line originally purchased from ATCC (CRL-3273)<br>LM7 - LM7 osteosarcoma cells were kindly provided to the Krenciute lab by Dr. Eugenie Kleinerman (MD Anderson Cancer Center) in 2011.<br>Jurkat reporter cells - Gift from Kole Roybal, Jurkat cells originally purchased from ATCC (Clone E6-1)<br>HEK293T - Lenti-XTM 293T Cell Line (Takara Bio Cat# 632180)
Authentication	COA were provided with cell lines from ATCC and Takara Bio. Relevant antigen expression for each cell line was routinely confirmed by flow cytometry. LM7 cells were routinely validated using the ATCC STR Profiling Cell Authentication Service.
Mycoplasma contamination	The following cell lines were tested for mycoplasma: Nalm6, A375, LM7 and 293T cells were mycoplasma free as tested using either the LookOut Mycoplasma PCR Detection Kit (Sigma Aldrich, Catalog # MP0035) at UCSF or the MycoAlert Mycoplasma Detection kit (Lonza, Catalog # LT07-218 at St. Jude). The following cell lines were used for short-term assays and not tested for mycoplasma (T2, Jurkat reporter lines). Our results pertain to the performace of primary human T cells.
Commonly misidentified lines<br>(See ICLAC register)	ICLAC registar was assessed and no commonly misidentified lines were used.

Animals and other organisms

Policy information about studies involving animals; ARRIVE guidelines recommended for reporting animal research

Laboratory animals	For experiments conducted at UCSF, 8-10 week old male or female NOD/SCID/IL2Rg (NSG) mice (Mus musculus, Strain #:005557) were purchased from JAX lab. For experiments conducted at St. Jude, NOD-SCID-Il2rg–/– (NSG) mice were obtained from breeding colonies maintained by the St. Jude Animal Resource Center. For all in vivo experiments, 8-10-weeks old mice were used. Mice allocated to different experimental groups were sex-, age-, and housing-matched.
Wild animals	This study did not involve wild animals.
Field-collected samples	No field collected samples were used.
Ethics oversight	Mice were used in accordance with guidelines established by the Institutional Animal Care and Use Committee and Laboratory Anima Resource Center at UCSF and St. Jude. Specifically, IACUC protocols used at UCSF included the UCSF Preclinical Therapeutics Core (IACUC protocol AN194778 - continuation of AN179937) and the Marson lab (AN180228-03B). IACUC protocol used at St. Jude in the

laboratory of Dr. Giedre Kreciute was 623-100650.

Note that full information on the approval of the study protocol must also be provided in the manuscript.

Human research participants

Policy information about studies involving human research participants

Population characteristics	Peripheral blood mononuclear cells (PBMCs) from anonymous healthy human donors (male and female, no age specified) were purchased fresh from StemCell Technologies or from Vitalant. PBMCs purchased from StemCell Technologies or Vitalant are produced from both male and female donors, although specific information on donor gender was not collected for the purpose of this research. New T cell donors were ordered in on a routine basis and thus experiments used different T cell donors.
Recruitment	PBMCs were purchased from two commercial soruces: StemCell Technologies and Vitalant.
Ethics oversight	PBMCs from anonymous donors were purchased from StemCell Technologies or Vitalant, which collected PBMCs from healthy donors under protocols approved by the StemCell Technologies IRB or the Vitalant IRB.

Note that full information on the approval of the study protocol must also be provided in the manuscript.

Flow Cytometry

Plots

Confirm that:

☒ The axis labels state the marker and fluorochrome used (e.g. CD4-FITC).

☐ The axis scales are clearly visible. Include numbers along axes only for bottom left plot of group (a 'group' is an analysis of identical markers).

☒ All plots are contour plots with outliers or pseudocolor plots.

☒ A numerical value for number of cells or percentage (with statistics) is provided.

Methodology

Sample preparation	For surface stains, up to 0.5 million T cells from culture were washed with PBS + 2% FBS (FACS Buffer), labeled in 100uL FACS Buffer containing the relevant antibodies, and incubated at 4C in the dark for 30 minutes. Samples were washed 2X in 1mL FACS Buffer before running. For intracellular stains, For phospho-flow cytometry assays, up to 0.5 million T cells cells were fixed with pre-warmed BD Phosflo Fix Buffer I (BD Biosciences, #557870) for 10 mins at 37°C. Cells were washed once with BD Pharmingen Stain Buffer (FBS) (Cat #554656). Next, the cells were permeabilized by adding BD Phosflow Perm Buffer III and incubated 4 hours to overnight at -20°C. Cells were then washed twice and incubated with antibodies for 30 mins at room temperature in the dark followed by two washes with BD Pharmingen Stain Buffer (FBS). For Intracellular cytokine staining, up to 0.5 million T cells were fixed and permeabilized with Fix & Perm Cell Permeabilization Kit (Thermo Fisher, Cat #Gas004) and incubated with fluorochrome-conjugated antibodies for 20 mins at RT in the dark. For Mitotracker probe staining, T cells were incubated in a 96 well plate at 200k cells per well in 25nM mitotracker Green FM (Cat #M7514) or Mitotracker Red CMXRos (Cat #M7512) in 100uL of warm X-Vivo media in the incubator for 30 minutes. Cells were then quenched with warm complete X-vivo media at a 1:1 volume, spun down, washed twice with warm X-vivo media, resuspended in 5% FBS/PBS, and then analyzed on the Attune flow cytometer. For all experiments, matched isotypes or known negatives (e.g. NT T cells) served as gating controls. LIVE/DEAD Fixable Aqua Dead Cell Stain Kit (Invitrogen) was used as a viability dye for some experiments.
Instrument	Attune NXT Cytometer (Invitrogen), BD FACS Canto II, and BD FACSAria 3 (sorting)
Software	FlowJo 10.7.1 and BD FACSDiva v8.
Cell population abundance	All sorts were end-point sorts and not for subsequent culture.
Gating strategy	For all data, viable lymphocytes were gated by FSC-A / SSC-A (as well as alive/dead marker for some experiments), and singlets by FSC-A / FSC-H. Positive populations were determined by the unstained as well as the stained/unstimulated samples. In co-culture experiments, T cells were defined as CD45+ cells. Gates and quadrants were defined based on FMO (fluorescence minus one) samples from the bone marrow samples. Gating strategy is shown in extended data.

☒ Tick this box to confirm that a figure exemplifying the gating strategy is provided in the Supplementary Information.
