# Supplementary material for: RASA2 ablation in T cells boosts antigen sensitivity and long-term function
Source: Nature. 2022 Aug 24;609(7925):174–82. doi: 10.1038/s41586-022-05126-w (PMC9433322; doi:10.1038/s41586-022-05126-w)
Supplement: Supplementary file 3 — Statistics and reproducibility [file 41586_2022_5126_MOESM3_ESM.pdf]

Statistics and Reproducibility - exact p-values and gating strategies

1.1. Fig_1f

E:T	Type	group1	group2	n1	n2	statistic	df	p
1:1	Adenosine	CTRL	RASA2	6	6	5.461613	5	0.002800
1:1	Cyclosporine	CTRL	RASA2	6	6	2.689243	5	0.043300
1:1	Tacrolimus	CTRL	RASA2	6	6	5.231174	5	0.003380
1:1	TGFb	CTRL	RASA2	6	6	4.254346	5	0.008060
1:2	Adenosine	CTRL	RASA2	6	6	4.599105	5	0.005850
1:2	Cyclosporine	CTRL	RASA2	6	6	3.280158	5	0.022000
1:2	Tacrolimus	CTRL	RASA2	6	6	9.126027	5	0.000265
1:2	TGFb	CTRL	RASA2	6	6	3.633890	5	0.015000
1:4	Adenosine	CTRL	RASA2	6	6	3.336535	5	0.020600
1:4	Cyclosporine	CTRL	RASA2	6	6	4.161547	5	0.008810
1:4	Tacrolimus	CTRL	RASA2	6	6	6.248733	5	0.001540
1:4	TGFb	CTRL	RASA2	6	6	3.718420	5	0.013700

1.2. Fig_1g

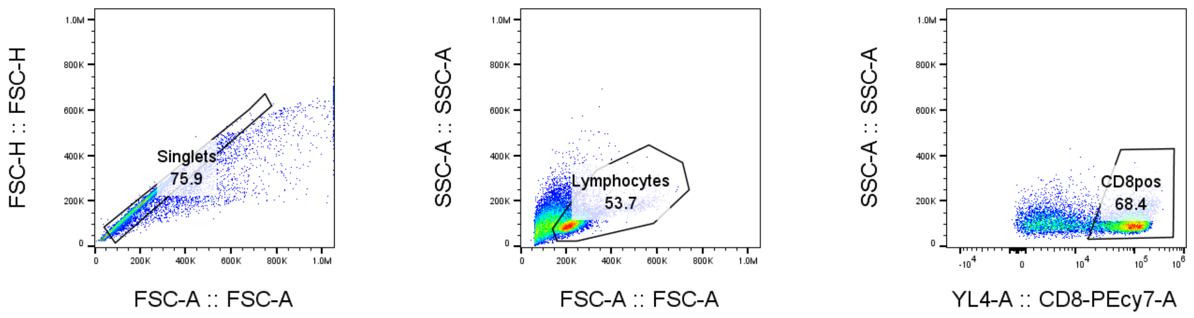

TregRatio	group1	group2	n1	n2	statistic	df	p
1to16	NT.CTRL	RASA2	4	4	-6.345588	3	0.00792
1to2	NT.CTRL	RASA2	4	4	-6.675490	3	0.00685
1to8	NT.CTRL	RASA2	4	4	-15.531412	3	0.00058

1.3. Fig_2d

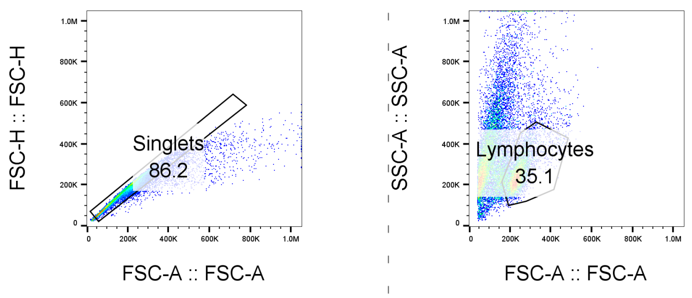

marker	group1	group2	n1	n2	statistic	p
pAKT	CTRL	RASA2	6	6	7	0.17700
pERK	CTRL	RASA2	6	6	0	0.00216
pMEK	CTRL	RASA2	6	6	0	0.00492
pS6	CTRL	RASA2	6	6	0	0.00216

1.4. Fig_2e

Gating strategy same as Fig 1g, positive gates shown in ED4a:

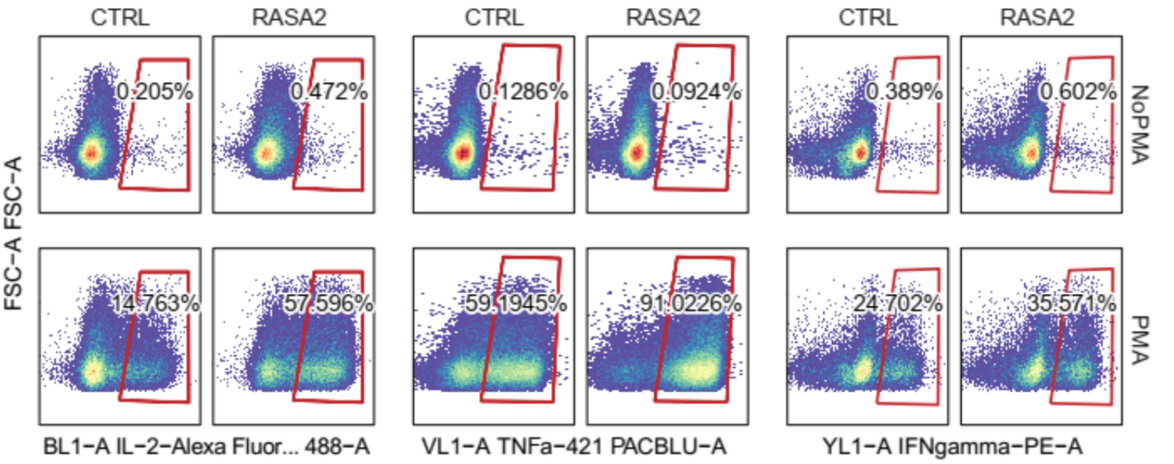

pop	group1	group2	n1	n2	statistic	p
IFNg	CTRL	RASA2	6	6	5	0.04110
IL2	CTRL	RASA2	6	6	0	0.00216
TNFa	CTRL	RASA2	6	6	0	0.00216

1.5. Fig_2i

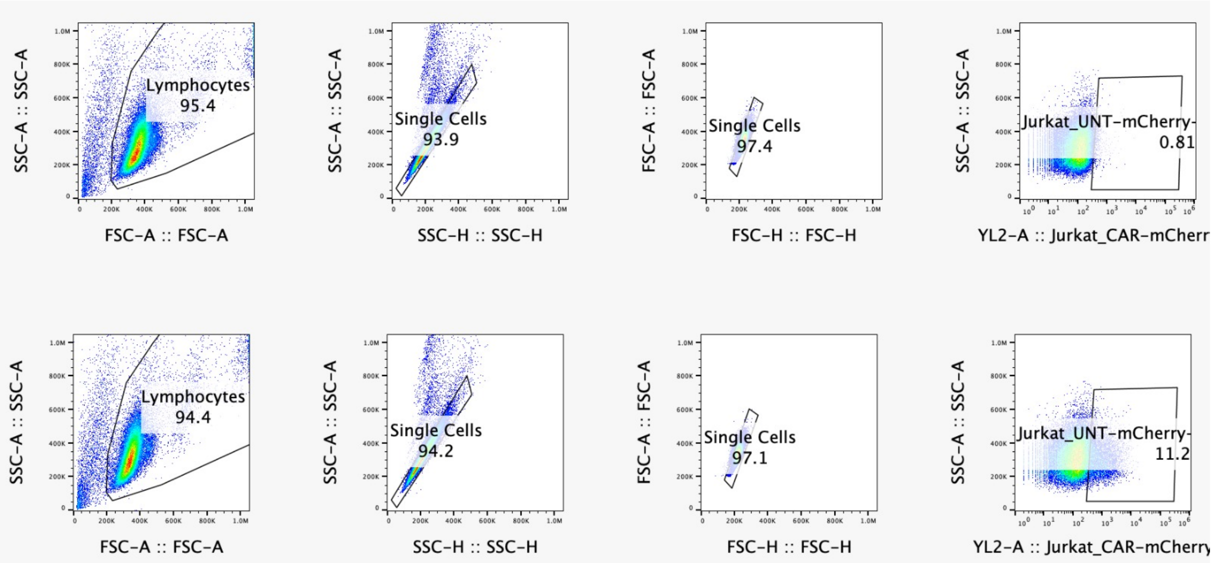

1.6. Fig_3b

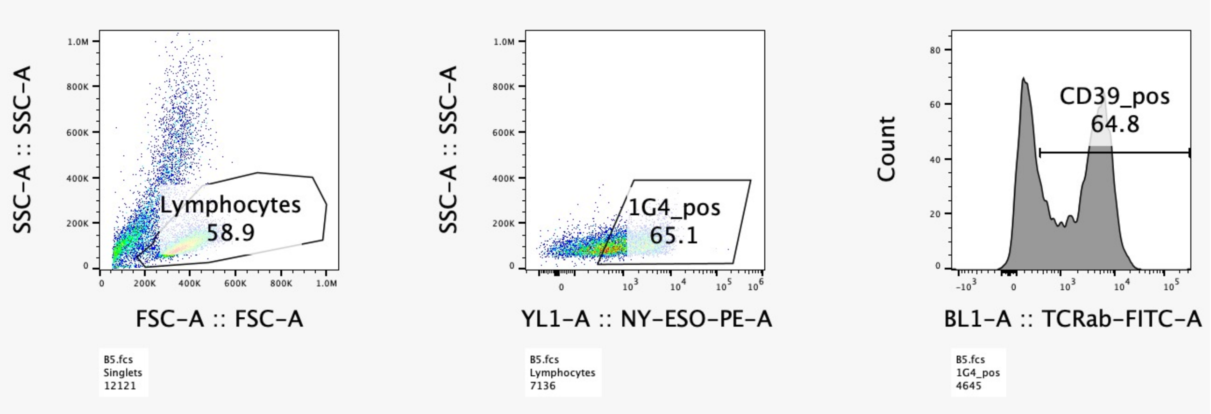

1.7. Fig_3c

Gene	group1	group2	n1	n2	statistic	p
GZMB	Stim1	Stim5	6	6	33	0.01520
IFNG	Stim1	Stim5	6	6	33	0.01520
TOX	Stim1	Stim5	6	6	0	0.00216

1.8. Fig_3f

Gating strategy same as Fig 1g, positive gates shown in ED6g:

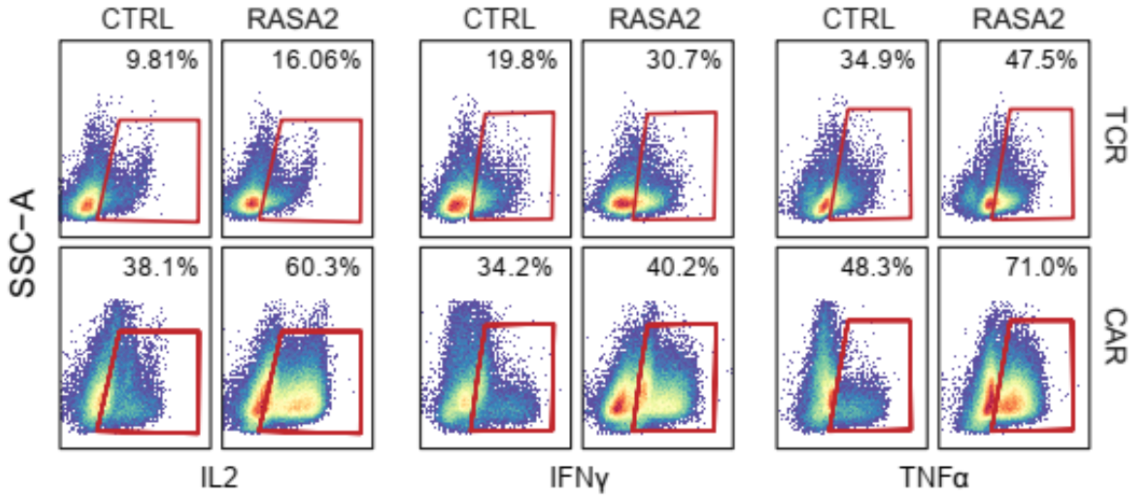

pop	group1	group2	n1	n2	statistic	df	p
IL2	CTRL	RASA2	6	6	-7.640245	8.862429	3.48e-05
IFNG	CTRL	RASA2	6	6	-3.784238	9.185392	4.16e-03
TNFa	CTRL	RASA2	6	6	-2.404329	9.286649	3.88e-02

1.9. Fig_3g

Cytokine	group1	group2	n1	n2	statistic	p
GzmB	CTRL	RASA	6	6	0	0.00216
GzmA	CTRL	RASA	6	6	5	0.04110
Granulysin	CTRL	RASA	6	6	11	0.31000
Perforin	CTRL	RASA	6	6	11	0.31000
IL-10	CTRL	RASA	6	6	0	0.00216
IFN-γ	CTRL	RASA	6	6	2	0.00866

1.10. Fig_3i

name	group1	group2	n1	n2	statistic	p
BasalResp	CTRL	RASA2	11	12	27	1.56e-02
MaximalResp	CTRL	RASA2	11	12	19	2.80e-03
SpareResp	CTRL	RASA2	11	12	7	6.66e-05

1.11. Fig_4i

Gating strategy;

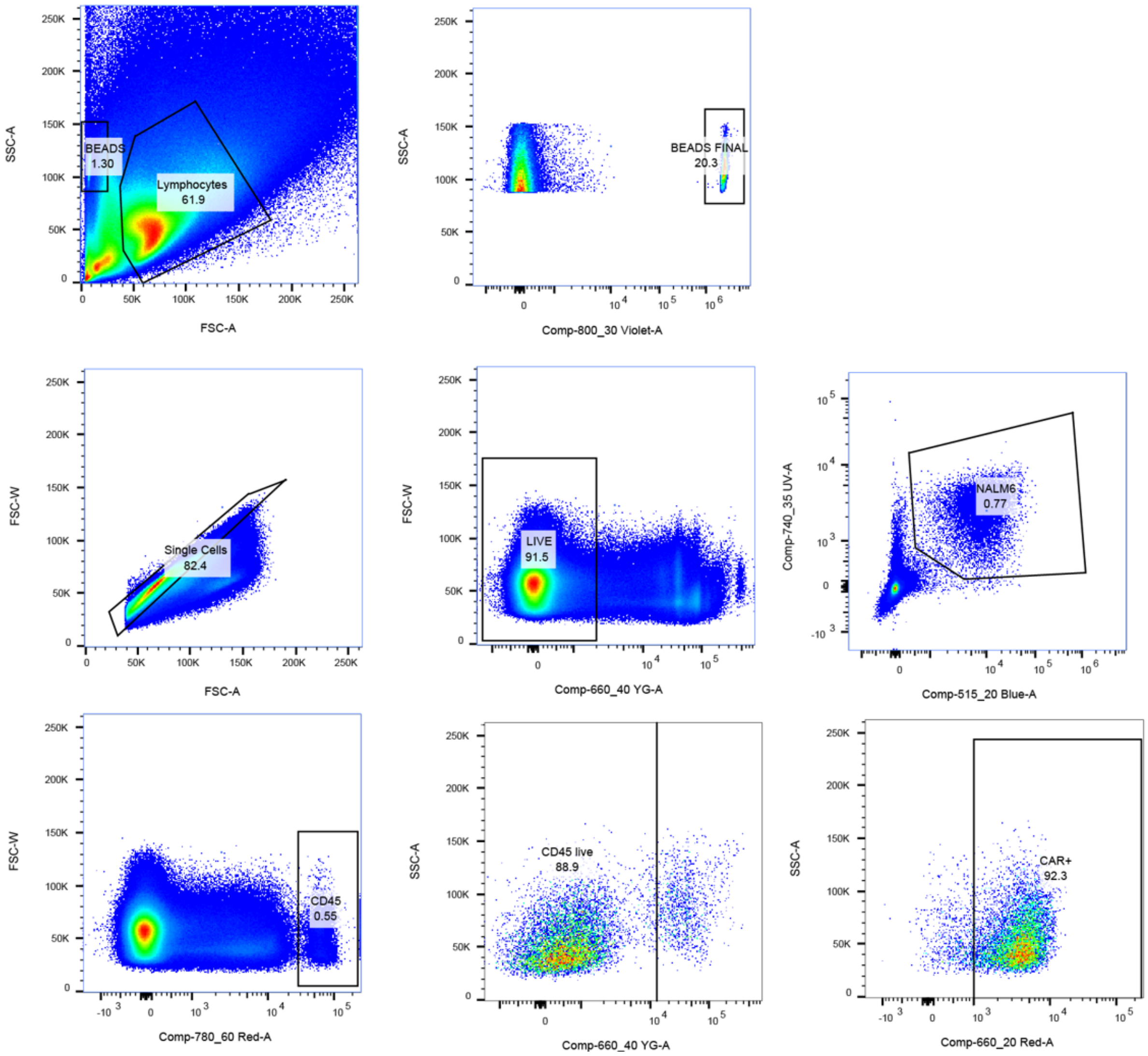

Population	Day	group1	group2	n1	n2	statistic	p
CAR	Day 16	AAVS1	RASA2	5	6	0	0.00433
CD45	Day 16	AAVS1	RASA2	5	6	0	0.00433
NALM6	Day 16	AAVS1	RASA2	5	6	29	0.00866
CAR	Day 7	AAVS1	RASA2	5	6	6	0.12600
CD45	Day 7	AAVS1	RASA2	5	6	6	0.12600
NALM6	Day 7	AAVS1	RASA2	5	6	4	0.05190

1.12. Fig_4j

Gating strategy same as Fig 4i

Day	Marker	group1	group2	n1	n2	statistic	p
D14	LAG3	CTRL	RASA2	5	6	28	0.01730
D7	LAG3	CTRL	RASA2	4	6	4	0.11400
D14	PD1	CTRL	RASA2	5	6	27	0.03030
D7	PD1	CTRL	RASA2	4	6	0	0.00952
D14	TIM3	CTRL	RASA2	5	6	27	0.03030
D7	TIM3	CTRL	RASA2	4	6	3	0.06670

1.13. Fig_4k

Gating same as Fig. 4i and:

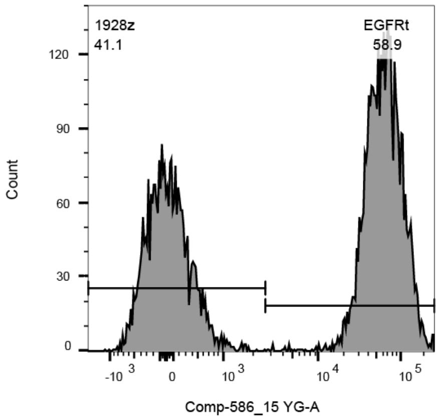

Day	group1	group2	n1	n2	statistic	p
Day 16	CTRL	RASA2	12	12	0	7.40e-07
Day 7	CTRL	RASA2	12	12	67	7.99e-01

1.14. Fig_ED2h

Tregs	group1	group2	n1	n2	statistic	p
1:1	CTRL	RASA2	8	8	2	0.000622
2:1	CTRL	RASA2	8	8	3	0.001090
4:1	CTRL	RASA2	8	8	2	0.000622
No Tregs	CTRL	RASA2	8	8	0	0.000155

1.15. Fig_ED3b

Time	group1	group2	n1	n2	statistic	df	p
0	AAVS1	RASA2	6	6	-2.9124091	5	0.033300
3	AAVS1	RASA2	6	6	-5.8234333	5	0.002110
10	AAVS1	RASA2	6	6	-7.7061730	5	0.000587
30	AAVS1	RASA2	4	4	-0.3927774	3	0.721000
60	AAVS1	RASA2	5	5	-1.1401137	4	0.318000

1.16. Fig_ED4f

T:E	Nalm6	group1	group2	n1	n2	statistic	df	p
1:0.25	V. .low	CTRL	RASA2	4	4	-5.526086	4.811813	3.00e-03
1:0.25	Low	CTRL	RASA2	6	6	-6.903946	9.347904	5.84e-05
1:0.25	WT	CTRL	RASA2	6	6	-6.826840	9.982166	4.63e-05
1:0.5	V. .low	CTRL	RASA2	4	4	-9.340107	3.697574	1.06e-03
1:0.5	Low	CTRL	RASA2	6	6	-6.591883	9.442494	8.03e-05
1:0.5	WT	CTRL	RASA2	6	6	-2.619695	7.452163	3.26e-02
1:1	V. .low	CTRL	RASA2	4	4	-11.87094	4.622244	1.23e-04
1:1	Low	CTRL	RASA2	6	6	-4.080421	9.631713	2.39e-03
1:1	WT	CTRL	RASA2	6	6	-3.547832	5.971405	1.22e-02
1:2	V. .low	CTRL	RASA2	4	4	-8.447693	5.434358	2.52e-04
1:2	Low	CTRL	RASA2	6	6	-7.020443	7.752312	1.28e-04
1:2	WT	CTRL	RASA2	6	6	-2.057215	9.944595	6.69e-02

1.17. Fig_ED6i

pop	group1	group2	n1	n2	statistic	p
CD39	AAVS	RASA2	8	8	32	1.0000
LAG3	AAVS	RASA2	8	8	25	0.5050
PD1	AAVS	RASA2	8	8	12	0.0379
TIM3	AAVS	RASA2	8	8	37	0.6450

1.18. Fig_ED7b

Type	Group	group1	group2	n1	n2	statistic	p
MITO	CAR	CTRL	RASA2	4	4	0	2.86e-02
ROS	CAR	CTRL	RASA2	4	4	0	2.86e-02
MITO	TCR	CTRL	RASA2	10	10	0	1.08e-05
ROS	TCR	CTRL	RASA2	10	10	20	2.32e-02

1.19. Fig_ED7d

Stim	name	group1	group2	n1	n2	statistic	p
repstim	BasalResp	CTRL	RASA2	6	6	0	0.00216
unstim	BasalResp	CTRL	RASA2	6	6	0	0.00216
repstim	MaximalResp	CTRL	RASA2	6	6	0	0.00216
unstim	MaximalResp	CTRL	RASA2	6	6	0	0.00216
repstim	SpareResp	CTRL	RASA2	6	6	0	0.00216
unstim	SpareResp	CTRL	RASA2	6	6	5	0.04110

1.20. Fig_ED7e

Stim	name	group1	group2	n1	n2	statistic	p
repstim	ECAR_Basal	CTRL	RASA2	6	6	0	0.00216
unstim	ECAR_Basal	CTRL	RASA2	6	6	0	0.00216
repstim	ECAR_Maximal	CTRL	RASA2	6	6	0	0.00216
unstim	ECAR_Maximal	CTRL	RASA2	6	6	18	1.00000
repstim	ECAR_Spare	CTRL	RASA2	6	6	0	0.00216
unstim	ECAR_Spare	CTRL	RASA2	6	6	36	0.00216

1.21. Fig_ED7f

Target	Stim	name	DFn	DFd	F	p	ges
CTRL	repstim	BasalResp	3	19	1.718	1.97e-01	0.213
CTRL	repstim	MaximalResp	3	19	21.356	2.64e-06	0.771
CTRL	repstim	SpareResp	3	19	23.164	1.46e-06	0.785
RASA2	repstim	BasalResp	3	19	1.664	2.08e-01	0.208
RASA2	repstim	MaximalResp	3	19	0.592	6.28e-01	0.085
RASA2	repstim	SpareResp	3	19	1.327	2.95e-01	0.173
CTRL	unstim	BasalResp	3	19	2.933	6.00e-02	0.317
CTRL	unstim	MaximalResp	3	19	0.656	5.89e-01	0.094
CTRL	unstim	SpareResp	3	19	1.575	2.28e-01	0.199
RASA2	unstim	BasalResp	3	19	5.332	8.00e-03	0.457
RASA2	unstim	MaximalResp	3	19	1.418	2.69e-01	0.183
RASA2	unstim	SpareResp	3	19	2.511	9.00e-02	0.284

1.22. Fig_ED9j

Gating strategy is as in Fig 4i and:

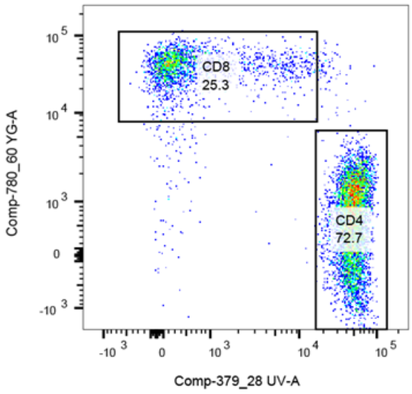

Pop	Day	group1	group2	n1	n2	statistic	p
CD4+	Day 16	AAVS1	RASA2	5	6	19	0.537000
CD4+	Day 7	AAVS1	RASA2	10	12	0	0.000083
CD8+	Day 16	AAVS1	RASA2	5	6	8	0.247000
CD8+	Day 7	AAVS1	RASA2	10	12	116	0.000242

1.23. Fig_ED9k

Gating strategy is as in Fig ED9j and:

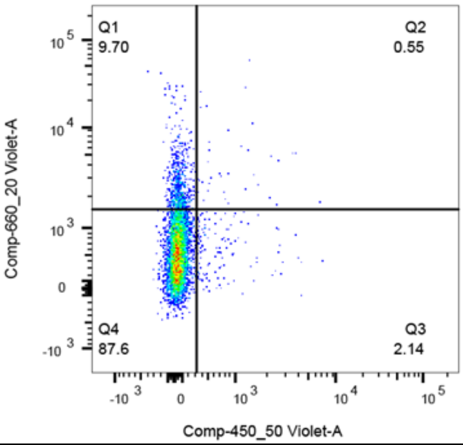

Subset	Day	Pop3	group1	group2	n1	n2	stat	p
CD4	16	CD62L+CD45RA+	CTRL	RASA2	6	6	29	0.09310
CD4	16	CD62L+CD45RA-	CTRL	RASA2	5	6	29	0.01350
CD4	16	CD62L-CD45RA-	CTRL	RASA2	6	6	9	0.17300
CD4	16	CD62L-CD45RA+	CTRL	RASA2	6	6	16	0.81800
CD8	16	CD62L+CD45RA+	CTRL	RASA2	6	6	36	0.00216
CD8	16	CD62L+CD45RA-	CTRL	RASA2	6	6	36	0.00477
CD8	16	CD62L-CD45RA-	CTRL	RASA2	6	6	33	0.01520
CD8	16	CD62L-CD45RA+	CTRL	RASA2	6	6	1	0.00433
CD4	7	CD62L+CD45RA+	CTRL	RASA2	5	6	18	0.66200
CD4	7	CD62L+CD45RA-	CTRL	RASA2	5	6	25	0.08230
CD4	7	CD62L-CD45RA-	CTRL	RASA2	5	6	22	0.24700
CD4	7	CD62L-CD45RA+	CTRL	RASA2	5	6	20	0.42900
CD8	7	CD62L+CD45RA+	CTRL	RASA2	5	6	23	0.17700
CD8	7	CD62L+CD45RA-	CTRL	RASA2	5	6	27	0.03030

Subset	Day	Pop3	group1	group2	n1	n2	stat	p
CD8	7	CD62L-CD45RA-	CTRL	RASA2	5	6	8	0.24700
CD8	7	CD62L-CD45RA+	CTRL	RASA2	5	6	13	0.79200

Statistics and Reproducibility - experimental design and details

Figure 1

b) n=4 human donors for the Stimulation only and Tregs screen, n=2 for the Adenosine, Cyclosporine and Tacrolimus screens, and n=1 for the TGFβ screen. Statistics for each screen were derived using MAGeCK software which utilized robust rank aggregation for gene level analyses. Data for this analysis can be found in Supplementary Table 1.
e-f) n=2 human T cell donors in triplicates. This experiment was performed twice with similar results.
g) This experiment was performed once in n=4 independent human T donors.
h) Data shown for one representative donor out of four independent T cell donors with similar results.

Figure 2

b) Experiment performed twice with similar results.
c-f) n=2 human T cell donors in triplicates. Experiments were performed at least twice with similar results.
g) n=2 human T cell donors in duplicate. Experiments were performed once.
h) Data shown for one representative donor out of two independent T cell donors. Experiment was performed once.
i) Experiment was performed once.
j-k) n=4 human T cell donors, experiment was performed once.

Figure 3

b) This experiment was performed once in n=4 independent human T donors.
c,d) This experiment was performed once in n=3 independent human T donors with the NY-ESO-1 TCR, and once in n=3 independent human T donors with the CD19-specific CAR.
e) Data representative of one T cell donor, was repeated in at least 8 T cell donors across 4 independent experiments with similar results.
f) n=2 human T cell donors in triplicate. This experiment was repeated three times with similar results.
g) This experiment was performed once in n=3 independent human T donors.
h-i) This experiment was performed with n=2 human T cell donors in 6 technical replicates. This experiment was performed twice with similar results.
j-l) Data in (j) is for one donor and for (k) is for two donors, and (l) is data from 7 different human T cell donors aggregated from three independent experiments.
m) Data in is for one representative donor in triplicates. This experiment was performed in 3 different T cell donors (summary statistics available in Extended data 8c). This experiment was repeated twice with similar results.

Figure 4

b) n=6 biologically independent mice per group. This experiment was performed once.
d) n=5 mice with RASA2 KO T cells, n=4 for CTRL T cells. This experiment was performed once.
f,g) n=7 mice per group. This experiment was performed twice with two separate donors with similar results.
i-j) Day 7: n=5 for CTRL, n=6 for RASA2, Day 16: n=6 for CTRL, n=6 for RASA2. This experiment was performed once.

k) Day 7: n=6 for “mix 1” (triangles), n=6 for “mix 2” (circles), Day 16: n=6 for “mix 1”, n=6 for “mix 2”. This experiment was performed once.
m-o) Quantitative bioluminescence imaging (mean ± SEM, n=10 for CTRL, n=14 for RASA2, *p < 0.05 for two-tailed paired Student’s t-test). n, Representative bioluminescence images of each treatment group. (o) Survival curve for the LM7 cohort in (m) (n=10 for CTRL; n=14 for RASA2 group; exact p-value by log-rank test). This experiment was performed twice with two separate donors with similar results.

Extended Data Figure 1
a-e) n=4 human donors for the Stimulation only and Tregs screen, n=2 for Adenosine, Cyclosporine and Tacrolimus screens, and n=1 for the TGFβ screen. Statistics for each screen were derived using MAGeCK software which utilized robust rank aggregation for gene level analyses. Data for this analysis can be found in Supplementary Table 1.
f) n=2 human T cell donors, 2 sgRNAs per gene target in triplicates. This experiment was performed once.

Extended Data Figure 2
a) n=4 human T cell donors. This experiment was performed at least 5 times with similar results.
b) n=2 human T cell donors. This experiment was performed three times with similar results.
c) Data from one representative donor out of 2 human T cell donors tested in triplicate. This experiment was performed twice with similar results.
d) n=2 donors in 3 replicates. This experiment was performed twice with similar results.
e) Data from one representative donor with 3 technical replicates. This experiment was performed in 2 human T cell donors twice with similar results.
f,h) This experiment was performed once in n=4 independent human T donors. Data from all four donors is shown.

Extended Data Figure 3
a) Experiment performed once.
b,c) Data for 3 different human T cell donors. This experiment was performed twice with similar results.
d,e) n = 2 human T cell donors. This experiment was performed twice with similar results.
f) n = 2 human T cell donors. This experiment was performed twice with similar results.
g) Representative data for one of 4 human T cell donors for p-ERK and CFSE, then data shown for all 4 human T cell donors for CD69. n = 4 human T cell donors. This experiment was performed twice with similar results.

Extended Data Figure 4
a) Representative data for one of two donors. This experiment was repeated 3 times with similar results.
b) Left: n = 2 human T cell donors. This experiment was repeated three times with similar results. Middle: n = 2 human T cell donors, experiment was performed twice with similar results. Right: n = 2 human T cell donors, experiment was performed twice with similar results.
d) n = 2 human T cell donors. This experiment was repeated at least 5 times with similar results.
e,f) n = 2 human T cell donors. Experiment was performed once.

Extended Data Figure 5
a) Experiment was performed once.
b-d) n = 4 human T cell donors. This experiment was performed once.
k) n= 2 human T cell donors. This experiment was performed twice with similar results.
l) n= 3 human T cell donors. This experiment was performed in the 3 T cell donors across three independent experiments.
m) representative data from one human T cell donor out of two, which are shown in (n).
n) n=2 human T cell donors. This experiment was performed twice with similar results.

Extended Data Figure 6
a) n=4 T cell donors, this experiment was performed once.
b) n=3 T cell donors, this experiment was performed once.
c) n=4 T cell donors, this experiment was performed at least twice with similar results.
d,e) Representative data from two T cell donors. n=2 T cell donors in (e). This experiment was performed at least three times with similar results.
f) Experiment was performed once.
g) Data from one representative donor out of two. This experiment was performed at least three times with similar results.
h) n = 2 human T cell donors. This experiment was performed at least three times with similar results.
i) n = 4 human T cell donors. This experiment was performed at least three times with similar results.

Extended Data Figure 7
a) n=3 human T cell donors. Experiment was performed once.
b) n=2 human T cell donors. Experiment was performed twice with similar results.
c-e) Data shown for one of two T cell donors. Experiment was performed twice with similar results.
f) Experiment performed once.

Extended Data Figure 8
a) Representative data for one human donor out of 7 different human T cell donors from three independent experiments (summarized in Figure 3l).
b) Experiment repeated at least three times with similar results.
c) n=3 human T cell donors. This experiment was repeated twice with similar results.
d) Representative data from one of the donors described in (c).
e) Representative data for one of two human T cell donors co-cultured with Nalm6 leukemia cells. Experiment was performed twice with similar results.
f) Representative data for one of two human T cell donors summarized in (g).
g) n=2 human T cell donors. This experiment was performed once.

Extended Data Figure 9
a,b) n=6 biologically independent mice per group. This experiment was performed once.
c,d) n=5 mice with RASA2 KO T cells, n=4 for CTRL T cells. This experiment was performed once.
e) Representative data from two human T cell donors. This experiment was performed twice with similar results.

f,g) n=7 mice per group. This experiment was performed twice with two separate donors with similar results.
h,i) n=8 mice per group for control-edited CAR-T cells and n=7 mice per group for RASA2 edited CAR-T cells. This experiment was performed twice with two separate donors with similar results.
j,k) Day 7: n=5 for CTRL, n=6 for RASA2, Day 16: n=6 for CTRL, n=6 for RASA2, error bars mean ± SEM. This experiment was performed once.
l) representative gating data for (m).
m) Day 7: n=6 for CTRL, n=6 for RASA2, Day 16: n=6 for CTRL, n=6 for RASA2. This experiment was performed once.

Extended Data Figure 10
a-c) n=5 in no T cell control arm, and n=7 mice per arm in each cohort. This experiment was performed once. Survival analysis for the leukemia rechallenge model of the cohort shown in (a,b). Exact p-value by log-rank test.
d) n=2 human donors, n=3 mice per group. This experiment was performed once.
e,f) For mice receiving only CAR-T cells (n=2 human donors, n=3 mice per group), as well as mice receiving tumor-clearing CAR-T cell infusions (n=1 human donor, n=6 mice per group. This experiment was performed once. (f) shows representative H&Es from bone marrow and spleens of mice from (e).
h) n=10 for CTRL, n=14 for RASA2. This experiment was performed twice with two separate donors with similar results.
i) n=2 human T cell donors. This experiment was performed 5 times with similar results.
j) n=10 for CTRL, n=14 for RASA2. Experiment was performed twice with similar results.
k) Mice without detectable BLI from experiment in (j), which include n=1 no T cell control, n=1 CTRL, and n=3 RASA2 KO CAR-T cell condition, were re-challenged with a second tumor injection. This experiment was performed twice with similar results.
